# Systematic discrimination of the repetitive genome in proximity of ferroptosis genes and a novel prognostic signature correlating with the oncogenic lncRNA CRNDE in multiple myeloma

**DOI:** 10.3389/fonc.2022.1026153

**Published:** 2022-12-20

**Authors:** Jiading Qin, Amit Sharma, Yulu Wang, Fabian Tobar-Tosse, Tikam Chand Dakal, Hongde Liu, Hongjia Liu, Bo Ke, Chunfang Kong, Tingting Liu, Chunxia Zhao, Ingo G. H. Schmidt-Wolf, Chenghao Jin

**Affiliations:** ^1^ Medical College of Nanchang University, Nanchang, China; ^2^ Department of Hematology, Jiangxi Provincial People’s Hospital, Nanchang, China; ^3^ National Clinical Research Center for Hematologic Diseases, The First Affiliated Hospital of Soochow University, Soochow, China; ^4^ Department of Integrated Oncology, Center for Integrated Oncology, University Hospital of Bonn, Bonn, Germany; ^5^ Department of Neurosurgery, University Hospital of Bonn, Bonn, Germany; ^6^ Department of Basic Sciences for Health, Pontificia Universidad Javeriana Cali, Cali, Colombia; ^7^ Genome and Computational Biology Lab, Department of Biotechnology, Mohanlal Sukhadia University, Udaipur, India; ^8^ State Key Laboratory of Bioelectronics, School of Biological Science & Medical Engineering, Southeast University, Nanjing, China; ^9^ School of Nursing, Nanchang University, Nanchang, China

**Keywords:** ferroptosis, lncRNA – long noncoding RNA, repetitive genome, multiple myeloma, gene signature, prognosis

## Abstract

Emerging insights into iron-dependent form of regulated cell death ferroptosis in cancer have opened a perspective for its use in cancer therapy. Of interest, a systematic profiling of ferroptosis gene signatures as prognostic factors has gained special attention in several cancers. Herein, we sought to investigate the presence of repetitive genomes in the vicinity of ferroptosis genes that may influence their expression and to establish a prognostic gene signature associated with multiple myeloma (MM). Our analysis showed that genes associated with ferroptosis were enriched with the repetitive genome in their vicinity, with a strong predominance of the SINE family, followed by LINE, of which the most significant discriminant values were SINE/Alu and LINE/L1, respectively. In addition, we examined in detail the performance of these genes as a cancer risk prediction model and specified fourteen ferroptosis-related gene signatures, which identified MM high-risk patients with lower immune/stromal scores with higher tumor purity in their immune microenvironment. Of interest, we also found that lncRNA CRNDE correlated with a risk score and was highly associated with the majority of genes comprising the signature. Taken together, we propose to investigate the molecular impact of the repetitive genome we have highlighted on the local transcriptome of ferroptosis genes in cancer. Furthermore, we revealed a genomic signature/biomarker related to ferroptosis that can be used to predict the risk of survival in MM patients.

## Highlights

- Ferroptosis-related genes showed enrichment with the repetitive genome in their vicinity, with strong predominance of the SINE family.
- We generated ferroptosis-related prognostic gene signature that can identify high-risk multiple myeloma patients.
- LncRNA CRNDE showed strong association with the majority of genes forming the prognostic signature.

## Introduction

Cell death is an essential feature of physiological/pathological processes, and ferroptosis which differs considerably from other cell death types, such as apoptosis, necrosis, and autophagy has recently gained attention. Accumulative studies have shown that dysregulated ferroptosis participates in several cancers, including renal cell carcinoma (1), colorectal carcinoma (2), gastric cancer (3), and multiple myeloma (4, 5). Overall, targeting potential regulatory factors in the ferroptosis pathway is thought to promote or inhibit disease progression in several malignancies.

Over the years, ferroptosis-related genes have been used to generate a prognostic signature in lung adenocarcinoma (6), low-grade glioma (7)., acute myeloid leukemia (8), gastric cancer (9), renal cell carcinoma (10), osteosarcoma (11), skin melanoma (12), and breast cancer (13). However, most of studies focused on the gene expression patterns and correlation with the clinical outcomes, mainly survival rate. None of the above-mentioned studies addressed the impact of genome organization in proximity to these genes, which is known to play a pivotal role in human diseases (14). On this note, the prevalence of repetitive sequences, especially LINEs (Long Interspersed Nuclear Elements), SINEs (Short Interspersed Nuclear Elements), Alu family in the functional parts of genomes and their association with cancers remains undisputed (15–17). Aoki et al. evaluated global methylation levels of four repetitive elements (LINE-1, Alu Ya5, Alu Yb8 and Satellite-α) in MM samples and found the global hypomethylation of LINE-1 being associated with progression and worse prognosis of multiple myeloma (MM) (18). Using bisulfite treatment followed by sequencing, Bollati et al. investigated the methylation status of repetitive DNA elements to verify a possible correlation with the different molecular subtypes of MM, and found a progressive and significant decrease of methylation in Alu, LINE-1 and SAT-α sequences (19). In a comprehensive study, Lee et al. discussed about various somatic insertions of LINE-1, Alu and ERV in different types of cancer, including colorectal, glioblastoma, ovarian, prostate and multiple myeloma (20). It is also noteworthy to mention that some noncoding RNAs (ncRNAs), particularly long noncoding RNAs, have been found to be involved in biological processes of ferroptosis, thus influencing cancer growth (21, 22). Although the exact regulatory mechanism behind this remains unclear, their potential use as ncRNAs-based ferroptosis targeting has been hypothesized (23) An interesting study examined some lncRNAs closely related to ferroptosis and identified PELATON as a novel ferroptosis suppressor that may also serves as a prognostic signature in glioblastoma patients (24).

Considering this, herein, we investigate the presence of repetitive genomes in the vicinity of ferroptosis genes that may influence their expression. In our comprehensive approach, we considered the analysis of various repeat configurations, e.g., LINEs (L1 and L2), SINEs (Alu and MIR), low complexity (AT and GC) and interspersed elements, across the upstream promoter region of these particular genes, as we reported previously (25). In addition, we used ferroptosis genes to create the first prognostic gene signature (based on risk groups and immune microenvironment) linked to multiple myeloma. Besides, we demonstrated the putative association of the oncogenic lncRNA CRNDE with the obtained MM-specific ferroptosis gene signature.

## Materials and methods

### Ferroptosis-related genes and repetitive genome analysis

We manually collected 387 genes classified as ferroptosis driver genes, suppressor genes, and markers using available database (http://www.zhounan.org/ferrdb). Upon removal of duplicates, we retained 269 genes that were used for further analysis like repetitive genomic analysis and determination of the prognostic signature.

To identify repetitive genomic sequence in the proximity of these genes, we retrieved repeats and genomic annotations from the T2T genome assembly (CHM13v2.0) available in the NCBI FTP (ftp://ncbi.nlm.nih.gov). Genomics coordinates were used to identify repeats in the 2kb promoter region of genes, as well as the classification of the repeats, their length, and their order relative to the transcription start site. We then calculated the repeat content for each gene using the n-gram probabilistic model (26) in which each repeat was defined as a unigram with a normalized weight (frequency and length were properties associated with the model). It was calculated as RC(r) = *Log* (*Nr* * *Lr/Le*) , where the repeat content (RC) for the repeat (r) is defined by the absolute frequency (Nr) and repeat length (Lr) under a region of exploration with a specific length (Le), here 2kb promoter. A weighted matrix was created with the genes as the index and the repeat content values as the score for each repeat type. To identify discriminative repeats, two clustering methods were applied under the matrix: Hierarchical clustering with a complete linkage method, Manhattan distance metric for the whole data, and k-mean clustering as an unsupervised algorithm for the pair of repeat types with a significant discriminative score defined by hierarchical clustering, mainly implemented by using custom Python algorithms. Finally, gene clusters with associated repeats in k-means clustering were described by functional enrichment analysis in the String database (https://string-db.org), which includes Geneontology, KEGG, and Wikipathways datasets, together with the Enrich Tool and Allen Brain Atlas datasets (27).

### Gene expression data and construction/validation of a prognostic signature

Multiple myeloma was selected to establish the prognostic signature based on ferroptosis-related genes. MM gene expression study MMRF-COMMPASS was obtained from the XENA database maintained by UCSC (https://xenabrowser.net/datapages/) and GSE24080 was obtained from the GEO database (https://www.ncbi.nlm.nih.gov/geo/). We used the MICE package to supplement missing values such as ethnicity, race, age, and International Staging System (ISS) stage from the MMRF-COMMPASS study and B2M, CRP, and creatinine from the GSE24080 cohort. We strictly followed the previously described procedure for data processing, and the paired-samples t-test was used to check for the consistency of the distribution between two cohorts. Subsequently, 844 patients from the MMRF-COMMPASS study were used as the training cohort, while the 556 patients from the GSE24080 dataset served as the validation cohort. Next, Kaplan-Meier and univariate Cox analyses were performed in the training cohort to investigate the prognostic relationship between gene expression and overall survival (OS). A list of prognostic genes with risk correlation coefficients was generated using the Cox regression model LASSO (Least Absolute Shrinkage and Selection Operator) based on the OS in the training cohort with the optimal parameter lambda. Risk score was calculated using following equation, where n, βi and Coefi represented the number of hub genes, regression coefficient values and gene expression levels, respectively. Risk Score = $\sum_{i=1}^{n}Coefi\;*\;\beta i$ . On the basis of median risk scores, patients were divided into either high or low risk score groups and Kaplan-Meier analysis were used to determine the survival differences between them. In this study, ROC analyses were performed to further evaluate the prognostic power of the ferroptosis-related gene signature. A similar procedure was used for the validation cohort. To mention, the training cohort had only OS status with five years follow-up, while validation cohort contained both OS status and event-free survival (EFS) status with seven years follow-up. We extend our analysis by generating and validating the nomogram-based analysis for predicting the survival probability in our cohorts. The nomogram was validated with the R package “rms” (calibrated for 3 and 5 years) and the C-index was measured to determine the predictive power.

### Gene set enrichment analysis and immune infiltration status estimation

The relative cell component of tumor microenvironment in the MMRF-COMMPASS study was calculated using the CIBERSORT algorithm (28, 29). Furthermore, gene set enrichment analysis (GSEA) were used to investigate the pathophysiological mechanisms associated with the ferroptosis-related genes. Based on the median cutoff value, samples were divided into low and high expression groups. KEGG enrichment terms with an adjusted P value<0.05 and false discovery rate (q value)<0.05 were considered statistically significant and ranked accordingly. ESTIMATE algorithm was used to calculate the immune score, stromal score, and tumor purity of each sample. We also quantified the relative infiltration of 28 immune cell types that mark the infiltrating immune cells of MM by single-sample GSEA analysis (ssGSEA) followed previous published methods (30). Each immune cell type of feature gene panels was obtained from a recent article (31). An enrichment score in ssGSEA analysis represented the relative abundance of each immune cell type *via* “GSVA” package (version 1.39.1).

### Expression profile of ferroptosis-related signatures in pan-cancer

To explore the expression of obtained signatures in pan-cancer, we downloaded the gene expression data of FPKM from TCGA for 33 cancers, FPKM (Fragments Per Kilobase per Million) i.e. fragments per kilobase of transcription per million mapped reads. We then calculated the average expression levels of these selective genes in all samples and in each cancer type separately to plot heat maps for visualization.

### Prediction of ferroptosis and lncRNA interactions

LncRNAs gene expression in myeloma were obtained from MMRF-COMMPASS dataset in XENA database maintained by UCSC (https://xenabrowser.net/datapages/) and GSE24080 dataset in GEO database (https://www.ncbi.nlm.nih.gov/geo/). The correlation of risk score (based on our signature) and lncRNAs were investigated. Statistical significance was determined using Spearman correlation coefficient |R| > 0.3 and p value< 0.05. The prediction of physical and functional interaction between selected lncRNA (CRNDE) and the proteins of our signature genes were performed in RNA-Protein Interaction Prediction (RPISeq, http://pridb.gdcb.iastate.edu/RPISeq/) using the protein sequence of signature genes and RNA sequence of the lncRNAs. The output, i.e., prediction probability of possible interactions was obtained in terms of RF and SVM classifiers. The interaction probabilities ranging from 0 to 1 were considered, being higher probabilities were better. In general, prediction probabilities with score more than 0.5 was considered “positive,” i.e., expressing the likelihood of interaction between given lncRNA and proteins. Next, the interaction of CRNDE (lncRNA) and mRNA of signature genes were explored. The prediction of physical and functional interaction between five lincRNAs and the mRNA of RGS20 was done using LncRRIsearch web server (http://rtools.cbrc.jp/LncRRIsearch/).

### Statistical analysis

Statistical analyses were performed using R Studio (version 2021.09.1; https://rstudio.com/). Kaplan-Meier analysis was performed using the R packages “survival” and “survminer”. Student’s t-test was used to compare differences between subgroups where the data were normally distributed, otherwise the Wilcox. Test was applied. Univariate and multivariate Cox proportional hazard regression analyses were performed with the R package “survival”, and LASSO analysis was performed with the R package “glmnet.” All statistical tests were two-sided and P<0.05 was considered statistically significant.

## Results

### Repetitive genome predominantly distributed in the proximity of ferroptosis genes

We first investigated the occurrence of repetitive genomic elements in the vicinity of ferroptosis genes (**Supplementary Figure 1**). For this purpose, we first determined the repetitive genome configuration in the promoter regions of these genes (~2KB). We found that Alu, MIR, L1 and L2 were the frequent repeats around 148 ferroptosis genes (**Supplementary Figure 1A**). Further clustering of these genes revealed high prevalence of LINE/L1, SINE/MIR, and SINE/Alu and low prevalence when these genes were combined or when LINE/L2 or others were included. In addition, the hierarchical distribution of repeats shows SINE/Alu and LINE/L1 as the divergent elements or with a significant discriminative score for gene clustering. To provide evidence for a possible functional relationship between gene clusters and repeats, we applied k-means clustering and functional enrichment analysis (**Supplementary Figure 1B**). Using this approach, we identified four significant gene clusters: L1-related (16 genes), Alu-related (59 genes), Alu/L1-related (17 genes), and unrelated genes (56 genes). In terms of their functional analysis, this discriminative analysis revealed the weight of each repeat type in the promoter region of gene sets that could define regulatory directions in the cellular and molecular context. We also checked the abilities of these clusters in other available datasets, such as the Allen Brain Atlas datasets, and found that each cluster represented a set of genes for some brain segments, e.g., “anterior cingulate area” for L1 and “paraventricular hypo-thalamic nucleus” for Alu-related repeats. For instance, the gene GABARAPL2 (highly associated to SINE/Alu) and the gene PARK7 (highly associated to LINE/L1) highlighted in our analysis, are known to be expressed highly in brain. In addition, these clusters were analyzed according to key cellular and molecular processes, e.g., the gene cluster more associated with LINE/L1 shows cellular responses to external stimuli (cytokine production, nongenomic effect of vitamin D, metabolic-related processes). Also, the gene cluster more associated with SINE/Alu shows standard metabolic processes and cellular adaptations to endogenous factors (response to starvation, autophagy, ferroptosis, fatty acid metabolism). Of interest, the cluster with Alu/L1-related genes reveals chromatin pathways mainly associated with the sirtulin 1 (SIRT1) and histone lysine methyltransferase (SUV39H1) genes, which could define specific configurations of these repeats for important regulatory processes.

### Ferroptosis-related gene signature predicts the survival in multiple myeloma

**Figure 1:**
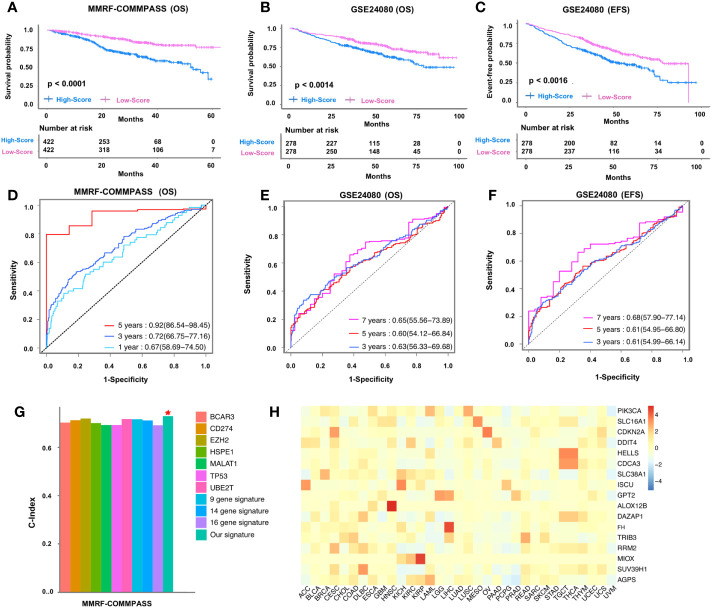
Clinical application of 14-gene signature in MMRF-COMMPASS and GSE24080 cohorts. Kaplan-Meier curves of the 14-gene signature risk score in the training cohort **(A)** and validation cohort **(B, C)**. ROC curves of the 14-gene signature risk score in the training **(D)** and validation **(E, F)** cohorts. Comparison of the C-indexes between the ferroptosis-related fourteen-gene signature and other existing biomarkers in multiple myeloma **(G)**. The risk coefficient between 14 genes and 33 types of cancer **(H)**. Red star represented our ferroptosis-related fourteen-gene signature.

We described our strategy in a flowchart (**Supplementary Figure 2**). Using the stringent strategy, the clinical characteristics of the patients involved in the study were outlined (**Supplementary Table 1**). We primarily used information from 844 patients (in the training cohort) and obtained 1669 survival-related genes (**Supplementary Table 2**). Thereafter, we intersected these survival-related genes with ferroptosis-related genes (n=269) and generated a list of 17 ferroptosis-related prognostic genes (**Supplementary Figure 3A**, **Supplementary Table 3**), which were further narrowed down based on risk coefficient scores (n=14) (**Supplementary Figures 3B, C**, **Supplementary Table 4**). Based on their mean risk score derived from the signature of 14 genes, the patients were classified into high-risk and low-risk groups. Notably, twelve signature genes (SLC38A1, CDKN2A, MIOX, AGPS, HELLS, FH, DAZAP1, SLC16A1, SUV39H1, DDIT4, TRIB3, ALOX12B) were found to be upregulated (**Supplementary Figures 4A–L**), while two (PIK3CA, ISCU) were downregulated in the high-risk group, based on the training cohort (**Supplementary Figures 4M, N**). It is important to mention that compared to the low-risk group, the high-risk group showed a worse outcome in the training cohort OS (P< 0.0001, **Figure 1A**), both in the validation cohort OS (P = 0.0014, **Figure 1B**), and in the validation cohort EFS (P = 0.00016, **Figure 1C**). To confirm the prognostic power and independence of the ferroptosis-related gene signature from other clinical characteristics (age, albumin, Β2M, creatine, CRP, hemoglobin, isotype, ISS stage, LDH, race, sex, risk), univariate and multivariate Cox analyses were also performed. Our analysis confirmed the robustness of the signature in the OS of the training cohort (univariate, HR = 1.1, 95% = 1.06-1.15, P<0.001; multivariate, HR = 1.06, 95%=1.02-1.1, P=0.005), OS of the validation cohort (univariate, HR = 1. 18, 95%=1.15-1.21, P<0.001; multivariate, HR=1.17, 95%=1.13-1.20, P<0.001) and EFS of the validation cohort (univariate, HR=1.08, 95%=1.05-1.12, P<0.001; multivariate, HR=1.05, 95%=1.02-1.09, P=0.004) (**Supplementary Table 5**). Thus, the gene signature associated with ferroptosis appears to be a reliable prognostic indicator for MM patients.

### Prognostic performance and clinical application of ferroptosis-related gene signature

Next, to assess the prognostic performance of the signature, we performed time-dependent (one-year, three-year, and five-year) dynamic AUC comparisons in the OS training cohort and obtain AUC values of 0.67, 0.72, and 0.92, respectively (**Figure 1D**). Of interest, the AUC value of the multigene risk score compared with ISS stage and sex fared better compared to the independent variables in the training cohort across each time point within 5 years (**Supplementary Figure 5A**). Likewise, in the GSE24080 training cohort, the AUC value of the multigene risk score was 0.63, 0.6, and 0.65 at 3, 5, and 7 years, respectively (**Figure 1E**), and each event within 7 years was found to be greater compared to the defined clinical variables (**Supplementary Figures 5B–D**). Similar results were obtained when the EFS of the validation cohort was used to assess the predictive power of this multigene risk score (AUC: 0.61, 0.61, 0.68 at 3, 5, and 7 years, respectively) (**Figure 1F**, **Supplementary Figures 5E–G**). To translate our obtained ferroptosis-related gene signature into clinical application, we integrated the training factors (patient age, sex, ISS stage, and multigene signature) into multivariate Cox analysis and constructed a nomogram to predict the survival probability of patients with MM (**Supplementary Figure 6A**). Analysis of the calibration curve, which included the nomogram after 3 and 5 years, showed a close resemblance to the diagonal curve at the same defined intervals (**Supplementary Figures 6B, C**). In addition, the C-index of the training cohort for overall survival was found to be 0.764 (95CI = 0.747-0.781), whereas the C-index of the validation cohort was estimated as 0.703 (95CI = 0.682-0.724), suggesting reliability of the nomogram. Of interest, we found superior performance and better prognostic ability of the obtained ferroptosis-related signature when compared with 10 already known biomarkers (32–41) (**Figure 1G**). This was also evident in the decision curve analysis (DCA) of the nomogram, where the threshold probability ranged from 14% to 95% and the probability of maximum net benefit exceeded 0.2 (**Supplementary Figure 7**). Besides, we investigated the reliability of the obtained signature in a panel of 29 cancers and found that these genes are relatively highly expressed in most cancers, especially in HNSC (squamous cell carcinoma of the head and neck), CESC (squamous cell carcinoma of the cervix and endocervical adenocarcinoma), and COAD (adenocarcinoma of the colon) (**Figure 1H**).

### Gene enrichment and immunofiltration analysis confirmed the relevance of the signature in high-risk MM patients

**Figure 2:**
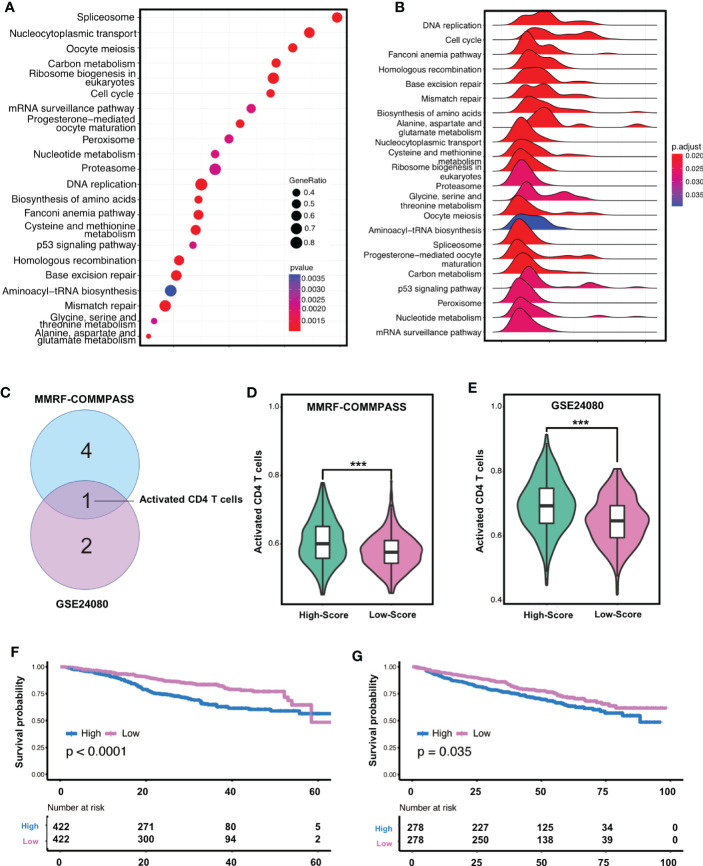
Gene enrichment and immunofiltration analysis confirmed the relevance of the signature in high-risk MM patients. **(A)** Bubble diagram shows gene counts and gene ratio of the significantly enriched KEGG pathway terms. **(B)** Ridgeline plot shows enrichment score of the significantly enriched KEGG pathway terms. **(C)** Immune cell population alternation associated with OS with statistically significant difference in both cohorts. **(D, E)** the enrichment degree of high-risk group was significantly higher than that of low-risk group in both cohorts by ssGSEA analyses *** indicates P-value < 0.001. **(F, G)** Activated CD4+T cells were relevant to survival in both the training cohorts. KEGG, kyoto encyclopedia of genes and genomes; GSEA, gene set enrichment analysis; ES, enrichment score.

The result of CIBERSORT indicated that the plasma cells accounted for more than 85% (**Supplementary Figure 8A**), which was consistent with the experimental protocol of the GSE24080 or MMRF-COMMPASS study. To our surprise, non-plasma cells together, including memory B cells, CD4+ T cells, and activated NK cells (**Supplementary Figure 8B**), could account for more than 10% of the tumor microenvironment. These immune cells should not be ignored despite low absolute content (42). Considering the negative correlation between the risk score of ferroptosis-related gene signature and the clinical outcome of MM, KEGG enrichment analysis was performed between the high-risk and low-risk groups. We found that 22 KEGG terms were significantly enriched in the high-risk group (**Figure 2A**), with DNA replication being the highly enriched (Enrichment score = 0.8046), while the mRNA surveillance pathway had the lowest enrichment (Enrichment score = 0.5593) (**Figure 2B**). Notably, four ferroptosis-related pathways, including proteasome (NES = 1.6748, adjusted p-value = 0.0263, 5q-value = 0.0181), cysteine and methionine metabolism (NES = 1.7277, adjusted p-value = 0.0195, q-value = 0.0134), p53 signaling pathway (NES = 1.5643, adjusted p-value = 0.0263, q-value = 0.0181) and DNA replication (NES = 1.9783, adjusted p-value = 0.0195, q-value = 0.0134), were found to be significantly enriched in high-risk MM patients (**Supplementary Figure 9**).

Since immune cell infiltration may have a differential impact on high and low-score patients, we next assessed the degree of immune infiltration using the ESTIMATE algorithm. The analysis showed that the high-risk MM in the training cohort had lower immune and stromal score but high tumor purity (**Supplementary Figures 10A–C**), which was also confirmed in the validation cohort (**Supplementary Figures 9D–F**). Of interest, we found that immune and stromal score were negatively correlated with risk score, whereas tumor purity was positively correlated with risk score in the training cohort (**Supplementary Figures 10G–I**). To further investigate the influence of immune cell population alternation, we built cox proportional hazards regression models based on the enrichment level of 28 immune infiltration-related gene sets *via* ssGSEA analyses, and focused on whether the alternation of these gene sets was related with poor outcome. In the training cohort, activated CD4+T cells, regulatory T cells, and type 1 T helper cells were significantly negatively associated with OS (P<0.05) (**Supplementary Figure 11A**), whereas follicular T helper cells and immature B cells were significantly positively associated with OS (P<0.05). Meanwhile, in the validation cohort (**Supplementary Figure 11B**), activated CD4+T cells and type 2 T helper cells were significantly negatively associated with OS (P<0.05), whereas type 17 T helper cells were significantly positively associated with OS (P<0.05). Thus, we noticed that activated CD4+T cells was significantly negatively associated with OS in both cohorts (**Figure 2C**). Interestingly, the enrichment degree of high-risk group was significantly higher than that of low-risk group in both cohorts (**Figures 2D, E**). Of significance, only activated CD4+T cells were relevant to survival in both the training cohort (p<0.001) and the validation cohort (p=0.035) (**Figures 2F, G**).

### Oncogenic lncRNA CRNDE and signature genes display strong correlation

**Figure 3:**
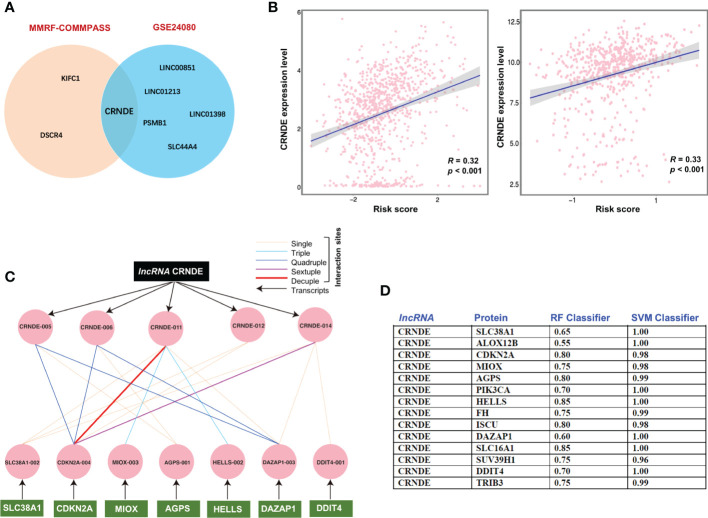
Oncogenic lncRNA CRNDE and signature genes display strong correlation. **(A)** The potential significant correlation of obtained ferroptosis gene signature with the lncRNAs. **(B)** lncRNA CRNDE was significantly correlated in both cohorts, MMRF-COMMPASS cohort (left) and GEO cohort (right). **(C)** The interaction between the transcript of lncRNA CRNDE and signature genes related mRNA. **(D)** The interaction between lncRNA CRNDE and signature genes related proteins.

Since an increasing number of studies have indicated that non-coding RNAs may modulate the process of ferroptotic cell death (43–45). Herein, we also assessed the potential correlation of obtained ferroptosis gene signature with the lncRNAs. Of interest, while we found only a few lncRNAs in the training cohort (KIFC1, DSCR4) and in the validation cohort (SLC44A4, PSMB1, LINC01398, LINC01213, LINC00851), while the oncogenic lncRNA CRNDE was significantly correlated in both cohorts (**Figure 3A**). We also observed that the risk score increased significantly with increasing expression of CRNDE in both cohorts, MMRF-COMMPASS cohort (left) and GEO cohort (right). (**Figure 3B**). Further analysis revealed that the lncRNA CRNDE and signature genes interact with each other at both the mRNA level and the protein level (**Figures 3C, D**). Our analysis showed that all tested proteins were reasonably good interaction partners of the lncRNA CRNDE. However, a selected few proteins such as HELLS, SLC16A1, and CDKN2A were predicted to be the major interacting partners of the lncRNA CRNDE. With one exception (DDIT4), all other ferroptosis associated genes in the obtained signature were enriched in repetitive genomes, especially with Alu-related repeats (MIOX, HELLS, DAZAP1, TRIB3, PIK3CA, SUV39H1).

## Discussion

Emerging evidence suggests that ferroptosis may be the target of innovative antitumor therapies (46, 47). Given this, there have been a multitude of studies that have defined several aspects of genes and mechanisms related to ferroptosis in cancers (48–50). However, the effects of genome organization (repetitive genome) in the proximity of these genes have not been investigated. In addition, only a few studies have investigated the aspect of ferroptosis in multiple myeloma (MM), a form of cancer characterized by excessive proliferation and dysfunction of certain plasma cells in the bone marrow (5). MM being a hematological malignancy harbors biological complexity due to several disrupted cancer pathways resulting from multiple genetic abnormalities and epigenetic aberrations (4, 51, 52). A recent study showed that induction of Ferroptosis in MM cells triggers DNA methylation and histone modification changes associated with cellular senescence (4). Similarly, a study compared the kinomic activity profile of the natural anticancer agent withaferin A with apoptotic and ferroptotic signatures to predict the mode of cell death in MM cells (53). Considering this, herein, we investigated the genomic architecture of ferroptosis-related genes and independently constructed a prognostic signature for patient stratification in MM.

To this end, we first defined the ferroptosis-related genes and assessed the presence of the repetitive genome in their promoter region and specified the gene clusters with L1, Alu, and L1/Alu repeats. To mention, such repeats have been described by their regulatory role in gene expression (54)., however, the significance of their distribution or configuration remains unclear. Moreover, further clustering of these genes revealed high prevalence of LINE/L1, SINE/MIR, and SINE/Alu and low prevalence when these genes were combined or when LINE/L2 or others were included. The functional analysis revealed that the obtained clusters are involved in key cellular and molecular processes. For instance, the gene cluster associated with LINE/L1 showed involvement in cellular responses to external stimuli (cytokine production, nongenomic effect of vitamin D, metabolic-related processes). While, the gene clusters more associated with SINE/Alu showed standard metabolic processes and cellular adaptations to endogenous factors (response to starvation, autophagy, ferroptosis, fatty acid metabolism). Of interest, the clusters with Alu/L1-related genes show chromatin pathways primarily linked sirtulin 1 (SIRT1) and histone lysine methyltransferase (SUV39H1) genes.

Next, using the stringent strategy, we established the ferroptosis-related prognostic genes signature containing fourteen genes (SLC38A1, CDKN2A, MIOX, AGPS, HELLS, FH, DAZAP1, SLC16A1, SUV39H1, DDIT4, TRIB3, ALOX12B, PIK3CA, ISCU). We further classified patients into high-risk and low-risk groups based on the mean risk score according to signature. We also confirmed the prognostic power and independence of the obtained signature related to ferroptosis with other clinical characteristics (age, albumin, Β2M, creatinine, CRP, hemoglobin, isotype, ISS stage, LDH, race, sex, risk) and found it to be a reliable indicator for patients with MM. Besides, we investigated the reliability of the obtained signature in a panel of 29 cancers and found that these genes are relatively highly expressed in most cancers. Importantly, we found superior performance and better prognostic ability of the obtained ferroptosis-gene signature compared to 10 already known biomarkers. The outcome of decision curve analysis (DCA) of the nomogram affirmed the possible use of this signature for clinical utility. As next, we utilized the ferroptosis-related gene signature for GSEA analysis and found that several KEGG terms significantly enriched in the high-risk group. Among them, four ferroptosis-related pathways, including proteasome, cysteine and methionine metabolism, p53 signaling pathway and DNA replication, were found to be significantly enriched in high-risk MM patients. In terms of clinical application, myeloma patients receiving proteasome inhibitor (ie, bortezomib) benefited in OS compared to those who did not receive proteasome inhibitor (55)., and proteasome inhibitor was commonly used to treat relapsed/refractory myeloma, either as single agent or combined with other therapies (56). Given that immune cell infiltration may have a differential impact on high and low-score patients, we also assessed the degree of immune infiltration and found that the high-risk MM (in the training cohort) had lower immune and stromal score but high tumor purity. Among several immune cell populations, we found that activated CD4+ T cells and activated CD8+ T cells were significantly upregulated in the high-score group. Of significance, only activated CD4+ T cells were relevant to survival in both the training cohort and the validation cohort. Since MM is an immunoproliferative disease, the increased frequency of Tregs and T cells possessing a regulatory function have already been discussed MM patients (57). An independent study also reported a higher proportion of activated CD4+ Tregs in MM patients compared to healthy donors (58). The robust signature we used to establish the relationship between activated CD4+ T cell subsets and patient survival is concordant to these studies. However, the exact mechanism by which immune cells, especially activated CD4+ T cell subsets, affect MM remains unclear. To mention, some of the genes in our signature has already been implicated in MM, for instance, ALOX12B variants has been proposed as a biomarker for progression and resistance in MM (59). CDKN2A has previously been found to be differentially expressed in MM (60)., and its overexpression has been correlated with poor OS in MM (61). SUV39H1 and the contribution of other epigenetic modifiers has been implicated in MM development and disease progression (62). To mention, some studies have been conducted on prognostic gene signatures related to cell death mechanisms such as ferroptosis (63, 64) and autophagy (65, 66) seeking their potential role in cancer treatment. Of importance, the ferroptosis-related gene signature, we presented in the current study is the first for MM.

Next, we investigated whether the obtained signature shows any potential correlation with the lncRNAs implicated in cancers. Interestingly, we identified few lncRNAs (KIFC1, DSCR4) in the training cohort and (SLC44A4, PSMB1, LINC01398, LINC01213, LINC00851) in the validation cohort appears to correlate with the signature. Most importantly, we identified the oncogenic lncRNA CRNDE significantly correlated in both training and validation cohorts. LncRNA CRNDE has been found to be altered in several cancers, including colorectal cancer, glioma, hepatocellular carcinoma, lung cancer, breast cancer, gastric cancer, and renal cell carcinoma (67, 68). Of interest, a recent study performed CRISPR-mediated deletion of the lncRNA CRNDE and showed decrease in IL6 signaling and proliferation responses in multiple myeloma cells (69). Our analysis revealed that lncRNA CRNDE and signature genes interact with each other at both the mRNA level and the protein level. Hence, by using multiple myeloma, we support the potential use of non-coding genome based ferroptosis targeting, which has recently been suggested (23).

It is also important to mention the limitations of this study, such as: 1) we did not evaluate the impact of therapies (e.g., targeted therapy and/or chemotherapy, with or without steroids, etc.) on the defined high/low risk groups of MM patients. 2) Mutations in certain genes (including KRAS, NRAS, TP53, FAM46C, DIS3 and BRAF) have a high recurrence rate in MM, however, we did not calibrate our signature according to the mutation spectrum of patients. 3) The experimental validation of our signature is a requisite. 4) The current methodologies for the enrichment of plasma cells specially by using anti-CD138 immunomagnetic bead selection may lead to some potential bias for assessing the immune microenvironment components (e.g. memory B cells, CD4+ T cells, and activated NK cells), the composition of non-plasma cells may proportionally be lower/affected. Despite this, our study is the first to define the effects of the repetitive genome on the proximity of ferroptosis related genes and their putative association with the oncogenic lncRNA CRNDE.

## Conclusions

We showed that ferroptosis-related genes are enriched with the repetitive genome in their proximity, with a strong predominance of the SINE family, followed by LINE, of which the most significant discriminant values were SINE/Alu and LINE/L1, respectively. In addition, we developed an independent predictive model/signature comprising fourteen ferroptosis-related genes that can identify MM high-risk patients with lower immune/stromal score and higher tumor purity in their immune microenvironment.

Besides, we found that the oncogenic lncRNA CRNDE correlated with the risk score and was highly associated with most of the signature genes.

## Data availability statement

The datasets presented in this study can be found in online repositories. The names of the repository/repositories and accession number(s) can be found in the article/**Supplementary Material**.

## Author contributions

Conceptualization, JQ, AS, YW, IS-W, and CJ. Data collection, JQ, AS, YW, FT-T,TD, HDL, HJL, BK, CK, TL, and CZ. Data curation, JQ, AS, YW, FT-T,TD, HDL, HJL, BK, CK, CZ, IS-W, and CJ. All authors contributed to the article and approved the submitted version.

## Supplementary material

Supplementary Figure 1 Association of repetitive genome content in the upstream promoter region and their functional meaning for ferroptosis-related genes. **(A)** Hierarchical clustering of genes based on the normalized score of repeat quantity and length “Log (Repeat content)”. **(B)** On top: k-means clustering of genes based on SINE/Alu and LINE/L1 (brain sections are from Allen Brain Atlas up-expression enrichment) and bottom: functional enrichment of each gene cluster.

Supplementary Figure 2 Flowchart of the study. LASSO, the least absolute shirankage and selection operator Cox regression model; ROC, receiver operating characteristic; MM, multiple myeloma.

Supplementary Figure 3 Construction of the prognotic gene signature using LASSO regression analysis. **(A)** Venn Diagram represents 17 potential prognostic genes composed from Intersecting 1669 univariate genes with 269 ferroptosis genes. **(B)** LASSO coefficient profiles of 17 ferroptosis-related potential prognostic genes. Each curve corresponds to a gene. **(C)** Selection of the optimal parameter in LASSO regression with 10-fold cross validation. LASSO, the least absolute shirankage and selection operator Cox regression model.

Supplementary Figure 4 The distribution of fourteen signature genes based on their mean risk score. **(A–L)** SLC38A1, CDKN2A, MIOX, AGPS, HELLS, FH, DAZAP1, SLC16A1, SUV39H1, DDIT4, TRIB3, ALOX12B. (M-N) PIK3CA, ISCU.

Supplementary Figure 5 Time-dependent dynamic AUC curves of the 14-gene signature risk score in the training **(A)** and validation **(B–G)** cohorts. The time-dependent dynamic AUC curve shows a comparison between the risk score and other independent factors. AUC, area under the ROC curve; ROC, receiver operating characteristic.

Supplementary Figure 6 Nomogram and its associated calibration curve analysis. **(A)** Ferroptosis-related fourteen-gene based nomogram predicting the 3- and 5-year survival probability in patients with multiple myeloma. **(B, C)** Calibration analysis of ferroptosis-related fourteen-gene containing nomogram at 3 years **(B)** and 5 years **(C)**.

Supplementary Figure 7 Decision curve analysis of the clinical use of ISS stage and the ferroptosis-related fourteen-gene based nomogram in multiple myeloma.

Supplementary Figure 8 Cell composition of tumor microenvironment investigated by CIBERSORT. **(A)** Cell composition of Bone marrow **(B)** Non-plasma cell composition.

Supplementary Figure 9 GSEA result of KEGG gene set based on the risk-score of each multiple myeloma patients. **(A)** Proteasome **(B)** Cysteine and methionine metabolism **(C)** p53 signaling pathway **(D)** DNA replication **(E)** Homologous recombination **(F)** Base excision repair **(G)** Mismatch repair **(H)** Fanconi anemia pathway **(I)** Spliceosome **(J)** Aminoacyl-tRNA biosynthesis **(K)** Cell cycle **(L)** Nucleotide metabolism. KEGG, kyoto encyclopedia of genes and genomes; GSEA, gene set enrichment analysis; NES, normalizedN enrichment score; FDR, false discovery rate.

Supplementary Figure 10 Relationship between immune infiltration level and ferroptosis-related fourteen-gene risk score. The distribution of immune score, stromal score and tumor purity upon different risk score in the training cohort **(A–C)** and validation cohort **(D–F)**. The correlation between risk score and the distribution of immune score **(G)**, stromal score **(H)** and tumor purity **(I)**, respectively. *p< 0.05, **p< 0.01, ***p< 0.001.

Supplementary Figure 11 Cox proportional hazards regression models based on the enrichment level of 28 immune infiltration-related gene sets *via* ssGSEA analyses in **(A)** MMRF-COMMPASS study and **(B)** GSE24080. Red stars indicate P<0.05.
