# Supplementary figures and images for: Systematic discrimination of the repetitive genome in proximity of ferroptosis genes and a novel prognostic signature correlating with the oncogenic lncRNA CRNDE in multiple myeloma
Source: Front Oncol. 2022 Dec 20;12:1026153. doi: 10.3389/fonc.2022.1026153 (PMC9808058; doi:10.3389/fonc.2022.1026153)

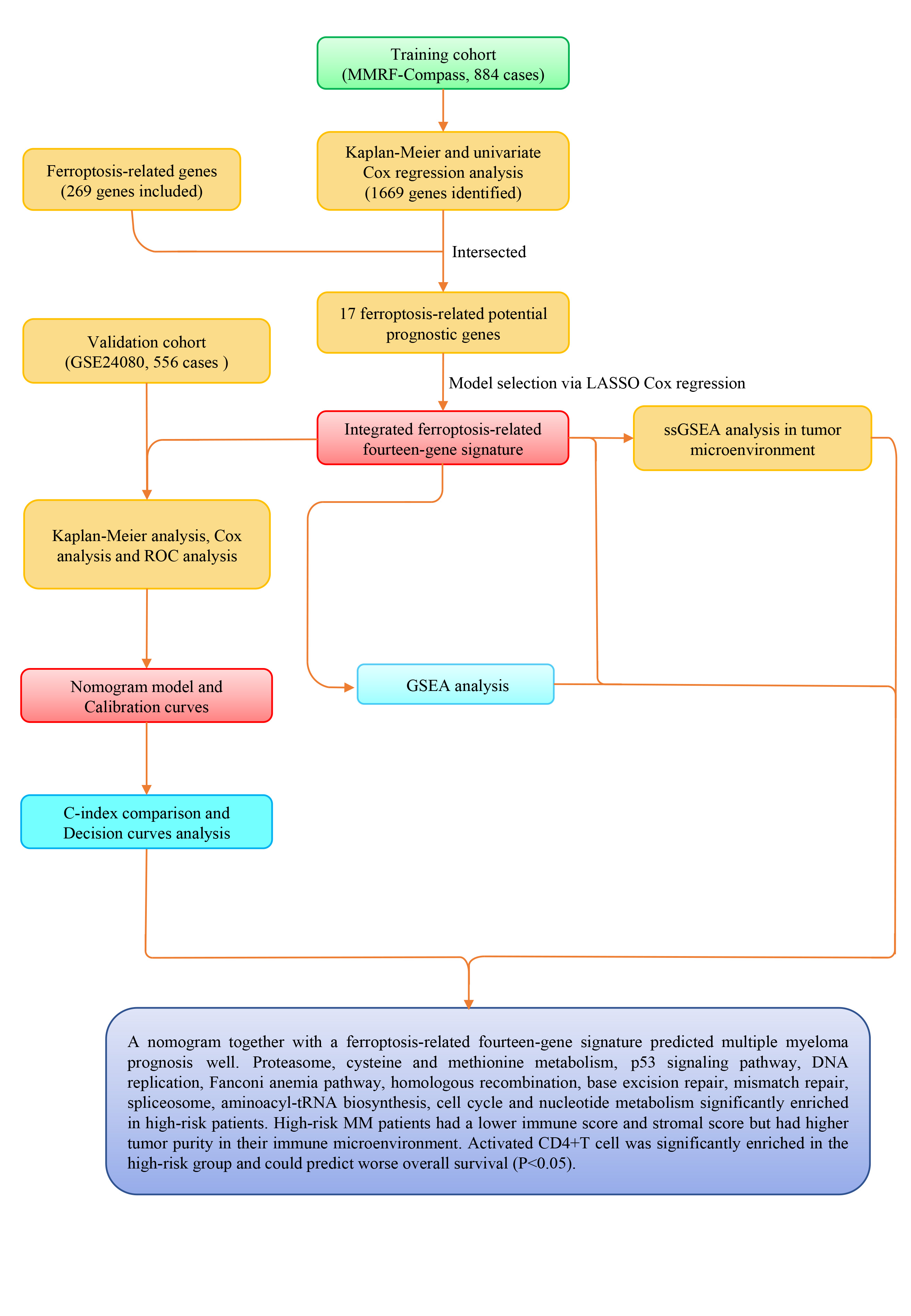

Supplement: Supplementary Figure 2 — Flowchart of the study. LASSO, the least absolute shirankage and selection operator Cox regression model; ROC, receiver operating characteristic; MM, multiple myeloma. [file Image_2.jpeg]

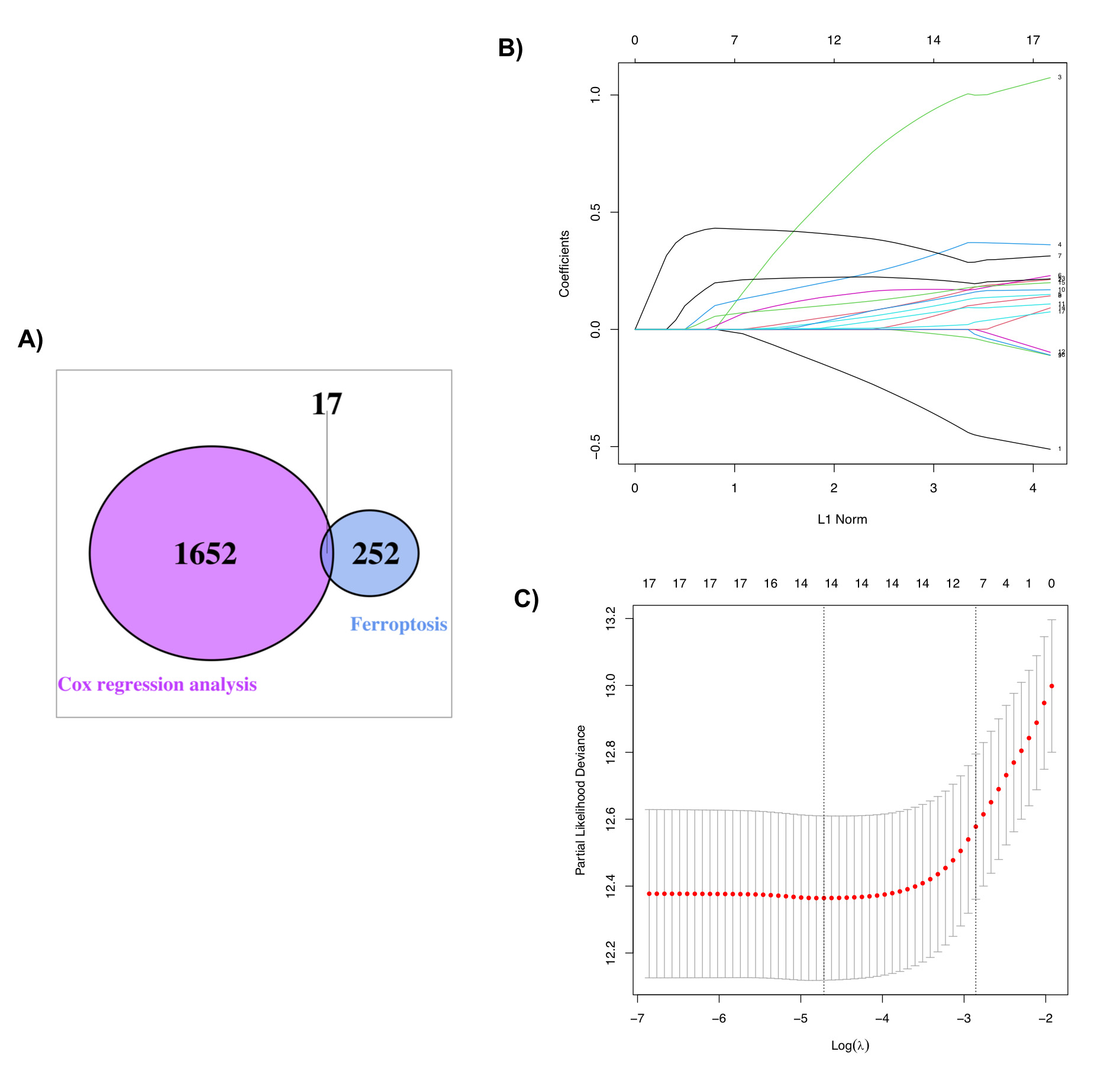

Supplement: Supplementary Figure 3 — Construction of the prognotic gene signature using LASSO regression analysis. (A) Venn Diagram represents 17 potential prognostic genes composed from Intersecting 1669 univariate genes with 269 ferroptosis genes. (B) LASSO coefficient profiles of 17 ferroptosis-related potential prognostic genes. Each curve corresponds to a gene. (C) Selection of the optimal parameter in LASSO regression with 10-fold cross validation. LASSO, the least absolute shirankage and selection operator Cox regression model. [file Image_3.jpeg]

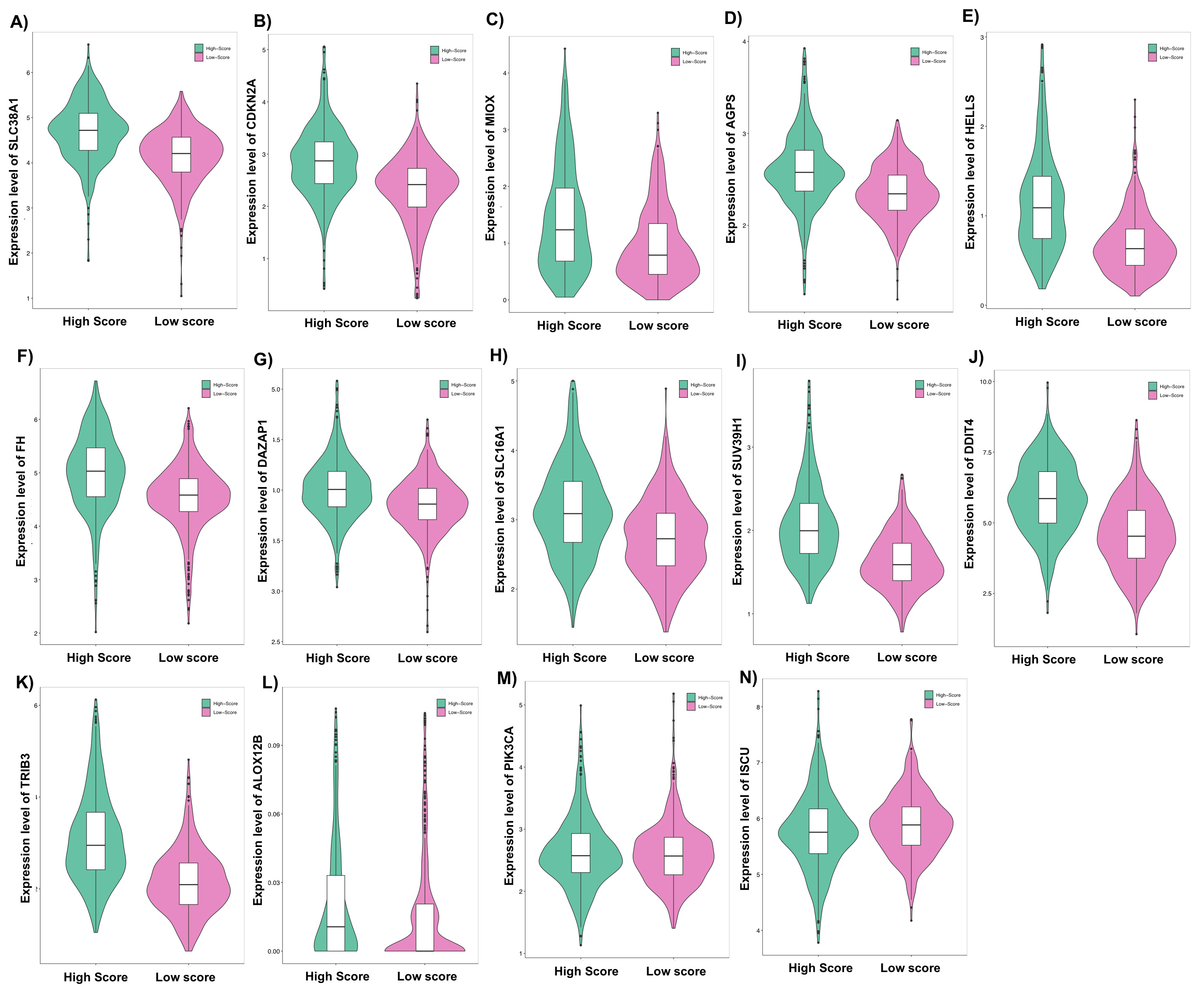

Supplement: Supplementary Figure 4 — The distribution of fourteen signature genes based on their mean risk score. (A–L) SLC38A1, CDKN2A, MIOX, AGPS, HELLS, FH, DAZAP1, SLC16A1, SUV39H1, DDIT4, TRIB3, ALOX12B. (M-N) PIK3CA, ISCU. [file Image_4.jpeg]

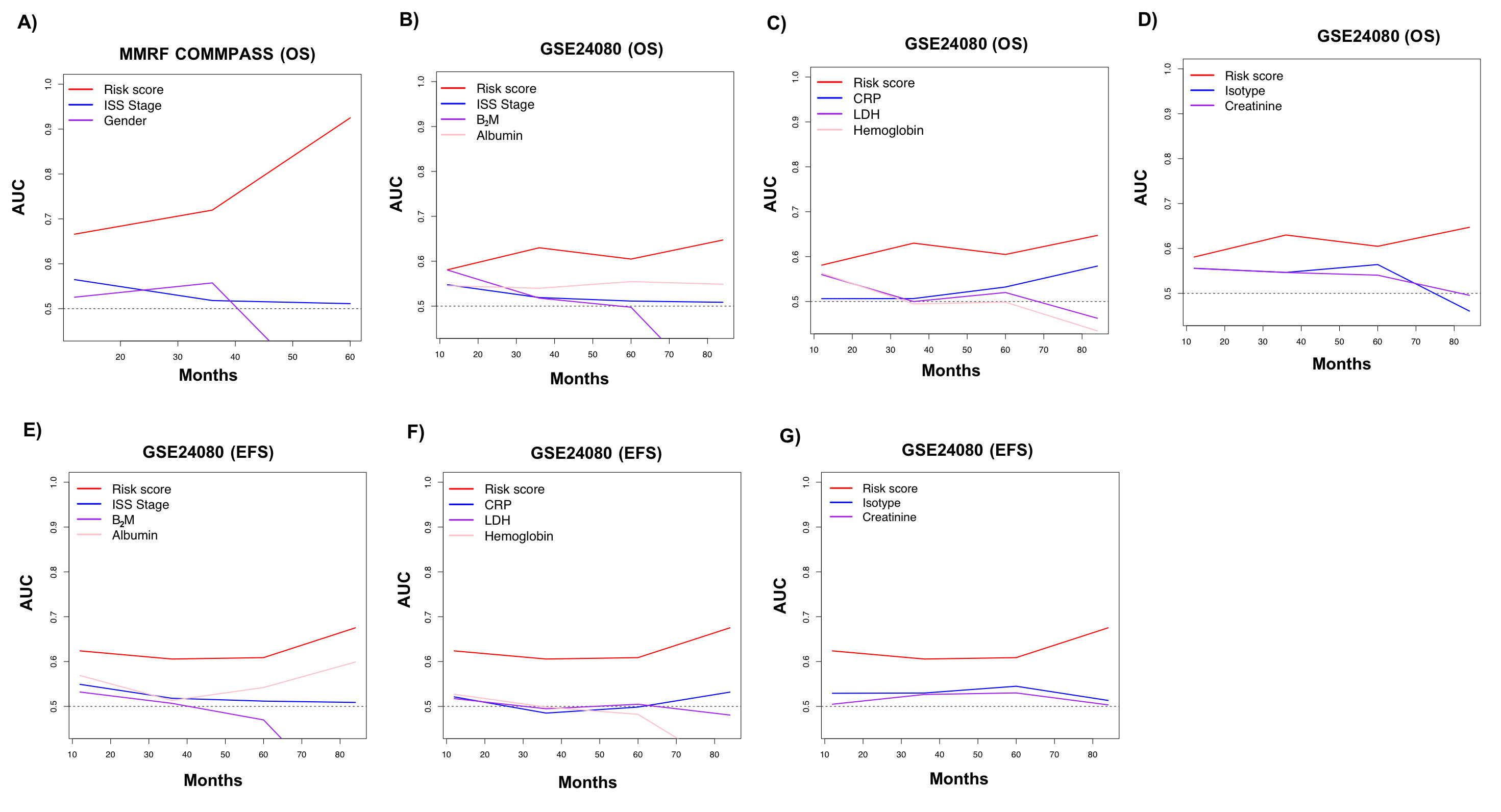

Supplement: Supplementary Figure 5 — Time-dependent dynamic AUC curves of the 14-gene signature risk score in the training (A) and validation (B–G) cohorts. The time-dependent dynamic AUC curve shows a comparison between the risk score and other independent factors. AUC, area under the ROC curve; ROC, receiver operating characteristic. [file Image_5.jpeg]

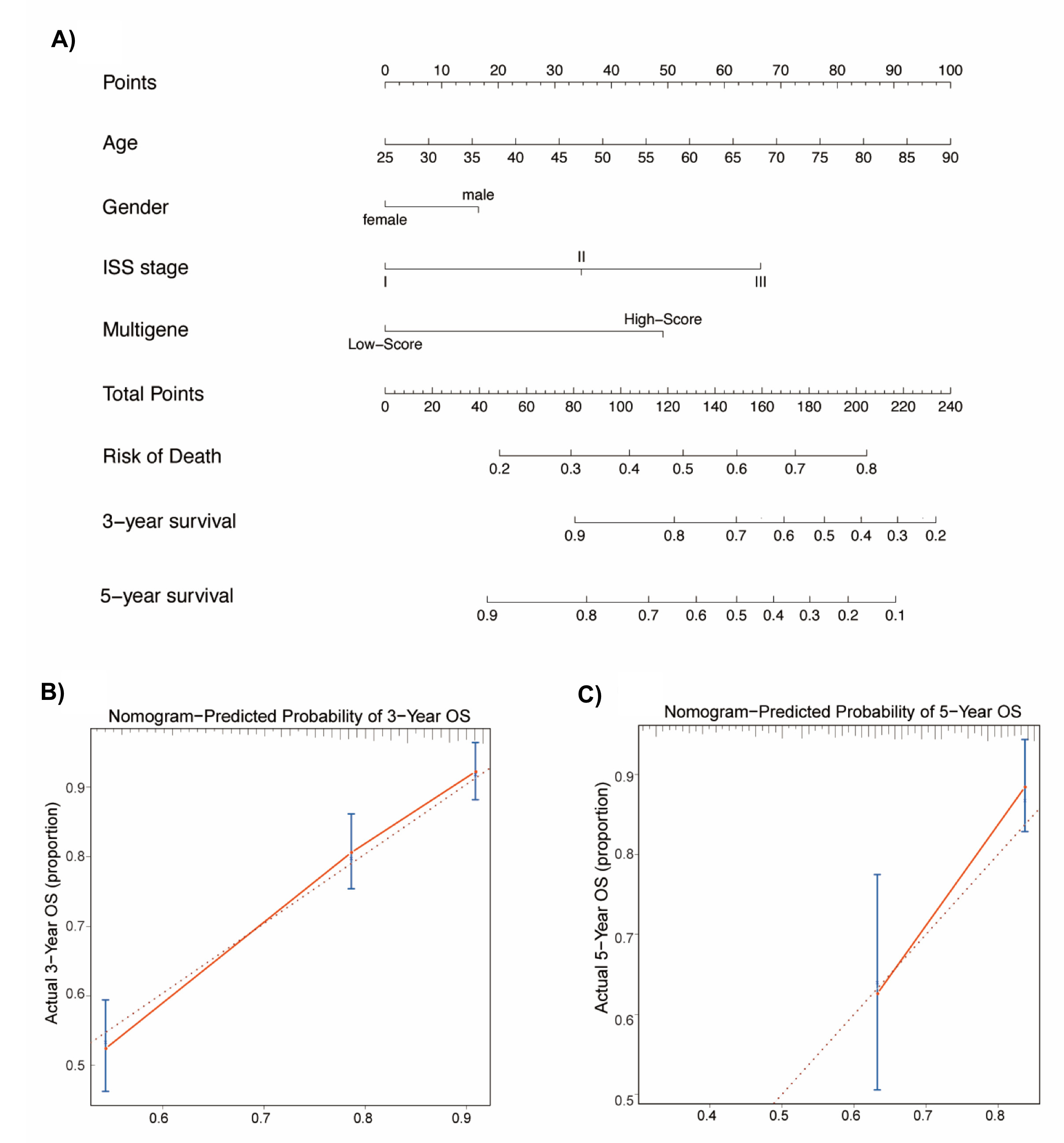

Supplement: Supplementary Figure 6 — Nomogram and its associated calibration curve analysis. (A) Ferroptosis-related fourteen-gene based nomogram predicting the 3- and 5-year survival probability in patients with multiple myeloma. (B, C) Calibration analysis of ferroptosis-related fourteen-gene containing nomogram at 3 years (B) and 5 years (C). [file Image_6.jpeg]

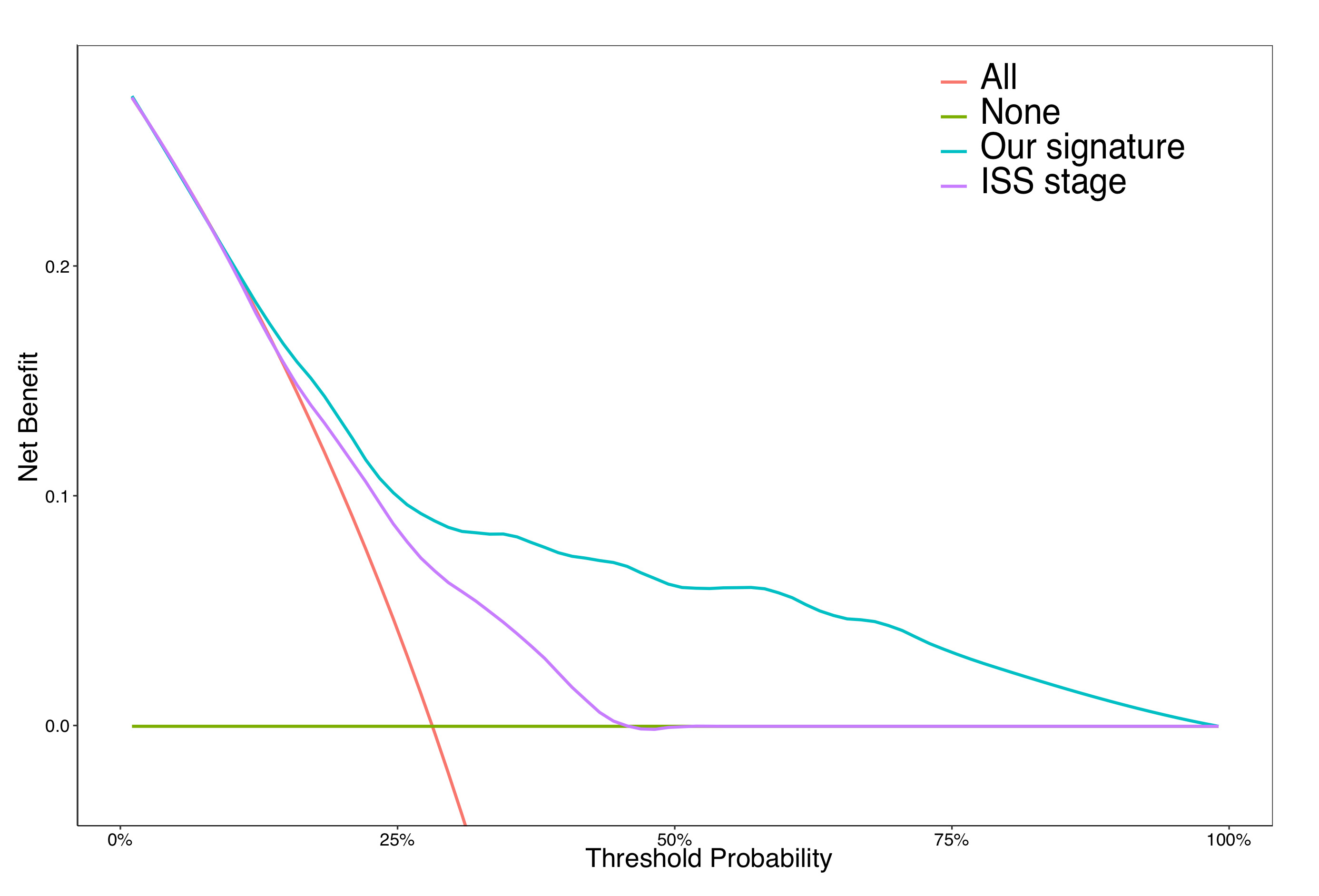

Supplement: Supplementary Figure 7 — Decision curve analysis of the clinical use of ISS stage and the ferroptosis-related fourteen-gene based nomogram in multiple myeloma. [file Image_7.jpeg]

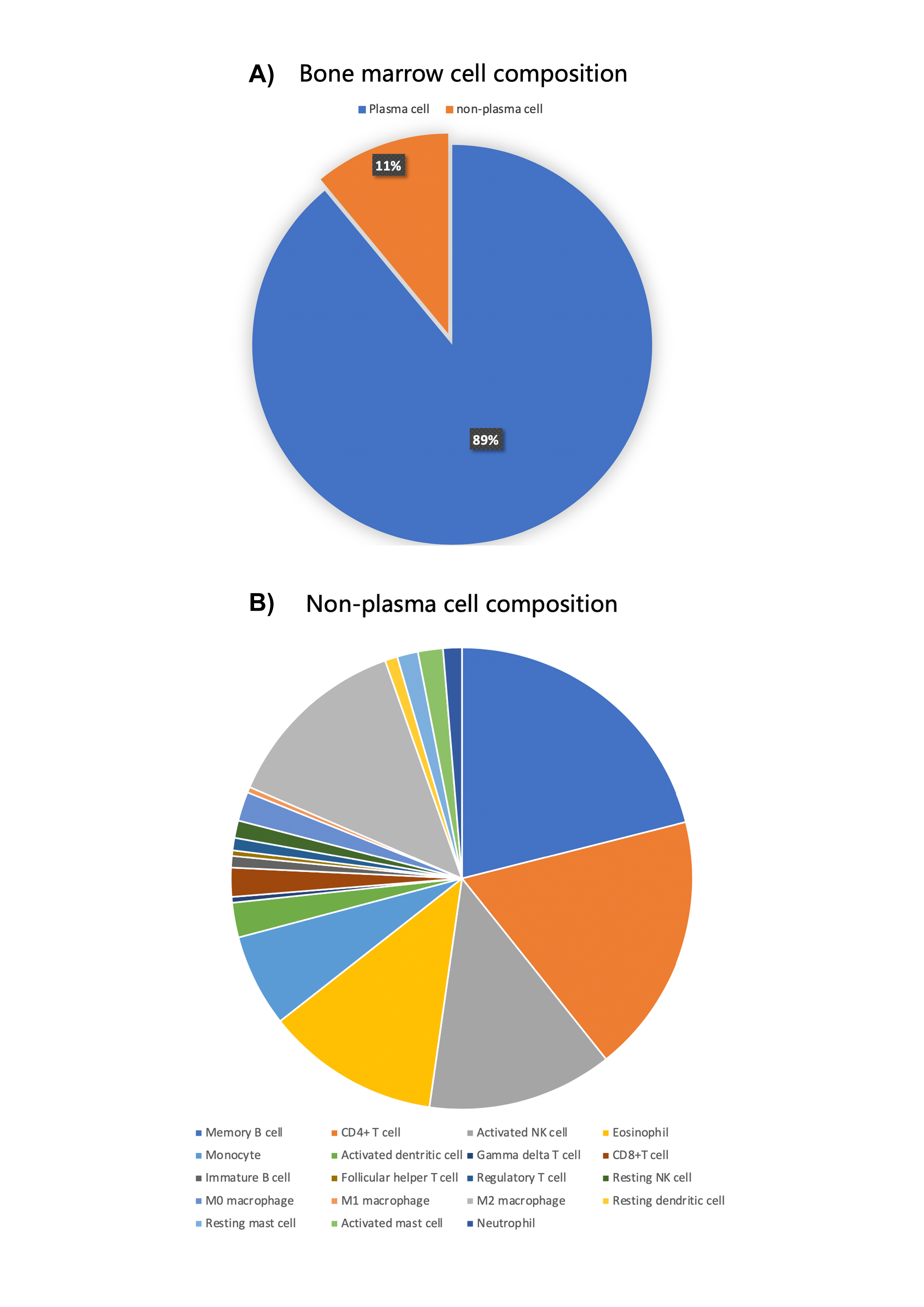

Supplement: Supplementary Figure 8 — Cell composition of tumor microenvironment investigated by CIBERSORT. (A) Cell composition of Bone marrow (B) Non-plasma cell composition. [file Image_8.tif]

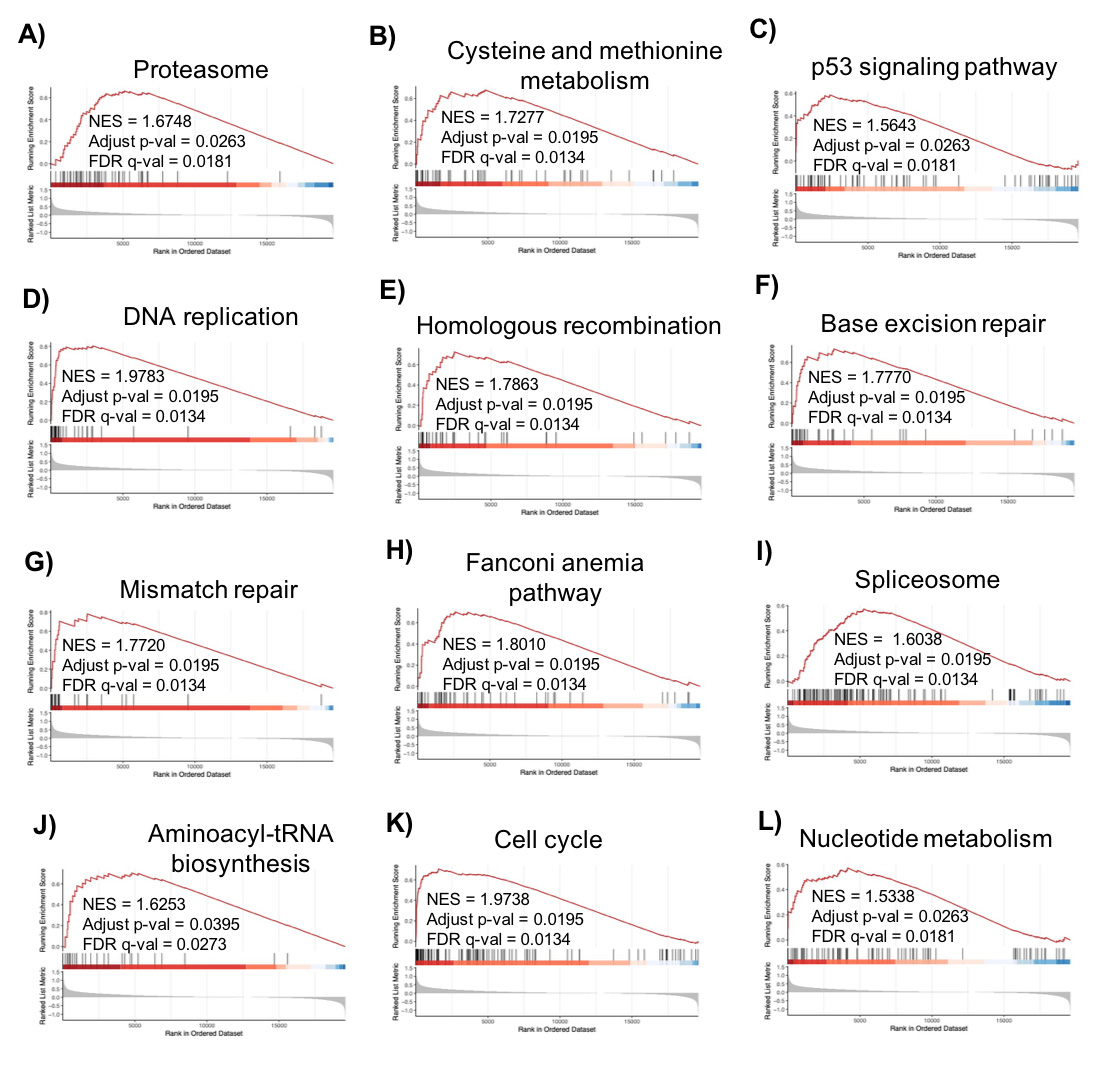

Supplement: Supplementary Figure 9 — GSEA result of KEGG gene set based on the risk-score of each multiple myeloma patients. (A) Proteasome (B) Cysteine and methionine metabolism (C) p53 signaling pathway (D) DNA replication (E) Homologous recombination (F) Base excision repair (G) Mismatch repair (H) Fanconi anemia pathway (I) Spliceosome (J) Aminoacyl-tRNA biosynthesis (K) Cell cycle (L) Nucleotide metabolism. KEGG, kyoto encyclopedia of genes and genomes; GSEA, gene set enrichment analysis; NES, normalizedN enrichment score; FDR, false discovery rate. [file Image_9.jpeg]

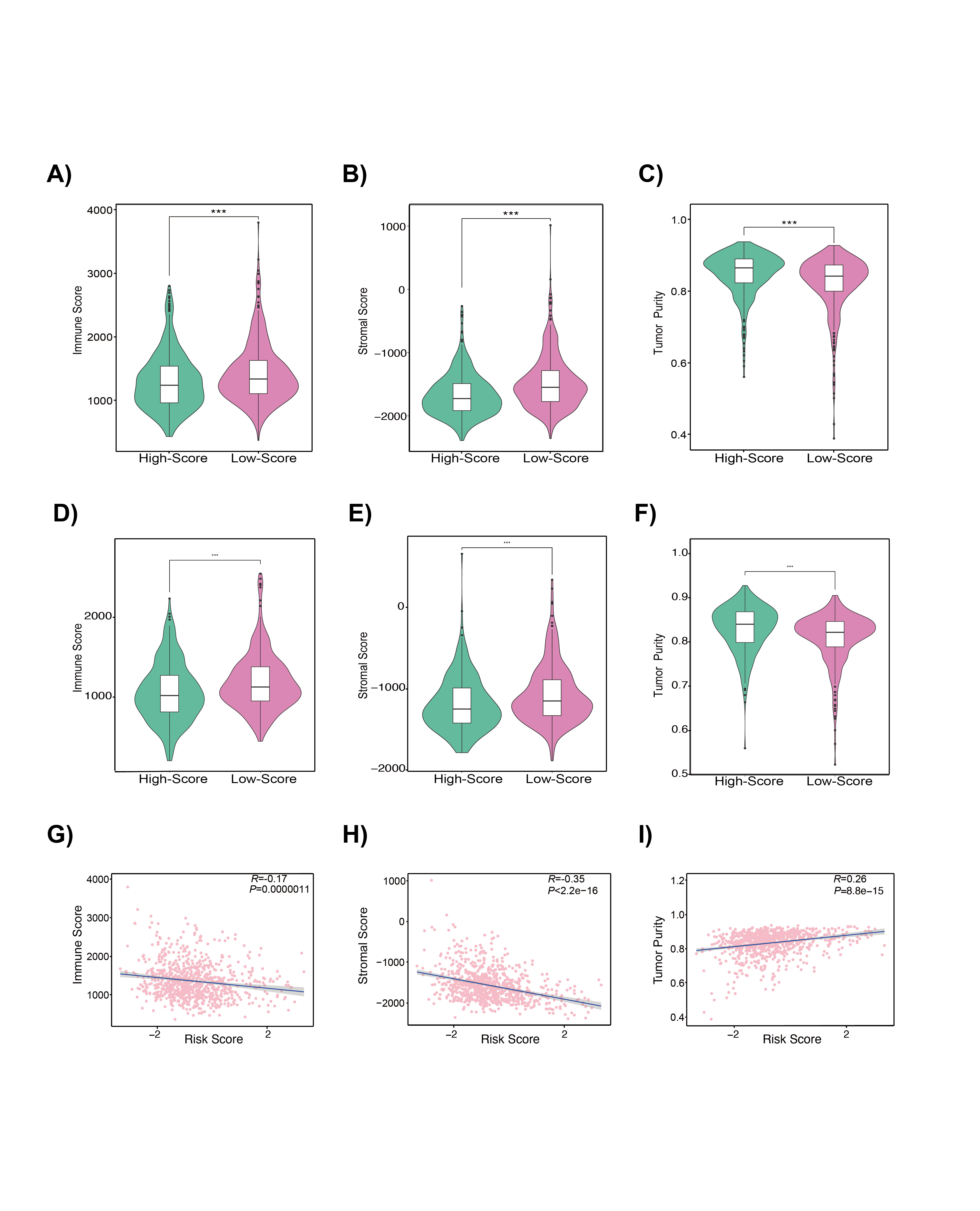

Supplement: Supplementary Figure 10 — Relationship between immune infiltration level and ferroptosis-related fourteen-gene risk score. The distribution of immune score, stromal score and tumor purity upon different risk score in the training cohort (A–C) and validation cohort (D–F). The correlation between risk score and the distribution of immune score (G), stromal score (H) and tumor purity (I), respectively. *p< 0.05, **p< 0.01, ***p< 0.001. [file Image_10.jpeg]

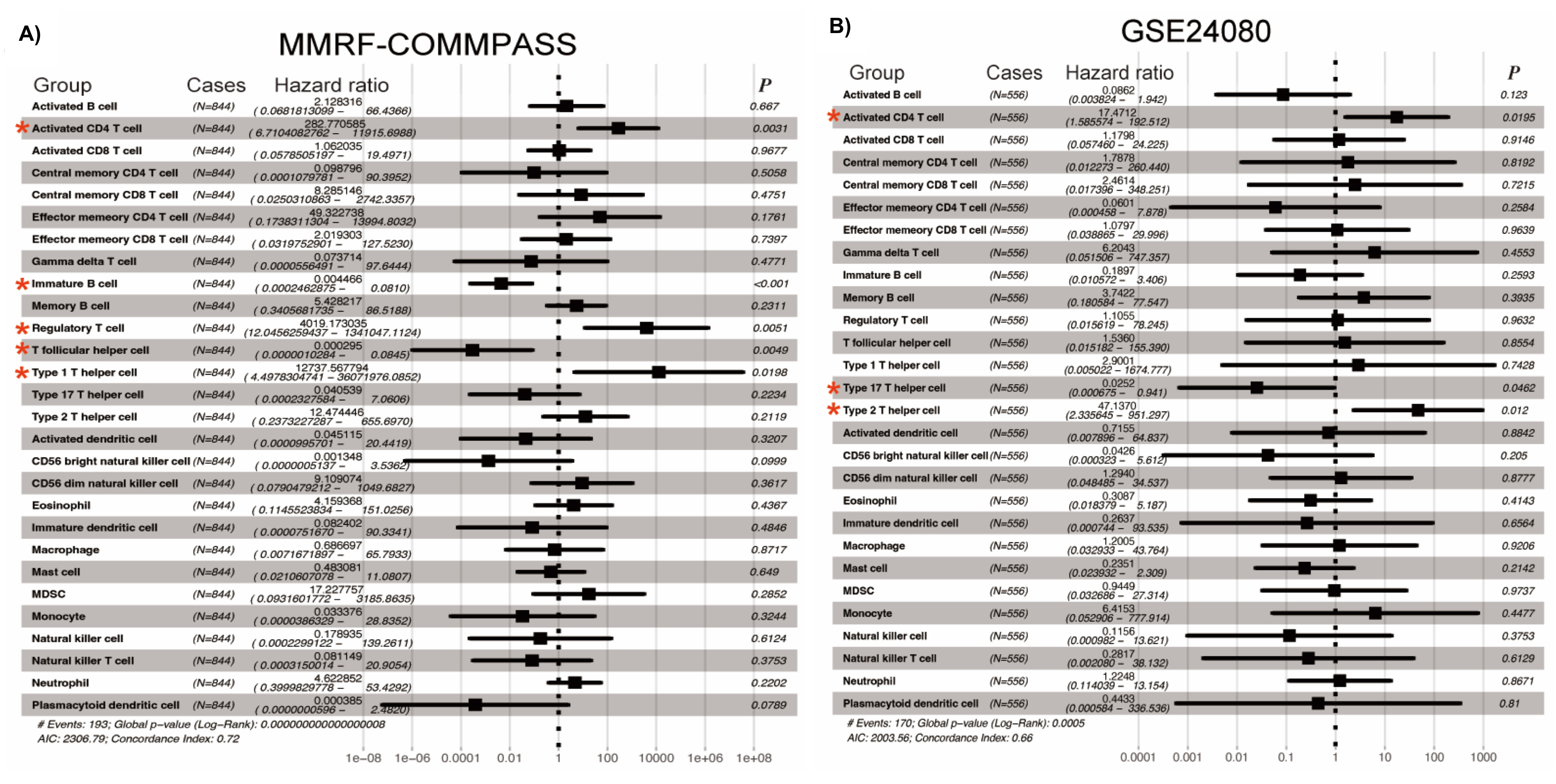

Supplement: Supplementary Figure 11 — Cox proportional hazards regression models based on the enrichment level of 28 immune infiltration-related gene sets via ssGSEA analyses in (A) MMRF-COMMPASS study and (B) GSE24080. Red stars indicate P<0.05. [file Image_11.jpeg]
