# Supplementary material for: Systematic discrimination of the repetitive genome in proximity of ferroptosis genes and a novel prognostic signature correlating with the oncogenic lncRNA CRNDE in multiple myeloma
Source: Front Oncol. 2022 Dec 20;12:1026153. doi: 10.3389/fonc.2022.1026153 (PMC9808058; doi:10.3389/fonc.2022.1026153)
Supplement: Supplementary file 12 [file Table_1.docx]

**Supplementary table 1. Clinical characteristics of patients involved in the study.**

| Characteristic | Training cohort  (MMRF-COMMPASS, 844 cases) | Validation cohort  (GSE24080, 556 cases) |
| --- | --- | --- |
| Age (M) | 63.88 (56.93 to 70.81) | 57.98 (50.95 to 64.7 ) |
| Gender (%) |  |  |
| Female | 350 (41%) | 222 (40%) |
| Male | 494 (59%) | 334 (60%) |
| Race (%) |  |  |
| White | 690 (82%) | 494 (89%) |
| Others | 154 (18%) | 62 (11%) |
| Ethnicity (%) |  |  |
| Hispanic or latino | 83 (10%) | NA |
| Not hispanic or latino | 761 (90%) | NA |
| Isotype (%) |  |  |
| FLC | NA | 83 (15%) |
| IgA | NA | 133 (24%) |
| IgD | NA | 3 (1%) |
| IgG | NA | 312 (56%) |
| Nonsecretory | NA | 6 (1%) |
| Not recorded | NA | 19 (3%) |
| B2M (M) mg/l | NA | 3.05 ( 2.1 to 4.9 ) |
| Albumin (M) g/l | NA | 41 ( 37 to 44 ) |
| ISS Stage (%) |  |  |
| I | 285 (34%) | 296 (53%) |
| II | 302 (36%) | 142 (26%) |
| III | 257 (30%) | 118 (21%) |
| CRP (M) mg/l | NA | 4.4 ( 1.17 to 11.03 ) |
| Creatinine (M) mg/l | NA | 1 ( 0.8 to 1.2 ) |
| LDH (M) U/l | NA | 156.5 ( 127 to 199 ) |
| Hemoglobin (M) g/dl | NA | 11.3 ( 9.8 to 12.6 ) |
| EFS status (%) |  |  |
| Non-Event | NA | 309 (56%) |
| Event | NA | 247 (44%) |
| EFS time (M) | NA | 42.62 ( 29.36 to 56.79 ) |
| OS status (%) |  |  |
| Alive | 651 (77%) | 386 (69%) |
| Death | 193 (23%) | 170 (31%) |
| OS time (M) | 27.13 ( 15.37 to 37.42 ) | 48.28 ( 34.83 to 64.19 ) |

M: Median, interquartile range; %: percentage
