# Supplementary material for: Systematic discrimination of the repetitive genome in proximity of ferroptosis genes and a novel prognostic signature correlating with the oncogenic lncRNA CRNDE in multiple myeloma
Source: Front Oncol. 2022 Dec 20;12:1026153. doi: 10.3389/fonc.2022.1026153 (PMC9808058; doi:10.3389/fonc.2022.1026153)
Supplement: Supplementary file 13 [file Table_2.docx]

**Supplementary table 2. 1669 genes were significantly predicting the prognosis of multiple myeloma patients by univariate Cox regression analyses**.

| Gene Symbol | HR | 95CI | P |
| --- | --- | --- | --- |
| AAR2 | 2.8 | 1.96-4 | <0.05 |
| AATF | 3.84 | 2.49-5.94 | <0.05 |
| ABCB10 | 2.16 | 1.65-2.81 | <0.05 |
| ABCE1 | 2.58 | 1.85-3.61 | <0.05 |
| ABCF1 | 2.05 | 1.52-2.78 | <0.05 |
| ABT1 | 2.6 | 1.87-3.61 | <0.05 |
| AC004381.6 | 2.16 | 1.7-2.75 | <0.05 |
| ACAP1 | 1.75 | 1.41-2.17 | <0.05 |
| ACAT2 | 1.98 | 1.48-2.65 | <0.05 |
| ACBD6 | 2.45 | 1.73-3.47 | <0.05 |
| ACBD7 | 2.17 | 1.59-2.96 | <0.05 |
| ACER2 | 0.56 | 0.44-0.71 | <0.05 |
| ACLY | 2.47 | 1.74-3.49 | <0.05 |
| ACOT7 | 2.47 | 2.06-2.96 | <0.05 |
| ACRV1 | 79.14 | 27.98-223.82 | <0.05 |
| ACSS1 | 0.58 | 0.48-0.7 | <0.05 |
| ACTL8 | 1.35 | 1.23-1.48 | <0.05 |
| ACTN4 | 1.91 | 1.51-2.42 | <0.05 |
| ADAM11 | 3.02 | 1.97-4.62 | <0.05 |
| ADAM15 | 1.62 | 1.33-1.97 | <0.05 |
| ADAM18 | 19.43 | 6.94-54.39 | <0.05 |
| ADAM23 | 1.55 | 1.37-1.76 | <0.05 |
| ADAM8 | 1.29 | 1.16-1.43 | <0.05 |
| ADAMTS14 | 2.53 | 2-3.19 | <0.05 |
| ADAMTS20 | 1.68 | 1.37-2.06 | <0.05 |
| ADAT3 | 2.3 | 1.61-3.28 | <0.05 |
| ADCY3 | 1.72 | 1.47-2.01 | <0.05 |
| ADD2 | 1.66 | 1.34-2.05 | <0.05 |
| ADPRHL2 | 2.21 | 1.62-3.02 | <0.05 |
| ADSL | 3.01 | 2.28-3.96 | <0.05 |
| ADSS | 2.01 | 1.55-2.59 | <0.05 |
| AFMID | 1.69 | 1.37-2.1 | <0.05 |
| AGK | 2.7 | 1.95-3.74 | <0.05 |
| AGO2 | 1.63 | 1.33-1.99 | <0.05 |
| AGPAT4 | 2 | 1.56-2.56 | <0.05 |
| AGPS | 3.82 | 2.65-5.52 | <0.05 |
| AHI1 | 0.49 | 0.39-0.61 | <0.05 |
| AHSA1 | 2.14 | 1.6-2.84 | <0.05 |
| AIFM1 | 1.82 | 1.41-2.34 | <0.05 |
| AIMP1 | 2.59 | 1.72-3.89 | <0.05 |
| AK2 | 4.36 | 2.98-6.38 | <0.05 |
| AK9 | 0.35 | 0.24-0.5 | <0.05 |
| AKNA | 1.49 | 1.3-1.71 | <0.05 |
| ALCAM | 0.7 | 0.62-0.8 | <0.05 |
| ALKBH7 | 0.53 | 0.42-0.67 | <0.05 |
| ALOX12B | 28.67 | 7.88-104.33 | <0.05 |
| ALYREF | 3.69 | 2.62-5.2 | <0.05 |
| AMIGO2 | 0.71 | 0.61-0.82 | <0.05 |
| ANAPC11 | 2.67 | 1.83-3.89 | <0.05 |
| ANKRD13A | 0.61 | 0.5-0.75 | <0.05 |
| ANKRD30BL | 2.51 | 1.83-3.42 | <0.05 |
| ANKRD34B | 2.17 | 1.62-2.92 | <0.05 |
| ANLN | 2 | 1.68-2.38 | <0.05 |
| ANP32B | 2.15 | 1.59-2.91 | <0.05 |
| ANP32E | 2.13 | 1.74-2.6 | <0.05 |
| ANXA1 | 1.16 | 1.09-1.22 | <0.05 |
| APEX2 | 2.77 | 2.04-3.77 | <0.05 |
| APH1A | 2.02 | 1.56-2.6 | <0.05 |
| APOBEC3B | 1.53 | 1.37-1.72 | <0.05 |
| ARC | 1.65 | 1.33-2.04 | <0.05 |
| ARF5 | 2.07 | 1.54-2.79 | <0.05 |
| ARHGAP11A | 1.76 | 1.5-2.07 | <0.05 |
| ARHGAP30 | 2.08 | 1.7-2.55 | <0.05 |
| ARHGEF1 | 2.01 | 1.51-2.67 | <0.05 |
| ARHGEF2 | 1.54 | 1.29-1.85 | <0.05 |
| ARHGEF37 | 0.58 | 0.46-0.73 | <0.05 |
| ARNT | 1.75 | 1.4-2.19 | <0.05 |
| ARNTL2 | 1.91 | 1.59-2.28 | <0.05 |
| ARPC2 | 2.39 | 1.72-3.32 | <0.05 |
| ARPC5 | 1.68 | 1.39-2.03 | <0.05 |
| ARVCF | 0.44 | 0.32-0.63 | <0.05 |
| ASB2 | 1.54 | 1.36-1.75 | <0.05 |
| ASB9 | 2.37 | 1.67-3.36 | <0.05 |
| ASF1B | 1.74 | 1.5-2.04 | <0.05 |
| ASNA1 | 2.42 | 1.68-3.5 | <0.05 |
| ASPM | 1.83 | 1.59-2.1 | <0.05 |
| ATAD2 | 2.21 | 1.89-2.58 | <0.05 |
| ATAD3A | 2.39 | 1.79-3.2 | <0.05 |
| ATAD5 | 2.91 | 2.27-3.75 | <0.05 |
| ATIC | 2.26 | 1.69-3.02 | <0.05 |
| ATP1B3 | 1.66 | 1.37-2.02 | <0.05 |
| ATP6V1H | 2.44 | 1.7-3.5 | <0.05 |
| AUNIP | 6.18 | 4.57-8.36 | <0.05 |
| AURKA | 1.95 | 1.66-2.29 | <0.05 |
| AURKB | 1.97 | 1.71-2.27 | <0.05 |
| AURKC | 0.3 | 0.19-0.47 | <0.05 |
| B3GALT4 | 0.58 | 0.46-0.72 | <0.05 |
| B4GALT4 | 0.45 | 0.33-0.61 | <0.05 |
| B4GALT7 | 0.43 | 0.31-0.6 | <0.05 |
| BATF3 | 1.51 | 1.31-1.74 | <0.05 |
| BAZ2B | 0.46 | 0.35-0.59 | <0.05 |
| BCCIP | 3.44 | 2.31-5.14 | <0.05 |
| BCL2L2 | 0.46 | 0.35-0.62 | <0.05 |
| BECN2 | 4879.75 | 155.74-152895.4 | <0.05 |
| BEND4 | 2.23 | 1.64-3.02 | <0.05 |
| BEND6 | 2.41 | 1.77-3.28 | <0.05 |
| BGLAP | 1.33 | 1.2-1.47 | <0.05 |
| BHLHE22 | 3.16 | 2.41-4.14 | <0.05 |
| BHLHE41 | 0.71 | 0.63-0.81 | <0.05 |
| BIRC5 | 1.97 | 1.72-2.26 | <0.05 |
| BLMH | 2.36 | 1.74-3.2 | <0.05 |
| BLNK | 0.65 | 0.56-0.76 | <0.05 |
| BMP6 | 0.71 | 0.62-0.81 | <0.05 |
| BMS1 | 3.11 | 2.04-4.74 | <0.05 |
| BOD1 | 0.5 | 0.37-0.67 | <0.05 |
| BOLA3 | 2.13 | 1.61-2.83 | <0.05 |
| BOP 1.00 | 1.83 | 1.48-2.25 | <0.05 |
| BRCA1 | 2.29 | 1.85-2.84 | <0.05 |
| BRI3BP | 1.63 | 1.35-1.96 | <0.05 |
| BRIP1 | 2.6 | 1.98-3.41 | <0.05 |
| BRIX1 | 2.86 | 2.09-3.9 | <0.05 |
| BTD | 0.44 | 0.34-0.56 | <0.05 |
| BUB1 | 1.96 | 1.69-2.26 | <0.05 |
| BUB1B | 1.78 | 1.55-2.04 | <0.05 |
| BZW1 | 2.06 | 1.53-2.77 | <0.05 |
| C11orf80 | 0.53 | 0.44-0.63 | <0.05 |
| C11orf84 | 1.89 | 1.45-2.48 | <0.05 |
| C12orf48 | 2.79 | 2.22-3.5 | <0.05 |
| C12orf75 | 1.28 | 1.16-1.41 | <0.05 |
| C14orf132 | 12.21 | 4.68-31.85 | <0.05 |
| C14orf80 | 1.8 | 1.51-2.15 | <0.05 |
| C15orf41 | 2.97 | 1.92-4.6 | <0.05 |
| C15orf65 | 0.61 | 0.5-0.75 | <0.05 |
| C16orf59 | 2.32 | 1.93-2.79 | <0.05 |
| C16orf87 | 2.72 | 2.05-3.6 | <0.05 |
| C16orf88 | 2.3 | 1.63-3.24 | <0.05 |
| C16orf92 | 58.96 | 16.02-217.02 | <0.05 |
| C17orf53 | 3.51 | 2.73-4.53 | <0.05 |
| C18orf25 | 1.94 | 1.48-2.54 | <0.05 |
| C18orf54 | 2.59 | 1.91-3.49 | <0.05 |
| C19orf57 | 1.93 | 1.54-2.43 | <0.05 |
| C1D | 2.77 | 1.9-4.06 | <0.05 |
| C1orf106 | 1.36 | 1.22-1.52 | <0.05 |
| C1orf112 | 2.91 | 2.27-3.71 | <0.05 |
| C1orf131 | 2.93 | 2.07-4.16 | <0.05 |
| C1orf35 | 1.94 | 1.48-2.55 | <0.05 |
| C1orf74 | 2.23 | 1.65-3.03 | <0.05 |
| C20orf100 | 1.52 | 1.37-1.68 | <0.05 |
| C21orf58 | 2.11 | 1.73-2.59 | <0.05 |
| C2orf47 | 3.44 | 2.38-4.98 | <0.05 |
| C2orf88 | 0.52 | 0.44-0.62 | <0.05 |
| C3orf18 | 0.54 | 0.44-0.67 | <0.05 |
| C3orf33 | 0.5 | 0.38-0.66 | <0.05 |
| C3orf67 | 3.55 | 2.39-5.27 | <0.05 |
| C4orf46 | 2.12 | 1.66-2.7 | <0.05 |
| C7orf31 | 0.45 | 0.34-0.59 | <0.05 |
| C7orf50 | 0.57 | 0.46-0.72 | <0.05 |
| C7orf73 | 2.5 | 1.74-3.59 | <0.05 |
| C8orf33 | 2.42 | 1.86-3.16 | <0.05 |
| C8orf37 | 3.03 | 1.91-4.8 | <0.05 |
| C8orf76 | 3.41 | 2.31-5.04 | <0.05 |
| CA5B | 1.8 | 1.41-2.3 | <0.05 |
| CAB39L | 0.34 | 0.23-0.5 | <0.05 |
| CACNA1S | 1483.17 | 65.09-33797.59 | <0.05 |
| CACNA2D2 | 0.36 | 0.24-0.55 | <0.05 |
| CACNB1 | 1.99 | 1.63-2.42 | <0.05 |
| CACYBP | 2.08 | 1.66-2.61 | <0.05 |
| CALHM2 | 1.55 | 1.32-1.81 | <0.05 |
| CALM2 | 1.81 | 1.45-2.25 | <0.05 |
| CALN1 | 14.56 | 7.21-29.41 | <0.05 |
| CAMK1D | 0.64 | 0.55-0.75 | <0.05 |
| CAMKV | 19.08 | 6.49-56.08 | <0.05 |
| CAPZB | 2.27 | 1.68-3.07 | <0.05 |
| CARD10 | 6.94 | 3.78-12.74 | <0.05 |
| CARD17 | 1.64 | 1.41-1.89 | <0.05 |
| CARD18 | 2.71 | 1.81-4.06 | <0.05 |
| CARHSP1 | 2.13 | 1.56-2.91 | <0.05 |
| CASC5 | 2 | 1.67-2.41 | <0.05 |
| CBLN2 | 1.46 | 1.26-1.69 | <0.05 |
| CBX2 | 3.32 | 2.59-4.25 | <0.05 |
| CBX3 | 2.88 | 2.07-4 | <0.05 |
| CBX5 | 1.85 | 1.48-2.32 | <0.05 |
| CBX6 | 1.44 | 1.23-1.69 | <0.05 |
| CBX7 | 0.62 | 0.52-0.74 | <0.05 |
| CCDC137 | 1.94 | 1.5-2.52 | <0.05 |
| CCDC138 | 3.02 | 2.31-3.95 | <0.05 |
| CCDC15 | 2.96 | 2.18-4.01 | <0.05 |
| CCDC150 | 6.44 | 4.58-9.04 | <0.05 |
| CCDC192 | 2.53 | 1.81-3.53 | <0.05 |
| CCDC34 | 2.26 | 1.85-2.75 | <0.05 |
| CCDC43 | 2.45 | 1.75-3.43 | <0.05 |
| CCDC69 | 0.56 | 0.44-0.7 | <0.05 |
| CCDC73 | 11 | 4.23-28.64 | <0.05 |
| CCDC86 | 1.46 | 1.25-1.71 | <0.05 |
| CCNA2 | 2.01 | 1.76-2.3 | <0.05 |
| CCNB1 | 1.84 | 1.6-2.11 | <0.05 |
| CCNB2 | 1.77 | 1.55-2.02 | <0.05 |
| CCNE2 | 1.52 | 1.3-1.78 | <0.05 |
| CCNF | 2.95 | 2.36-3.69 | <0.05 |
| CCT2 | 4.03 | 3.03-5.36 | <0.05 |
| CCT3 | 2.04 | 1.66-2.51 | <0.05 |
| CCT4 | 2.86 | 2.1-3.89 | <0.05 |
| CCT5 | 2.07 | 1.62-2.65 | <0.05 |
| CCT6A | 1.86 | 1.44-2.42 | <0.05 |
| CCT7 | 3.24 | 2.18-4.81 | <0.05 |
| CD160 | 1.94 | 1.52-2.49 | <0.05 |
| CD27 | 0.82 | 0.76-0.88 | <0.05 |
| CD274 | 0.61 | 0.5-0.75 | <0.05 |
| CD38 | 0.53 | 0.45-0.63 | <0.05 |
| CD3E | 1.28 | 1.18-1.38 | <0.05 |
| CD40 | 0.68 | 0.59-0.79 | <0.05 |
| CD58 | 1.72 | 1.4-2.11 | <0.05 |
| CD6 | 1.98 | 1.5-2.61 | <0.05 |
| CD70 | 1.36 | 1.2-1.54 | <0.05 |
| CD74 | 0.77 | 0.7-0.85 | <0.05 |
| CDC123 | 3.48 | 2.43-4.98 | <0.05 |
| CDC14B | 0.66 | 0.55-0.79 | <0.05 |
| CDC20 | 1.85 | 1.62-2.11 | <0.05 |
| CDC25A | 2.44 | 2-2.96 | <0.05 |
| CDC25C | 2.42 | 1.95-3.01 | <0.05 |
| CDC42 | 2.4 | 1.82-3.16 | <0.05 |
| CDC42EP1 | 1.47 | 1.32-1.64 | <0.05 |
| CDC42EP2 | 4.07 | 2.79-5.95 | <0.05 |
| CDC42SE1 | 1.76 | 1.45-2.12 | <0.05 |
| CDC45 | 2.05 | 1.76-2.39 | <0.05 |
| CDC5L | 2.35 | 1.68-3.29 | <0.05 |
| CDC6 | 1.9 | 1.61-2.23 | <0.05 |
| CDC7 | 1.89 | 1.58-2.26 | <0.05 |
| CDCA2 | 2.28 | 1.91-2.72 | <0.05 |
| CDCA3 | 2.16 | 1.76-2.67 | <0.05 |
| CDCA4 | 3.04 | 2.37-3.88 | <0.05 |
| CDCA5 | 1.82 | 1.59-2.07 | <0.05 |
| CDCA7 | 2.14 | 1.79-2.57 | <0.05 |
| CDCA8 | 1.94 | 1.69-2.23 | <0.05 |
| CDH9 | 1.72 | 1.4-2.13 | <0.05 |
| CDK1 | 1.79 | 1.56-2.05 | <0.05 |
| CDK2 | 2.28 | 1.76-2.96 | <0.05 |
| CDK4 | 2.3 | 1.62-3.27 | <0.05 |
| CDKN2A | 2.28 | 1.83-2.84 | <0.05 |
| CDKN2C | 1.31 | 1.2-1.43 | <0.05 |
| CDKN3 | 2.14 | 1.83-2.5 | <0.05 |
| CDON | 2.68 | 1.93-3.72 | <0.05 |
| CDR2L | 3.59 | 2.17-5.95 | <0.05 |
| CDT1 | 2.09 | 1.78-2.45 | <0.05 |
| CDYL | 1.77 | 1.39-2.25 | <0.05 |
| CEACAM1 | 0.61 | 0.52-0.71 | <0.05 |
| CECR1 | 0.7 | 0.63-0.79 | <0.05 |
| CENPA | 2.03 | 1.74-2.36 | <0.05 |
| CENPE | 2.69 | 2.21-3.28 | <0.05 |
| CENPF | 1.88 | 1.64-2.15 | <0.05 |
| CENPH | 2.17 | 1.8-2.62 | <0.05 |
| CENPI | 3.09 | 2.44-3.91 | <0.05 |
| CENPK | 1.99 | 1.63-2.42 | <0.05 |
| CENPL | 2.89 | 2.23-3.76 | <0.05 |
| CENPM | 2.19 | 1.85-2.59 | <0.05 |
| CENPN | 2.75 | 2.14-3.53 | <0.05 |
| CENPO | 3.41 | 2.57-4.51 | <0.05 |
| CENPP | 4.19 | 2.83-6.19 | <0.05 |
| CENPQ | 1.74 | 1.4-2.16 | <0.05 |
| CENPS | 2.5 | 1.74-3.61 | <0.05 |
| CENPU | 1.85 | 1.56-2.18 | <0.05 |
| CENPW | 1.96 | 1.69-2.29 | <0.05 |
| CEP120 | 0.48 | 0.36-0.65 | <0.05 |
| CEP55 | 1.85 | 1.6-2.14 | <0.05 |
| CEP72 | 3.22 | 2.37-4.4 | <0.05 |
| CEP78 | 2.59 | 1.86-3.62 | <0.05 |
| CEP85 | 1.9 | 1.46-2.47 | <0.05 |
| CFAP77 | 38.83 | 8.15-184.9 | <0.05 |
| CFH | 1.31 | 1.17-1.47 | <0.05 |
| CFHR3 | 1.64 | 1.37-1.96 | <0.05 |
| CFHR4 | 4.51 | 2.69-7.59 | <0.05 |
| CFL1 | 2.25 | 1.65-3.06 | <0.05 |
| CHAF1A | 2.27 | 1.87-2.74 | <0.05 |
| CHAF1B | 2.2 | 1.78-2.71 | <0.05 |
| CHCHD2 | 2.34 | 1.73-3.18 | <0.05 |
| CHCHD3 | 2.91 | 1.96-4.33 | <0.05 |
| CHD1L | 2.11 | 1.68-2.65 | <0.05 |
| CHEK1 | 2.63 | 2.11-3.27 | <0.05 |
| CHEK2 | 1.8 | 1.41-2.29 | <0.05 |
| CHML | 1.53 | 1.31-1.78 | <0.05 |
| CHMP3 | 0.36 | 0.23-0.55 | <0.05 |
| CHRM3 | 1.94 | 1.61-2.35 | <0.05 |
| CHTF18 | 1.95 | 1.59-2.39 | <0.05 |
| CHTOP | 1.93 | 1.46-2.57 | <0.05 |
| CIAPIN1 | 2.42 | 1.69-3.46 | <0.05 |
| CIB2 | 0.61 | 0.5-0.75 | <0.05 |
| CIRBP | 0.5 | 0.39-0.64 | <0.05 |
| CISD3 | 2.08 | 1.56-2.76 | <0.05 |
| CISH | 0.7 | 0.61-0.81 | <0.05 |
| CIT | 3.22 | 2.46-4.22 | <0.05 |
| CKAP2 | 1.57 | 1.3-1.91 | <0.05 |
| CKAP2L | 1.98 | 1.65-2.37 | <0.05 |
| CKAP5 | 1.98 | 1.55-2.55 | <0.05 |
| CKLF | 1.58 | 1.3-1.91 | <0.05 |
| CKLF.CMTM1 | 9.43 | 3.91-22.78 | <0.05 |
| CKS1B | 2.02 | 1.72-2.38 | <0.05 |
| CKS2 | 1.45 | 1.26-1.66 | <0.05 |
| CLEC2L | 2.25 | 1.81-2.78 | <0.05 |
| CLEC7A | 1.24 | 1.14-1.34 | <0.05 |
| CLPTM1 | 0.47 | 0.35-0.64 | <0.05 |
| CLSPN | 2.79 | 2.26-3.45 | <0.05 |
| CLU | 1.45 | 1.28-1.63 | <0.05 |
| CMSS1 | 2.04 | 1.55-2.68 | <0.05 |
| CMTM1 | 2.91 | 1.86-4.55 | <0.05 |
| CNIH4 | 2.17 | 1.62-2.9 | <0.05 |
| CNTNAP4 | 1.57 | 1.3-1.9 | <0.05 |
| COA6 | 2.04 | 1.65-2.51 | <0.05 |
| COBLL1 | 0.68 | 0.58-0.8 | <0.05 |
| COLGALT2 | 1.79 | 1.41-2.28 | <0.05 |
| COMMD1 | 2.57 | 1.77-3.73 | <0.05 |
| COMMD5 | 2.55 | 1.8-3.6 | <0.05 |
| COPS3 | 3.93 | 2.72-5.7 | <0.05 |
| COPS5 | 2.53 | 1.84-3.49 | <0.05 |
| CORO1A | 1.6 | 1.39-1.84 | <0.05 |
| COX6C | 2.06 | 1.57-2.69 | <0.05 |
| CPQ | 0.69 | 0.62-0.77 | <0.05 |
| CPSF3 | 2.2 | 1.58-3.07 | <0.05 |
| CPSF6 | 3.19 | 2.14-4.78 | <0.05 |
| CREB3L3 | 2.9 | 1.84-4.55 | <0.05 |
| CREBL2 | 0.45 | 0.35-0.59 | <0.05 |
| CRELD1 | 0.52 | 0.41-0.67 | <0.05 |
| CRIP1 | 1.28 | 1.16-1.41 | <0.05 |
| CRIP2 | 1.67 | 1.37-2.04 | <0.05 |
| CRISPLD1 | 2.29 | 1.68-3.14 | <0.05 |
| CRTC3 | 0.56 | 0.45-0.71 | <0.05 |
| CRYL1 | 0.66 | 0.57-0.77 | <0.05 |
| CSAG1 | 1.33 | 1.2-1.48 | <0.05 |
| CSAG3 | 1.63 | 1.4-1.9 | <0.05 |
| CSE1L | 2.29 | 1.74-3.02 | <0.05 |
| CSF2 | 4.14 | 2.74-6.26 | <0.05 |
| CSGALNACT1 | 0.71 | 0.63-0.8 | <0.05 |
| CSNK1G3 | 0.51 | 0.41-0.63 | <0.05 |
| CSRNP3 | 7.43 | 3.16-17.5 | <0.05 |
| CSTF2 | 2.67 | 2.1-3.39 | <0.05 |
| CT45A10 | 3.58 | 2.34-5.46 | <0.05 |
| CT45A5 | 60557.65 | 963.42-3806456.67 | <0.05 |
| CTAG1A | 12.54 | 5.87-26.78 | <0.05 |
| CTAG1B | 6.23 | 3.26-11.93 | <0.05 |
| CTAG2 | 1.27 | 1.18-1.36 | <0.05 |
| CTD.2579N5.1 | 4.63 | 2.48-8.65 | <0.05 |
| CTH | 1.41 | 1.22-1.63 | <0.05 |
| CTNNAL1 | 1.46 | 1.26-1.69 | <0.05 |
| CTPS1 | 2.13 | 1.7-2.67 | <0.05 |
| CTSS | 0.61 | 0.49-0.75 | <0.05 |
| CTTN | 0.72 | 0.64-0.81 | <0.05 |
| CTXN1 | 3.16 | 2.03-4.92 | <0.05 |
| CUX1 | 0.45 | 0.32-0.63 | <0.05 |
| CXXC5 | 0.51 | 0.4-0.65 | <0.05 |
| CYB5D2 | 0.54 | 0.43-0.69 | <0.05 |
| CYC1 | 2.65 | 1.91-3.69 | <0.05 |
| CYP19A1 | 34.81 | 8.47-143.08 | <0.05 |
| CYP1A1 | 55.82 | 10.51-296.43 | <0.05 |
| CYP2C18 | 160.27 | 19.32-1329.84 | <0.05 |
| DAAM1 | 1.65 | 1.35-2.03 | <0.05 |
| DACT1 | 2.04 | 1.6-2.6 | <0.05 |
| DAND5 | 19.74 | 6.37-61.23 | <0.05 |
| DAP3 | 2.09 | 1.56-2.8 | <0.05 |
| DAZAP1 | 5.26 | 3.19-8.68 | <0.05 |
| DBF4B | 3.22 | 2.24-4.62 | <0.05 |
| DCAF13 | 3.17 | 2.33-4.31 | <0.05 |
| DCC | 1.36 | 1.21-1.53 | <0.05 |
| DCK | 1.74 | 1.47-2.06 | <0.05 |
| DCLRE1B | 3.19 | 2.28-4.45 | <0.05 |
| DCTPP1 | 2.02 | 1.53-2.65 | <0.05 |
| DCUN1D5 | 1.9 | 1.45-2.5 | <0.05 |
| DDHD2 | 0.51 | 0.38-0.67 | <0.05 |
| DDIT4 | 1.37 | 1.24-1.52 | <0.05 |
| DDX21 | 1.8 | 1.42-2.3 | <0.05 |
| DDX49 | 2.66 | 2.01-3.52 | <0.05 |
| DDX5 | 0.57 | 0.46-0.71 | <0.05 |
| DDX52 | 3.44 | 2.3-5.14 | <0.05 |
| DDX58 | 0.61 | 0.5-0.75 | <0.05 |
| DEDD | 2.28 | 1.69-3.07 | <0.05 |
| DENND5B | 0.57 | 0.46-0.7 | <0.05 |
| DENR | 2.36 | 1.63-3.41 | <0.05 |
| DEPDC1 | 2.06 | 1.72-2.46 | <0.05 |
| DEPDC1B | 2.07 | 1.74-2.46 | <0.05 |
| DGKH | 4.02 | 2.78-5.81 | <0.05 |
| DHFR | 1.76 | 1.42-2.18 | <0.05 |
| DHRS12 | 0.41 | 0.28-0.6 | <0.05 |
| DHX37 | 3.1 | 2.15-4.45 | <0.05 |
| DHX9 | 2.57 | 1.91-3.46 | <0.05 |
| DIAPH3 | 3.49 | 2.65-4.6 | <0.05 |
| DKC1 | 2.1 | 1.65-2.68 | <0.05 |
| DLG3 | 2.18 | 1.69-2.82 | <0.05 |
| DLGAP1 | 778.92 | 48.76-12442.43 | <0.05 |
| DLGAP5 | 2.06 | 1.8-2.37 | <0.05 |
| DMC1 | 2.71 | 2.13-3.43 | <0.05 |
| DMRT3 | 6.09 | 2.91-12.72 | <0.05 |
| DNA2 | 3.05 | 2.34-3.96 | <0.05 |
| DNAJB2 | 0.5 | 0.38-0.67 | <0.05 |
| DNAJB9 | 0.58 | 0.48-0.7 | <0.05 |
| DNAJC4 | 0.35 | 0.27-0.46 | <0.05 |
| DNAJC8 | 3.96 | 2.68-5.86 | <0.05 |
| DNAJC9 | 3.24 | 2.16-4.86 | <0.05 |
| DNMBP | 0.44 | 0.34-0.57 | <0.05 |
| DNMT1 | 2.2 | 1.76-2.76 | <0.05 |
| DNMT3B | 1.78 | 1.5-2.11 | <0.05 |
| DOCK11 | 1.43 | 1.26-1.63 | <0.05 |
| DOCK7 | 0.41 | 0.29-0.58 | <0.05 |
| DONSON | 2.21 | 1.7-2.87 | <0.05 |
| DPF1 | 3.69 | 2.44-5.59 | <0.05 |
| DPF3 | 1.49 | 1.29-1.71 | <0.05 |
| DPH6 | 3.32 | 1.98-5.55 | <0.05 |
| DPH7 | 0.54 | 0.41-0.7 | <0.05 |
| DPP7 | 0.75 | 0.67-0.85 | <0.05 |
| DPPA2 | 1.87 | 1.44-2.43 | <0.05 |
| DPY19L2 | 1.52 | 1.27-1.81 | <0.05 |
| DR1 | 2.07 | 1.54-2.78 | <0.05 |
| DRG1 | 3.12 | 2.22-4.39 | <0.05 |
| DRP2 | 28.24 | 14.38-55.44 | <0.05 |
| DSCC1 | 2.48 | 2.12-2.89 | <0.05 |
| DTL | 1.96 | 1.71-2.26 | <0.05 |
| DTYMK | 3.02 | 2.25-4.06 | <0.05 |
| DUSP12 | 2.12 | 1.63-2.76 | <0.05 |
| DUSP14 | 1.86 | 1.47-2.36 | <0.05 |
| DUSP26 | 0.79 | 0.73-0.87 | <0.05 |
| E2F1 | 1.7 | 1.49-1.95 | <0.05 |
| E2F2 | 1.55 | 1.39-1.72 | <0.05 |
| E2F8 | 2.12 | 1.81-2.47 | <0.05 |
| EBNA1BP2 | 2.76 | 1.99-3.82 | <0.05 |
| EBP | 2.44 | 1.77-3.36 | <0.05 |
| ECE1 | 0.46 | 0.36-0.59 | <0.05 |
| ECHDC2 | 0.68 | 0.58-0.8 | <0.05 |
| ECT2 | 1.87 | 1.5-2.35 | <0.05 |
| EFCAB11 | 3.72 | 2.13-6.51 | <0.05 |
| EFNA3 | 3.68 | 2.5-5.43 | <0.05 |
| EFTUD2 | 5.15 | 3.39-7.83 | <0.05 |
| EGFLAM | 7.2 | 3.52-14.72 | <0.05 |
| EIF2S1 | 2.88 | 2.1-3.95 | <0.05 |
| EIF4A2 | 0.46 | 0.35-0.61 | <0.05 |
| EIF4A3 | 1.74 | 1.45-2.09 | <0.05 |
| EIF4EBP1 | 1.5 | 1.33-1.69 | <0.05 |
| EIF6 | 2.66 | 1.97-3.57 | <0.05 |
| ELK1 | 3.38 | 2.42-4.71 | <0.05 |
| ELK4 | 1.83 | 1.42-2.36 | <0.05 |
| ELOC | 3.77 | 2.57-5.51 | <0.05 |
| ELOVL6 | 2.02 | 1.71-2.39 | <0.05 |
| EME1 | 2.27 | 1.87-2.75 | <0.05 |
| EMP1 | 1.39 | 1.23-1.57 | <0.05 |
| ENAM | 1.31 | 1.17-1.47 | <0.05 |
| ENO1 | 2.59 | 2.11-3.18 | <0.05 |
| ENSA | 1.86 | 1.43-2.41 | <0.05 |
| ENTPD4 | 0.63 | 0.52-0.77 | <0.05 |
| ENY2 | 5.49 | 3.7-8.14 | <0.05 |
| EPB41L4A | 0.52 | 0.43-0.63 | <0.05 |
| EPHB2 | 3.31 | 2.23-4.91 | <0.05 |
| EPN1 | 0.42 | 0.3-0.6 | <0.05 |
| EPOP | 1.7 | 1.39-2.08 | <0.05 |
| EPS8L3 | 7.81 | 3.33-18.28 | <0.05 |
| ERCC6L | 2.54 | 1.95-3.31 | <0.05 |
| ERH | 2.8 | 2.01-3.9 | <0.05 |
| ERI1 | 2.82 | 2.06-3.86 | <0.05 |
| ESCO2 | 3.64 | 2.77-4.78 | <0.05 |
| ESF1 | 2.5 | 1.79-3.47 | <0.05 |
| ESPL1 | 2.43 | 2.02-2.91 | <0.05 |
| ESYT1 | 1.79 | 1.41-2.27 | <0.05 |
| ETV4 | 1.82 | 1.54-2.16 | <0.05 |
| EXD3 | 0.43 | 0.3-0.61 | <0.05 |
| EXO1 | 2.16 | 1.86-2.51 | <0.05 |
| EXOSC4 | 2.06 | 1.61-2.62 | <0.05 |
| EXOSC9 | 2.85 | 1.99-4.08 | <0.05 |
| EYA1 | 1.49 | 1.26-1.76 | <0.05 |
| EZH2 | 2.04 | 1.71-2.43 | <0.05 |
| FABP5 | 1.56 | 1.39-1.75 | <0.05 |
| FAM110A | 1.89 | 1.45-2.47 | <0.05 |
| FAM111B | 1.78 | 1.54-2.05 | <0.05 |
| FAM126A | 1.65 | 1.38-1.97 | <0.05 |
| FAM129A | 1.24 | 1.13-1.36 | <0.05 |
| FAM13A | 0.66 | 0.57-0.77 | <0.05 |
| FAM160A1 | 0.32 | 0.22-0.45 | <0.05 |
| FAM167A | 6.71 | 4.36-10.33 | <0.05 |
| FAM178B | 1.65 | 1.34-2.04 | <0.05 |
| FAM207A | 1.89 | 1.47-2.43 | <0.05 |
| FAM210A | 2.57 | 1.84-3.6 | <0.05 |
| FAM227A | 4.32 | 2.99-6.23 | <0.05 |
| FAM237A | 55.27 | 11.15-273.9 | <0.05 |
| FAM3B | 1.34 | 1.19-1.52 | <0.05 |
| FAM49B | 2.69 | 2.12-3.42 | <0.05 |
| FAM57B | 3.58 | 2.44-5.25 | <0.05 |
| FAM64A | 1.74 | 1.44-2.11 | <0.05 |
| FAM72A | 4.61 | 3.38-6.27 | <0.05 |
| FAM72B | 3.68 | 2.8-4.83 | <0.05 |
| FAM72C | 4.05 | 3.02-5.42 | <0.05 |
| FAM83D | 2.3 | 1.87-2.83 | <0.05 |
| FAM92A1 | 2.43 | 1.67-3.53 | <0.05 |
| FAM9B | 2.54 | 1.93-3.35 | <0.05 |
| FANCA | 1.98 | 1.61-2.44 | <0.05 |
| FANCB | 4.25 | 3.07-5.88 | <0.05 |
| FANCI | 1.83 | 1.55-2.16 | <0.05 |
| FANCM | 2.65 | 1.84-3.81 | <0.05 |
| FARSB | 2.25 | 1.68-3.01 | <0.05 |
| FAS | 1.35 | 1.22-1.49 | <0.05 |
| FASN | 1.77 | 1.45-2.16 | <0.05 |
| FBXL17 | 0.45 | 0.33-0.63 | <0.05 |
| FBXO2 | 0.57 | 0.46-0.7 | <0.05 |
| FBXO40 | 2.72 | 2.01-3.69 | <0.05 |
| FBXO43 | 2.66 | 2.03-3.49 | <0.05 |
| FBXO44 | 0.56 | 0.43-0.71 | <0.05 |
| FBXO5 | 2.1 | 1.62-2.72 | <0.05 |
| FBXW7 | 0.64 | 0.53-0.78 | <0.05 |
| FCGR2B | 0.78 | 0.7-0.87 | <0.05 |
| FCGRT | 0.69 | 0.61-0.79 | <0.05 |
| FCMR | 1.41 | 1.29-1.53 | <0.05 |
| FDPS | 2.69 | 2.02-3.57 | <0.05 |
| FEN1 | 2.27 | 1.87-2.75 | <0.05 |
| FERMT1 | 2.86 | 2.04-4.01 | <0.05 |
| FGD2 | 0.69 | 0.59-0.8 | <0.05 |
| FGF13 | 2.16 | 1.57-2.97 | <0.05 |
| FH | 2.19 | 1.74-2.76 | <0.05 |
| FHL2 | 2.87 | 1.9-4.32 | <0.05 |
| FHOD1 | 2.06 | 1.7-2.51 | <0.05 |
| FIGNL1 | 2.15 | 1.76-2.63 | <0.05 |
| FIP1L1 | 3.64 | 2.46-5.38 | <0.05 |
| FKBP1A | 2.55 | 1.92-3.39 | <0.05 |
| FLAD1 | 2.16 | 1.63-2.87 | <0.05 |
| FLNA | 1.43 | 1.31-1.56 | <0.05 |
| FLVCR1 | 2.4 | 1.79-3.21 | <0.05 |
| FMR1NB | 1.29 | 1.17-1.43 | <0.05 |
| FNDC3B | 0.46 | 0.37-0.58 | <0.05 |
| FOXD1 | 2.46 | 1.97-3.06 | <0.05 |
| FOXD2 | 13.88 | 4.75-40.57 | <0.05 |
| FOXM1 | 2.03 | 1.74-2.36 | <0.05 |
| FOXRED1 | 2.13 | 1.56-2.92 | <0.05 |
| FRG1 | 2.9 | 1.99-4.22 | <0.05 |
| FRMD5 | 14.45 | 6.32-33.05 | <0.05 |
| FYN | 1.5 | 1.3-1.73 | <0.05 |
| GAB2 | 0.66 | 0.56-0.77 | <0.05 |
| GABARAP | 0.41 | 0.3-0.56 | <0.05 |
| GABRA3 | 1.58 | 1.34-1.87 | <0.05 |
| GABRB2 | 1.68 | 1.37-2.06 | <0.05 |
| GABRG3 | 5.03 | 2.52-10.04 | <0.05 |
| GAGE2A | 1.37 | 1.22-1.53 | <0.05 |
| GAGE2E | 54.81 | 10.61-283.07 | <0.05 |
| GAL | 2.88 | 1.95-4.25 | <0.05 |
| GALE | 1.71 | 1.37-2.14 | <0.05 |
| GALNT11 | 0.55 | 0.45-0.68 | <0.05 |
| GALNT8 | 52.8 | 13.07-213.23 | <0.05 |
| GALR2 | 4.11 | 2.25-7.51 | <0.05 |
| GAP43 | 1.65 | 1.36-2 | <0.05 |
| GAR1 | 3.02 | 2.25-4.05 | <0.05 |
| GATA4 | 4.03 | 2.57-6.32 | <0.05 |
| GBA2 | 0.52 | 0.39-0.69 | <0.05 |
| GDPD2 | 13.92 | 7.51-25.81 | <0.05 |
| GEMIN2 | 1.92 | 1.46-2.53 | <0.05 |
| GEMIN6 | 1.96 | 1.48-2.61 | <0.05 |
| GFRA3 | 118.48 | 18.7-750.63 | <0.05 |
| GGA2 | 0.45 | 0.33-0.6 | <0.05 |
| GGH | 1.43 | 1.26-1.62 | <0.05 |
| GGT7 | 0.63 | 0.54-0.73 | <0.05 |
| GINM1 | 0.6 | 0.49-0.74 | <0.05 |
| GINS1 | 2.31 | 1.93-2.76 | <0.05 |
| GINS2 | 2.32 | 1.93-2.79 | <0.05 |
| GINS3 | 3.61 | 2.67-4.88 | <0.05 |
| GINS4 | 3.77 | 2.91-4.89 | <0.05 |
| GJA5 | 7.43 | 3.15-17.56 | <0.05 |
| GJC1 | 35.38 | 11.79-106.22 | <0.05 |
| GLB1L3 | 2.83 | 1.84-4.36 | <0.05 |
| GLIPR2 | 1.62 | 1.41-1.87 | <0.05 |
| GLRX2 | 1.85 | 1.46-2.34 | <0.05 |
| GLRX3 | 4.26 | 2.84-6.37 | <0.05 |
| GLRX5 | 2.15 | 1.55-2.98 | <0.05 |
| GLTSCR2 | 0.62 | 0.53-0.72 | <0.05 |
| GLUD1 | 3.62 | 2.6-5.02 | <0.05 |
| GMNN | 2.5 | 2.04-3.05 | <0.05 |
| GNAI3 | 2.82 | 1.89-4.22 | <0.05 |
| GNG13 | 39.39 | 13.36-116.18 | <0.05 |
| GNG7 | 0.58 | 0.47-0.71 | <0.05 |
| GNL2 | 3.16 | 2.17-4.61 | <0.05 |
| GOLGA8N | 0.35 | 0.22-0.54 | <0.05 |
| GOLGA8R | 0.11 | 0.05-0.25 | <0.05 |
| GOLM1 | 1.32 | 1.2-1.45 | <0.05 |
| GORASP1 | 0.43 | 0.31-0.61 | <0.05 |
| GPAA1 | 2.05 | 1.62-2.59 | <0.05 |
| GPATCH4 | 1.78 | 1.43-2.22 | <0.05 |
| GPCPD1 | 0.51 | 0.42-0.62 | <0.05 |
| GPD1L | 0.58 | 0.47-0.72 | <0.05 |
| GPR108 | 0.43 | 0.32-0.59 | <0.05 |
| GPR132 | 1.73 | 1.54-1.94 | <0.05 |
| GPR160 | 0.68 | 0.59-0.78 | <0.05 |
| GPR63 | 1.45 | 1.31-1.59 | <0.05 |
| GPS1 | 2.57 | 1.76-3.77 | <0.05 |
| GPT2 | 1.38 | 1.21-1.59 | <0.05 |
| GRAMD1C | 0.65 | 0.56-0.77 | <0.05 |
| GRB14 | 1.49 | 1.35-1.65 | <0.05 |
| GRHL1 | 0.55 | 0.44-0.68 | <0.05 |
| GRIP1 | 0.6 | 0.5-0.73 | <0.05 |
| GRM7 | 69.44 | 12.59-382.93 | <0.05 |
| GRPEL2 | 2.18 | 1.66-2.86 | <0.05 |
| GRWD1 | 2.61 | 1.9-3.57 | <0.05 |
| GSG2 | 1.4 | 1.24-1.59 | <0.05 |
| GSTCD | 2.52 | 1.74-3.65 | <0.05 |
| GTF2H3 | 2.36 | 1.75-3.18 | <0.05 |
| GTPBP4 | 2.39 | 1.77-3.22 | <0.05 |
| GTSE1 | 2.23 | 1.89-2.62 | <0.05 |
| GTSF1 | 1.27 | 1.16-1.4 | <0.05 |
| GUK1 | 2.11 | 1.54-2.89 | <0.05 |
| GZMB | 1.3 | 1.17-1.45 | <0.05 |
| GZMH | 1.31 | 1.19-1.45 | <0.05 |
| H2AFX | 1.84 | 1.51-2.23 | <0.05 |
| H2AFZ | 2.66 | 2.07-3.42 | <0.05 |
| H3F3A | 1.98 | 1.57-2.49 | <0.05 |
| HADH | 1.71 | 1.39-2.1 | <0.05 |
| HAT1 | 2.44 | 1.75-3.41 | <0.05 |
| HAUS1 | 2.34 | 1.79-3.05 | <0.05 |
| HAUS7 | 2.46 | 1.7-3.58 | <0.05 |
| HAUS8 | 2.79 | 1.93-4.03 | <0.05 |
| HBEGF | 1.37 | 1.21-1.54 | <0.05 |
| HDAC2 | 2.65 | 1.83-3.84 | <0.05 |
| HDGF | 1.88 | 1.56-2.27 | <0.05 |
| HDLBP | 0.57 | 0.47-0.7 | <0.05 |
| HEATR1 | 2.02 | 1.57-2.59 | <0.05 |
| HECW2 | 5.86 | 3.26-10.56 | <0.05 |
| HELLS | 2.86 | 2.29-3.57 | <0.05 |
| HES7 | 2.5 | 1.75-3.56 | <0.05 |
| HHAT | 1.92 | 1.55-2.39 | <0.05 |
| HIST1H2AI | 1.3 | 1.17-1.46 | <0.05 |
| HIST1H2BH | 1.5 | 1.29-1.75 | <0.05 |
| HIST1H3G | 2.41 | 1.78-3.25 | <0.05 |
| HJURP | 1.8 | 1.56-2.06 | <0.05 |
| HK2 | 1.33 | 1.19-1.49 | <0.05 |
| HKDC1 | 1.96 | 1.54-2.49 | <0.05 |
| HLA.C | 0.67 | 0.56-0.79 | <0.05 |
| HLA.DOB | 0.65 | 0.59-0.71 | <0.05 |
| HLA.E | 0.57 | 0.46-0.69 | <0.05 |
| HMGB2 | 1.98 | 1.65-2.37 | <0.05 |
| HMGB3 | 1.64 | 1.46-1.85 | <0.05 |
| HMGCS1 | 2.04 | 1.54-2.69 | <0.05 |
| HMGN2 | 1.96 | 1.48-2.61 | <0.05 |
| HMGN5 | 1.49 | 1.29-1.71 | <0.05 |
| HMGXB4 | 3.87 | 2.83-5.29 | <0.05 |
| HMMR | 1.93 | 1.64-2.27 | <0.05 |
| HN1 | 1.9 | 1.57-2.29 | <0.05 |
| HNRNPAB | 2.45 | 1.82-3.3 | <0.05 |
| HNRNPC | 3.55 | 2.44-5.16 | <0.05 |
| HNRNPD | 2.91 | 1.9-4.46 | <0.05 |
| HNRNPF | 2.74 | 1.98-3.8 | <0.05 |
| HNRNPH2 | 1.88 | 1.44-2.46 | <0.05 |
| HNRNPK | 6.06 | 3.57-10.28 | <0.05 |
| HNRNPR | 4.41 | 3.02-6.46 | <0.05 |
| HNRNPU | 2.23 | 1.66-3 | <0.05 |
| HPCA | 4.18 | 2.34-7.46 | <0.05 |
| HPCAL4 | 1.86 | 1.43-2.43 | <0.05 |
| HPDL | 1.5 | 1.32-1.71 | <0.05 |
| HPGD | 1.66 | 1.34-2.05 | <0.05 |
| HPRT1 | 2.41 | 1.99-2.91 | <0.05 |
| HPSE | 1.68 | 1.42-1.99 | <0.05 |
| HSD11B1 | 1.32 | 1.19-1.46 | <0.05 |
| HSD17B10 | 2.26 | 1.65-3.1 | <0.05 |
| HSD17B6 | 2.09 | 1.53-2.84 | <0.05 |
| HSP90AA1 | 1.78 | 1.49-2.12 | <0.05 |
| HSP90AB1 | 2.05 | 1.54-2.73 | <0.05 |
| HTATSF1 | 1.96 | 1.54-2.49 | <0.05 |
| HTR2C | 1.47 | 1.28-1.69 | <0.05 |
| ICA1L | 3.8 | 2.22-6.51 | <0.05 |
| ICAM3 | 0.63 | 0.54-0.74 | <0.05 |
| ICT1 | 2.89 | 2.07-4.03 | <0.05 |
| IER5L | 2.11 | 1.74-2.55 | <0.05 |
| IFFO2 | 1.37 | 1.2-1.56 | <0.05 |
| IGF2BP1 | 3.65 | 2.3-5.78 | <0.05 |
| IGFBP1 | 15.85 | 6.99-35.91 | <0.05 |
| IGFBP7 | 1.31 | 1.2-1.44 | <0.05 |
| IL24 | 1.7 | 1.49-1.95 | <0.05 |
| IL27RA | 1.32 | 1.18-1.48 | <0.05 |
| IL32 | 1.45 | 1.3-1.61 | <0.05 |
| ILDR2 | 2.04 | 1.53-2.72 | <0.05 |
| ILF2 | 2.85 | 2.17-3.75 | <0.05 |
| IMMP2L | 0.45 | 0.34-0.59 | <0.05 |
| IMPA1 | 2.33 | 1.68-3.22 | <0.05 |
| INCENP | 2.37 | 1.86-3.02 | <0.05 |
| ING2 | 1.56 | 1.3-1.88 | <0.05 |
| INTS7 | 2.04 | 1.59-2.63 | <0.05 |
| IPO9 | 2.16 | 1.61-2.9 | <0.05 |
| IQCF1 | 3.62 | 2.12-6.2 | <0.05 |
| IQGAP3 | 2.04 | 1.72-2.42 | <0.05 |
| ISCU | 0.51 | 0.4-0.65 | <0.05 |
| ISG20L2 | 2.07 | 1.57-2.72 | <0.05 |
| ISY1 | 3.18 | 1.98-5.12 | <0.05 |
| ITGA6 | 0.73 | 0.65-0.81 | <0.05 |
| ITGA7 | 2.08 | 1.67-2.58 | <0.05 |
| ITGB1BP1 | 2.61 | 1.89-3.61 | <0.05 |
| ITGB1BP2 | 4.39 | 2.97-6.49 | <0.05 |
| ITM2B | 0.66 | 0.57-0.77 | <0.05 |
| ITPRIP | 0.65 | 0.57-0.73 | <0.05 |
| ITPRIPL1 | 1.54 | 1.3-1.82 | <0.05 |
| JAK2 | 0.58 | 0.46-0.73 | <0.05 |
| JAKMIP2 | 1.85 | 1.53-2.24 | <0.05 |
| KCNAB3 | 3.22 | 2.03-5.11 | <0.05 |
| KCNH8 | 2.33 | 1.72-3.15 | <0.05 |
| KCNK1 | 1.7 | 1.35-2.13 | <0.05 |
| KCNK13 | 1.45 | 1.28-1.64 | <0.05 |
| KCNK2 | 2.07 | 1.62-2.64 | <0.05 |
| KCNMB3 | 0.41 | 0.3-0.57 | <0.05 |
| KCTD3 | 1.63 | 1.32-2 | <0.05 |
| KDELC1 | 2.15 | 1.56-2.95 | <0.05 |
| KDELR1 | 0.53 | 0.41-0.68 | <0.05 |
| KDM1A | 3.87 | 2.56-5.85 | <0.05 |
| KDM4C | 0.33 | 0.22-0.51 | <0.05 |
| KDM7A | 0.6 | 0.49-0.74 | <0.05 |
| KHDRBS1 | 2.78 | 1.89-4.1 | <0.05 |
| KHDRBS3 | 1.85 | 1.42-2.39 | <0.05 |
| KHSRP | 2.62 | 1.78-3.85 | <0.05 |
| KIAA0141 | 0.49 | 0.37-0.66 | <0.05 |
| KIAA1147 | 0.58 | 0.47-0.71 | <0.05 |
| KIAA1191 | 0.32 | 0.23-0.45 | <0.05 |
| KIAA1211 | 10.69 | 5.6-20.38 | <0.05 |
| KIAA1328 | 3.66 | 2.13-6.29 | <0.05 |
| KIAA1370 | 0.56 | 0.45-0.69 | <0.05 |
| KIAA1429 | 3.47 | 2.33-5.18 | <0.05 |
| KIAA1841 | 2.44 | 1.73-3.45 | <0.05 |
| KIF11 | 1.96 | 1.69-2.28 | <0.05 |
| KIF14 | 2.49 | 2.08-2.97 | <0.05 |
| KIF15 | 2.18 | 1.82-2.61 | <0.05 |
| KIF18A | 2.22 | 1.79-2.75 | <0.05 |
| KIF18B | 2.12 | 1.82-2.47 | <0.05 |
| KIF19 | 0.72 | 0.65-0.81 | <0.05 |
| KIF20A | 1.92 | 1.63-2.27 | <0.05 |
| KIF20B | 2.54 | 1.97-3.26 | <0.05 |
| KIF21B | 1.47 | 1.33-1.61 | <0.05 |
| KIF22 | 2.37 | 1.93-2.9 | <0.05 |
| KIF23 | 2.09 | 1.78-2.45 | <0.05 |
| KIF24 | 2.04 | 1.57-2.65 | <0.05 |
| KIF2C | 2.2 | 1.89-2.57 | <0.05 |
| KIF4A | 2.17 | 1.86-2.53 | <0.05 |
| KIF7 | 3.26 | 2.59-4.1 | <0.05 |
| KIFC1 | 1.79 | 1.56-2.06 | <0.05 |
| KIR3DX1 | 1.72 | 1.54-1.93 | <0.05 |
| KLHDC1 | 0.5 | 0.38-0.64 | <0.05 |
| KLHDC8B | 0.59 | 0.47-0.74 | <0.05 |
| KLHL23 | 3 | 2.1-4.29 | <0.05 |
| KLHL6 | 0.65 | 0.55-0.77 | <0.05 |
| KLLN | 3.52 | 2.07-5.98 | <0.05 |
| KLRF2 | 1.44 | 1.27-1.64 | <0.05 |
| KPNA2 | 1.95 | 1.62-2.35 | <0.05 |
| KPNB1 | 2.79 | 2-3.88 | <0.05 |
| KRT80 | 4.41 | 2.83-6.86 | <0.05 |
| KRT81 | 2.23 | 1.64-3.04 | <0.05 |
| KRT86 | 2.88 | 1.95-4.24 | <0.05 |
| L1CAM | 20.33 | 7.58-54.55 | <0.05 |
| L2HGDH | 2.55 | 1.75-3.72 | <0.05 |
| LAMA5 | 0.64 | 0.56-0.74 | <0.05 |
| LAMP3 | 0.68 | 0.6-0.78 | <0.05 |
| LATS2 | 0.64 | 0.54-0.76 | <0.05 |
| LBR | 2.23 | 1.83-2.72 | <0.05 |
| LCLAT1 | 2.48 | 1.78-3.45 | <0.05 |
| LCP1 | 1.2 | 1.12-1.29 | <0.05 |
| LDHA | 1.81 | 1.49-2.19 | <0.05 |
| LEF1 | 1.36 | 1.2-1.54 | <0.05 |
| LGALS1 | 1.43 | 1.25-1.62 | <0.05 |
| LGALS3 | 1.37 | 1.2-1.57 | <0.05 |
| LIN7B | 0.43 | 0.34-0.55 | <0.05 |
| LMF1 | 0.53 | 0.46-0.62 | <0.05 |
| LMNB1 | 2.48 | 2.06-2.98 | <0.05 |
| LMNB2 | 2.18 | 1.7-2.8 | <0.05 |
| LMNTD2 | 0.57 | 0.45-0.72 | <0.05 |
| LMOD1 | 5.28 | 2.65-10.52 | <0.05 |
| LPGAT1 | 1.66 | 1.41-1.95 | <0.05 |
| LRP8 | 2.11 | 1.71-2.6 | <0.05 |
| LRR1 | 2.86 | 2.21-3.71 | <0.05 |
| LRRC16A | 0.68 | 0.58-0.8 | <0.05 |
| LRRC63 | 7.63 | 3.25-17.93 | <0.05 |
| LRRC66 | 2.93 | 1.85-4.65 | <0.05 |
| LSAMP | 0.79 | 0.71-0.86 | <0.05 |
| LSM 2.00 | 2.29 | 1.7-3.08 | <0.05 |
| LSM 6.00 | 2.18 | 1.56-3.04 | <0.05 |
| LTBP1 | 0.63 | 0.55-0.72 | <0.05 |
| LTBP3 | 0.62 | 0.53-0.72 | <0.05 |
| LY86 | 0.77 | 0.69-0.86 | <0.05 |
| LYAR | 2.16 | 1.7-2.75 | <0.05 |
| LYPD6B | 0.7 | 0.63-0.78 | <0.05 |
| LYPLA1 | 2.55 | 1.87-3.49 | <0.05 |
| LYRM9 | 0.33 | 0.21-0.51 | <0.05 |
| MAD2L1 | 2.3 | 1.95-2.73 | <0.05 |
| MAD2L2 | 2.01 | 1.53-2.65 | <0.05 |
| MAGEA1 | 1.36 | 1.27-1.46 | <0.05 |
| MAGEA12 | 1.29 | 1.16-1.43 | <0.05 |
| MAGEA2 | 251.31 | 26.93-2345.22 | <0.05 |
| MAGEA2B | 22477.12 | 713.49-708095.11 | <0.05 |
| MAGEA3 | 1.25 | 1.16-1.35 | <0.05 |
| MAGEA4 | 1.49 | 1.32-1.69 | <0.05 |
| MAGEA6 | 1.24 | 1.14-1.34 | <0.05 |
| MAGEA8 | 7.19 | 3.31-15.58 | <0.05 |
| MAGEB1 | 1.46 | 1.27-1.68 | <0.05 |
| MAGEB16 | 1.4 | 1.22-1.61 | <0.05 |
| MAGEB2 | 1.36 | 1.25-1.48 | <0.05 |
| MAGEB3 | 2.6 | 1.82-3.71 | <0.05 |
| MAK16 | 2.82 | 2.02-3.92 | <0.05 |
| MAN1B1 | 0.48 | 0.36-0.64 | <0.05 |
| MANBAL | 0.41 | 0.3-0.56 | <0.05 |
| MANSC1 | 0.69 | 0.59-0.81 | <0.05 |
| MAP1B | 1.52 | 1.32-1.75 | <0.05 |
| MAP3K12 | 1.67 | 1.35-2.06 | <0.05 |
| MAP7D3 | 2.16 | 1.65-2.82 | <0.05 |
| MAPRE1 | 2.67 | 1.92-3.73 | <0.05 |
| MARCH3 | 1.87 | 1.46-2.38 | <0.05 |
| MARCH8 | 0.38 | 0.29-0.49 | <0.05 |
| MAZ | 2.35 | 1.68-3.3 | <0.05 |
| MCFD2 | 0.4 | 0.3-0.53 | <0.05 |
| MCHR2 | 13.38 | 4.98-35.92 | <0.05 |
| MCIDAS | 9.69 | 5.87-15.98 | <0.05 |
| MCM10 | 2.77 | 2.3-3.33 | <0.05 |
| MCM2 | 1.94 | 1.7-2.21 | <0.05 |
| MCM3 | 2.14 | 1.81-2.54 | <0.05 |
| MCM4 | 2.28 | 1.95-2.65 | <0.05 |
| MCM5 | 2.36 | 1.92-2.9 | <0.05 |
| MCM6 | 2.28 | 1.93-2.7 | <0.05 |
| MCM7 | 2.09 | 1.66-2.63 | <0.05 |
| MCMBP | 2.56 | 1.83-3.56 | <0.05 |
| MCTP2 | 0.65 | 0.54-0.78 | <0.05 |
| MEI1 | 0.66 | 0.57-0.76 | <0.05 |
| MELK | 1.99 | 1.72-2.32 | <0.05 |
| MERTK | 0.7 | 0.63-0.79 | <0.05 |
| MEST | 1.51 | 1.27-1.79 | <0.05 |
| MEX3A | 2.54 | 1.81-3.58 | <0.05 |
| MFAP3L | 0.75 | 0.68-0.83 | <0.05 |
| MGAT1 | 0.51 | 0.38-0.68 | <0.05 |
| MGAT4A | 0.53 | 0.42-0.66 | <0.05 |
| MIIP | 2.29 | 1.77-2.97 | <0.05 |
| MIOX | 1.47 | 1.26-1.7 | <0.05 |
| MIS18A | 2.49 | 1.97-3.15 | <0.05 |
| MKI67 | 1.69 | 1.5-1.91 | <0.05 |
| MLLT11 | 1.51 | 1.28-1.77 | <0.05 |
| MMS22L | 4.4 | 3.03-6.4 | <0.05 |
| MND1 | 2.05 | 1.72-2.43 | <0.05 |
| MNX1 | 4.21 | 2.95-6.01 | <0.05 |
| MOB3C | 0.53 | 0.42-0.66 | <0.05 |
| MPC2 | 2.16 | 1.65-2.84 | <0.05 |
| MPHOSPH10 | 2.71 | 1.89-3.9 | <0.05 |
| MRPL12 | 1.75 | 1.4-2.2 | <0.05 |
| MRPL13 | 3.37 | 2.49-4.58 | <0.05 |
| MRPL14 | 2.08 | 1.6-2.71 | <0.05 |
| MRPL15 | 1.91 | 1.53-2.37 | <0.05 |
| MRPL19 | 2.27 | 1.65-3.14 | <0.05 |
| MRPL24 | 1.72 | 1.37-2.16 | <0.05 |
| MRPL28 | 2.48 | 1.77-3.48 | <0.05 |
| MRPL39 | 2.51 | 1.91-3.31 | <0.05 |
| MRPL47 | 2.25 | 1.69-2.99 | <0.05 |
| MRPL9 | 2.63 | 1.98-3.49 | <0.05 |
| MRPS18C | 2.66 | 1.78-3.97 | <0.05 |
| MRPS23 | 2.17 | 1.68-2.8 | <0.05 |
| MRPS24 | 0.58 | 0.46-0.73 | <0.05 |
| MRPS26 | 2.02 | 1.52-2.69 | <0.05 |
| MRPS35 | 2.17 | 1.65-2.84 | <0.05 |
| MRTO4 | 3.25 | 2.4-4.42 | <0.05 |
| MSH2 | 2.05 | 1.7-2.48 | <0.05 |
| MSH6 | 2.62 | 2-3.43 | <0.05 |
| MSN | 2.1 | 1.69-2.61 | <0.05 |
| MTBP | 5.19 | 3.38-7.97 | <0.05 |
| MTFR2 | 2.17 | 1.7-2.77 | <0.05 |
| MTHFD1 | 2.24 | 1.74-2.89 | <0.05 |
| MTHFD2 | 1.66 | 1.4-1.99 | <0.05 |
| MTIF3 | 0.6 | 0.49-0.74 | <0.05 |
| MTMR11 | 1.44 | 1.25-1.65 | <0.05 |
| MTUS1 | 0.59 | 0.5-0.7 | <0.05 |
| MTX1 | 2.29 | 1.72-3.05 | <0.05 |
| MUC13 | 1.35 | 1.19-1.54 | <0.05 |
| MUC15 | 1.38 | 1.22-1.56 | <0.05 |
| MYBL2 | 1.83 | 1.58-2.11 | <0.05 |
| MYBPC2 | 1.2 | 1.13-1.28 | <0.05 |
| MYH9 | 1.61 | 1.32-1.96 | <0.05 |
| MYLK | 1.46 | 1.26-1.7 | <0.05 |
| MYLK2 | 46.11 | 16.84-126.22 | <0.05 |
| MYO1C | 0.58 | 0.47-0.72 | <0.05 |
| MZF1 | 0.6 | 0.49-0.72 | <0.05 |
| NAA15 | 2.97 | 2.09-4.21 | <0.05 |
| NAPSA | 2.21 | 1.78-2.73 | <0.05 |
| NASP | 1.8 | 1.44-2.25 | <0.05 |
| NCALD | 1.48 | 1.27-1.73 | <0.05 |
| NCAPD2 | 1.84 | 1.45-2.34 | <0.05 |
| NCAPD3 | 1.94 | 1.53-2.45 | <0.05 |
| NCAPG | 2.16 | 1.85-2.52 | <0.05 |
| NCAPG2 | 1.86 | 1.58-2.18 | <0.05 |
| NCAPH | 2.17 | 1.86-2.53 | <0.05 |
| NCKAP1 | 0.64 | 0.53-0.78 | <0.05 |
| NCL | 3.15 | 2.21-4.47 | <0.05 |
| NCOA3 | 0.65 | 0.54-0.78 | <0.05 |
| NDC1 | 2.37 | 1.81-3.11 | <0.05 |
| NDC80 | 1.88 | 1.62-2.18 | <0.05 |
| NDE1 | 2.35 | 1.66-3.33 | <0.05 |
| NDST1 | 0.61 | 0.49-0.75 | <0.05 |
| NDUFB9 | 2.51 | 1.81-3.48 | <0.05 |
| NDUFS2 | 1.92 | 1.46-2.51 | <0.05 |
| NEIL3 | 3.45 | 2.65-4.48 | <0.05 |
| NEK2 | 1.92 | 1.67-2.21 | <0.05 |
| NFE4 | 1.38 | 1.21-1.56 | <0.05 |
| NIFK | 2.07 | 1.6-2.67 | <0.05 |
| NIP7 | 2.28 | 1.7-3.06 | <0.05 |
| NKX2.1 | 16560.25 | 687.65-398810.47 | <0.05 |
| NLRC3 | 1.89 | 1.57-2.29 | <0.05 |
| NLRP5 | 2.97 | 1.88-4.72 | <0.05 |
| NMU | 1.4 | 1.26-1.57 | <0.05 |
| NOBOX | 114.61 | 17.52-749.84 | <0.05 |
| NOC2L | 3.72 | 2.59-5.36 | <0.05 |
| NOL10 | 2.91 | 1.99-4.25 | <0.05 |
| NOLC1 | 2.23 | 1.63-3.06 | <0.05 |
| NONO | 2.98 | 2.23-3.97 | <0.05 |
| NOP16 | 1.83 | 1.45-2.32 | <0.05 |
| NOP56 | 2.65 | 2.02-3.47 | <0.05 |
| NOSTRIN | 4.15 | 2.56-6.74 | <0.05 |
| NOXA1 | 0.58 | 0.5-0.67 | <0.05 |
| NPAP1 | 2.15 | 1.59-2.91 | <0.05 |
| NPC2 | 0.66 | 0.57-0.76 | <0.05 |
| NPEPL1 | 0.52 | 0.4-0.68 | <0.05 |
| NR3C1 | 0.57 | 0.46-0.7 | <0.05 |
| NR6A1 | 1.95 | 1.54-2.46 | <0.05 |
| NRM | 1.99 | 1.61-2.46 | <0.05 |
| NRSN2 | 1.74 | 1.45-2.1 | <0.05 |
| NSDHL | 1.89 | 1.52-2.34 | <0.05 |
| NSL1 | 1.99 | 1.56-2.54 | <0.05 |
| NSMCE3 | 0.58 | 0.47-0.73 | <0.05 |
| NT5C1A | 12.87 | 4.89-33.9 | <0.05 |
| NTRK1 | 1.6 | 1.39-1.85 | <0.05 |
| NUB1 | 0.71 | 0.62-0.83 | <0.05 |
| NUCKS1 | 1.67 | 1.35-2.06 | <0.05 |
| NUDC | 2.77 | 1.92-4.01 | <0.05 |
| NUDCD1 | 2.67 | 1.99-3.58 | <0.05 |
| NUDT1 | 1.91 | 1.54-2.36 | <0.05 |
| NUDT11 | 1.45 | 1.24-1.7 | <0.05 |
| NUDT18 | 0.59 | 0.47-0.74 | <0.05 |
| NUF2 | 1.96 | 1.73-2.22 | <0.05 |
| NUP107 | 2.38 | 1.72-3.29 | <0.05 |
| NUP133 | 2.42 | 1.84-3.19 | <0.05 |
| NUP153 | 2.17 | 1.68-2.8 | <0.05 |
| NUP155 | 2.46 | 1.84-3.3 | <0.05 |
| NUP93 | 1.88 | 1.52-2.32 | <0.05 |
| NUSAP1 | 1.64 | 1.45-1.85 | <0.05 |
| NUTF2 | 2.46 | 1.7-3.58 | <0.05 |
| NVL | 1.96 | 1.47-2.61 | <0.05 |
| NXPE1 | 0.64 | 0.53-0.77 | <0.05 |
| NXPE4 | 0.78 | 0.72-0.84 | <0.05 |
| NXT2 | 1.81 | 1.51-2.18 | <0.05 |
| OIP5 | 2.13 | 1.8-2.52 | <0.05 |
| ORAI3 | 0.46 | 0.34-0.61 | <0.05 |
| ORC1 | 2.62 | 2.16-3.17 | <0.05 |
| ORC6 | 2.4 | 1.94-2.98 | <0.05 |
| OS9 | 0.37 | 0.27-0.51 | <0.05 |
| OSBPL10 | 0.63 | 0.55-0.71 | <0.05 |
| OSBPL6 | 2.66 | 1.89-3.76 | <0.05 |
| OSGIN2 | 1.91 | 1.47-2.49 | <0.05 |
| OTUD1 | 0.77 | 0.69-0.85 | <0.05 |
| OTUD6B | 2.02 | 1.5-2.72 | <0.05 |
| OXCT1 | 2.27 | 1.74-2.97 | <0.05 |
| P2RY1 | 1.72 | 1.43-2.05 | <0.05 |
| PA2G4 | 3.33 | 2.41-4.59 | <0.05 |
| PAGE2 | 1.4 | 1.25-1.56 | <0.05 |
| PAGE2B | 1.42 | 1.28-1.59 | <0.05 |
| PAGE4 | 1.57 | 1.32-1.87 | <0.05 |
| PAGE5 | 1.29 | 1.2-1.38 | <0.05 |
| PAIP2B | 0.62 | 0.53-0.72 | <0.05 |
| PALM3 | 7.93 | 4.53-13.86 | <0.05 |
| PALMD | 4.8 | 2.58-8.93 | <0.05 |
| PAM | 0.47 | 0.38-0.58 | <0.05 |
| PAQR4 | 2.1 | 1.75-2.52 | <0.05 |
| PARP1 | 1.77 | 1.41-2.22 | <0.05 |
| PASD1 | 1.73 | 1.39-2.15 | <0.05 |
| PBDC1 | 3.17 | 2.32-4.34 | <0.05 |
| PBK | 1.82 | 1.59-2.09 | <0.05 |
| PCBD1 | 0.51 | 0.41-0.63 | <0.05 |
| PCDH20 | 3.57 | 2.19-5.82 | <0.05 |
| PCDHB15 | 2.31 | 1.68-3.17 | <0.05 |
| PCED1B | 0.74 | 0.66-0.83 | <0.05 |
| PCGF5 | 0.53 | 0.42-0.67 | <0.05 |
| PCLAF | 1.92 | 1.64-2.25 | <0.05 |
| PCMTD1 | 0.64 | 0.53-0.77 | <0.05 |
| PCNA | 2.1 | 1.78-2.49 | <0.05 |
| PCSK6 | 2.4 | 1.71-3.36 | <0.05 |
| PCYOX1L | 1.9 | 1.58-2.28 | <0.05 |
| PDCD10 | 2.43 | 1.82-3.25 | <0.05 |
| PDCD11 | 2.04 | 1.57-2.67 | <0.05 |
| PDCL2 | 16.65 | 5.6-49.49 | <0.05 |
| PDCL3 | 1.68 | 1.39-2.04 | <0.05 |
| PDE3B | 1.67 | 1.44-1.93 | <0.05 |
| PDE4B | 0.71 | 0.63-0.8 | <0.05 |
| PDGFB | 2.25 | 1.63-3.1 | <0.05 |
| PDPK1 | 0.38 | 0.27-0.54 | <0.05 |
| PDSS1 | 2.88 | 2.24-3.71 | <0.05 |
| PECAM1 | 0.69 | 0.63-0.76 | <0.05 |
| PECR | 1.88 | 1.45-2.44 | <0.05 |
| PELO | 1.94 | 1.46-2.56 | <0.05 |
| PEMT | 2.03 | 1.55-2.65 | <0.05 |
| PES 1.00 | 2.86 | 2.05-3.97 | <0.05 |
| PEX5L | 1.95 | 1.55-2.44 | <0.05 |
| PEX7 | 0.51 | 0.38-0.68 | <0.05 |
| PFDN2 | 3.13 | 2.43-4.02 | <0.05 |
| PFDN4 | 1.95 | 1.52-2.51 | <0.05 |
| PFDN6 | 2.13 | 1.56-2.91 | <0.05 |
| PFKP | 1.6 | 1.42-1.8 | <0.05 |
| PFN1 | 2.29 | 1.77-2.95 | <0.05 |
| PGAM1 | 2.61 | 1.74-3.91 | <0.05 |
| PGAM5 | 2.33 | 1.65-3.3 | <0.05 |
| PGK 1.00 | 2.15 | 1.57-2.96 | <0.05 |
| PHB | 2 | 1.55-2.59 | <0.05 |
| PHF1 | 0.54 | 0.43-0.68 | <0.05 |
| PHF11 | 0.54 | 0.42-0.7 | <0.05 |
| PHF19 | 2.07 | 1.81-2.38 | <0.05 |
| PHF5A | 2.33 | 1.65-3.28 | <0.05 |
| PIK3CA | 0.45 | 0.33-0.62 | <0.05 |
| PIN4 | 2.25 | 1.59-3.18 | <0.05 |
| PIP5K1B | 0.71 | 0.62-0.82 | <0.05 |
| PITX1 | 1.33 | 1.18-1.49 | <0.05 |
| PKDCC | 1.8 | 1.52-2.13 | <0.05 |
| PKMYT1 | 2.25 | 1.89-2.68 | <0.05 |
| PKP2 | 1.29 | 1.18-1.41 | <0.05 |
| PLCB1 | 1.78 | 1.42-2.23 | <0.05 |
| PLCG2 | 0.61 | 0.51-0.73 | <0.05 |
| PLEC | 1.51 | 1.28-1.77 | <0.05 |
| PLK1 | 2.31 | 1.97-2.72 | <0.05 |
| PLK4 | 2.48 | 2.07-2.97 | <0.05 |
| PLPP3 | 0.66 | 0.56-0.77 | <0.05 |
| PLPP5 | 0.58 | 0.48-0.7 | <0.05 |
| PMF1 | 2.49 | 1.87-3.32 | <0.05 |
| PNPLA7 | 0.6 | 0.51-0.72 | <0.05 |
| POC1A | 2.06 | 1.7-2.5 | <0.05 |
| POGK | 1.8 | 1.41-2.31 | <0.05 |
| POLA1 | 2.82 | 2.2-3.62 | <0.05 |
| POLA2 | 2.16 | 1.66-2.82 | <0.05 |
| POLD1 | 2.83 | 2.23-3.6 | <0.05 |
| POLD3 | 2.58 | 1.98-3.37 | <0.05 |
| POLD4 | 0.54 | 0.43-0.69 | <0.05 |
| POLDIP3 | 2.94 | 1.95-4.45 | <0.05 |
| POLE | 2.43 | 1.86-3.18 | <0.05 |
| POLE2 | 2.48 | 2.03-3.03 | <0.05 |
| POLE4 | 2.61 | 1.97-3.47 | <0.05 |
| POLQ | 2.54 | 2.1-3.06 | <0.05 |
| POLR2B | 2.33 | 1.65-3.3 | <0.05 |
| POLR2D | 2.18 | 1.56-3.02 | <0.05 |
| POLR3C | 2.39 | 1.77-3.24 | <0.05 |
| PON1 | 17.48 | 6.95-43.98 | <0.05 |
| POP1 | 3.24 | 2.34-4.48 | <0.05 |
| POP7 | 2.32 | 1.69-3.17 | <0.05 |
| POTEE | 8.54 | 4.01-18.19 | <0.05 |
| POU6F1 | 0.5 | 0.39-0.63 | <0.05 |
| POU6F2 | 3.63 | 2.47-5.34 | <0.05 |
| PPAT | 1.94 | 1.48-2.54 | <0.05 |
| PPCDC | 0.66 | 0.56-0.78 | <0.05 |
| PPFIBP2 | 0.46 | 0.36-0.6 | <0.05 |
| PPIH | 2.97 | 2.16-4.07 | <0.05 |
| PPIP5K1 | 0.54 | 0.42-0.7 | <0.05 |
| PPP1R37 | 0.44 | 0.32-0.6 | <0.05 |
| PPP1R8 | 3.15 | 2.08-4.78 | <0.05 |
| PPP1R9A | 1.63 | 1.33-1.98 | <0.05 |
| PPP2R5A | 1.85 | 1.44-2.37 | <0.05 |
| PPP3R1 | 2.36 | 1.67-3.33 | <0.05 |
| PPP4C | 3.55 | 2.47-5.09 | <0.05 |
| PRC1 | 1.64 | 1.38-1.96 | <0.05 |
| PRCC | 2.1 | 1.67-2.65 | <0.05 |
| PRDM13 | 3.96 | 2.55-6.16 | <0.05 |
| PRIM1 | 2.14 | 1.75-2.62 | <0.05 |
| PRIM2 | 3.84 | 2.86-5.15 | <0.05 |
| PRKAG2 | 0.43 | 0.32-0.57 | <0.05 |
| PRKCA | 0.67 | 0.59-0.77 | <0.05 |
| PRKCSH | 0.47 | 0.35-0.64 | <0.05 |
| PRKDC | 2.38 | 1.86-3.06 | <0.05 |
| PRPS1 | 1.75 | 1.39-2.21 | <0.05 |
| PRPS2 | 1.65 | 1.38-1.98 | <0.05 |
| PRR11 | 2.01 | 1.71-2.36 | <0.05 |
| PRSS2 | 1.5 | 1.31-1.72 | <0.05 |
| PRSS38 | 1.68 | 1.43-1.98 | <0.05 |
| PSAP | 0.47 | 0.38-0.58 | <0.05 |
| PSMA3 | 2.16 | 1.58-2.95 | <0.05 |
| PSMA6 | 2.53 | 1.83-3.5 | <0.05 |
| PSMA7 | 2.56 | 1.82-3.6 | <0.05 |
| PSMB2 | 2.9 | 2.09-4.04 | <0.05 |
| PSMB3 | 3.03 | 2.19-4.2 | <0.05 |
| PSMB4 | 2.36 | 1.77-3.15 | <0.05 |
| PSMC2 | 2.2 | 1.6-3.02 | <0.05 |
| PSMC3IP | 2.24 | 1.8-2.79 | <0.05 |
| PSMC5 | 2.43 | 1.69-3.51 | <0.05 |
| PSMD1 | 2.46 | 1.75-3.46 | <0.05 |
| PSMD11 | 2.89 | 2.03-4.1 | <0.05 |
| PSMD12 | 2.17 | 1.58-2.98 | <0.05 |
| PSMD13 | 2.46 | 1.69-3.56 | <0.05 |
| PSMD14 | 2.42 | 1.82-3.23 | <0.05 |
| PSMD4 | 2.32 | 1.83-2.95 | <0.05 |
| PSME3 | 2.75 | 1.87-4.04 | <0.05 |
| PSMG1 | 1.98 | 1.49-2.64 | <0.05 |
| PSMG2 | 2.36 | 1.67-3.33 | <0.05 |
| PSPH | 1.69 | 1.39-2.05 | <0.05 |
| PSRC1 | 2.2 | 1.7-2.85 | <0.05 |
| PTBP1 | 4.12 | 2.56-6.62 | <0.05 |
| PTEN | 2.69 | 1.94-3.75 | <0.05 |
| PTGES3 | 2.33 | 1.63-3.34 | <0.05 |
| PTMA | 3.03 | 2.19-4.19 | <0.05 |
| PTPN13 | 1.47 | 1.26-1.71 | <0.05 |
| PTTG1 | 1.57 | 1.37-1.8 | <0.05 |
| PUF60 | 4.59 | 3.34-6.32 | <0.05 |
| PWWP2B | 0.62 | 0.52-0.75 | <0.05 |
| PXMP2 | 1.93 | 1.53-2.44 | <0.05 |
| QPRT | 0.72 | 0.63-0.82 | <0.05 |
| QRFPR | 29.8 | 7.34-120.92 | <0.05 |
| RAB11B | 0.46 | 0.33-0.64 | <0.05 |
| RAB13 | 0.67 | 0.58-0.77 | <0.05 |
| RAB17 | 2.2 | 1.58-3.05 | <0.05 |
| RAB33A | 1.31 | 1.18-1.46 | <0.05 |
| RAB37 | 1.31 | 1.17-1.46 | <0.05 |
| RABIF | 1.76 | 1.38-2.23 | <0.05 |
| RAC3 | 2.49 | 1.92-3.23 | <0.05 |
| RACGAP1 | 1.99 | 1.69-2.35 | <0.05 |
| RAD18 | 3.67 | 2.67-5.03 | <0.05 |
| RAD21 | 2.9 | 2.22-3.77 | <0.05 |
| RAD23B | 3.09 | 2.07-4.63 | <0.05 |
| RAD51 | 2.54 | 2.11-3.07 | <0.05 |
| RAD51AP1 | 1.98 | 1.68-2.34 | <0.05 |
| RAD51C | 2.71 | 2-3.67 | <0.05 |
| RAD54B | 7.33 | 3.88-13.84 | <0.05 |
| RAD54L | 2.46 | 2.03-2.99 | <0.05 |
| RAD9B | 5.67 | 3-10.72 | <0.05 |
| RAE1 | 3.09 | 2.24-4.27 | <0.05 |
| RALGPS1 | 0.53 | 0.42-0.66 | <0.05 |
| RAN | 2.95 | 2.2-3.95 | <0.05 |
| RANBP1 | 3.23 | 2.38-4.38 | <0.05 |
| RASAL3 | 1.73 | 1.43-2.1 | <0.05 |
| RASSF8 | 0.51 | 0.39-0.67 | <0.05 |
| RBBP8 | 2.32 | 1.86-2.9 | <0.05 |
| RBFOX2 | 0.52 | 0.42-0.65 | <0.05 |
| RBM15 | 2.11 | 1.6-2.79 | <0.05 |
| RBM34 | 2.42 | 1.7-3.43 | <0.05 |
| RBM6 | 0.51 | 0.4-0.65 | <0.05 |
| RBM8A | 3.41 | 2.58-4.52 | <0.05 |
| RBX1 | 2.67 | 1.93-3.69 | <0.05 |
| RCBTB2 | 0.73 | 0.65-0.82 | <0.05 |
| RCC1 | 2.11 | 1.66-2.68 | <0.05 |
| RCC2 | 2.01 | 1.67-2.42 | <0.05 |
| RDM1 | 11.27 | 5.46-23.23 | <0.05 |
| RECQL | 2.07 | 1.58-2.72 | <0.05 |
| RECQL4 | 2.12 | 1.81-2.47 | <0.05 |
| REEP1 | 1.96 | 1.52-2.54 | <0.05 |
| REEP4 | 2.09 | 1.62-2.7 | <0.05 |
| REEP5 | 0.37 | 0.28-0.49 | <0.05 |
| RETNLB | 3.75 | 2.27-6.2 | <0.05 |
| RFC2 | 2.42 | 1.85-3.16 | <0.05 |
| RFC3 | 2.02 | 1.67-2.44 | <0.05 |
| RFC4 | 2.57 | 2.04-3.24 | <0.05 |
| RFC5 | 2.68 | 2.1-3.43 | <0.05 |
| RGAG1 | 141.62 | 19.11-1049.73 | <0.05 |
| RGS6 | 4.74 | 2.87-7.84 | <0.05 |
| RHBDD1 | 0.46 | 0.34-0.61 | <0.05 |
| RHEBL1 | 1.8 | 1.42-2.29 | <0.05 |
| RHOC | 1.46 | 1.29-1.65 | <0.05 |
| RHOXF2B | 8.1 | 3.68-17.84 | <0.05 |
| RIBC2 | 1.82 | 1.43-2.31 | <0.05 |
| RIPOR2 | 1.3 | 1.17-1.44 | <0.05 |
| RMDN3 | 0.49 | 0.38-0.63 | <0.05 |
| RMI1 | 1.78 | 1.4-2.25 | <0.05 |
| RMI2 | 1.96 | 1.62-2.36 | <0.05 |
| RNASEH2A | 2.31 | 1.86-2.86 | <0.05 |
| RNF11 | 0.67 | 0.56-0.79 | <0.05 |
| RNF115 | 2.79 | 1.89-4.13 | <0.05 |
| RNF125 | 1.66 | 1.38-2.01 | <0.05 |
| RNF139 | 2.02 | 1.51-2.7 | <0.05 |
| RNF141 | 0.45 | 0.33-0.61 | <0.05 |
| RNPS1 | 2.91 | 1.94-4.38 | <0.05 |
| RP11.579D7.1 | 6.78 | 4.3-10.68 | <0.05 |
| RP2 | 1.86 | 1.43-2.43 | <0.05 |
| RP3.412A9.11 | 0.66 | 0.59-0.74 | <0.05 |
| RPL26L1 | 2.42 | 1.7-3.43 | <0.05 |
| RPP40 | 1.87 | 1.5-2.31 | <0.05 |
| RPS27L | 0.5 | 0.37-0.67 | <0.05 |
| RPS6KA1 | 1.94 | 1.57-2.4 | <0.05 |
| RPS6KA4 | 2.1 | 1.55-2.83 | <0.05 |
| RQCD1 | 3.1 | 2.19-4.39 | <0.05 |
| RRM1 | 2.19 | 1.77-2.71 | <0.05 |
| RRM2 | 1.47 | 1.33-1.63 | <0.05 |
| RRP15 | 2.4 | 1.78-3.22 | <0.05 |
| RRP1B | 2 | 1.5-2.67 | <0.05 |
| RRP7A | 2.29 | 1.65-3.16 | <0.05 |
| RTKN2 | 5.37 | 2.91-9.91 | <0.05 |
| RUNX2 | 1.39 | 1.28-1.52 | <0.05 |
| RWDD2A | 0.56 | 0.44-0.71 | <0.05 |
| RYK | 0.41 | 0.3-0.58 | <0.05 |
| SAE1 | 2.17 | 1.64-2.89 | <0.05 |
| SAP30 | 1.91 | 1.49-2.45 | <0.05 |
| SAPCD1 | 2.34 | 1.74-3.14 | <0.05 |
| SASS6 | 2.36 | 1.75-3.18 | <0.05 |
| SAT2 | 0.42 | 0.33-0.54 | <0.05 |
| SCAMP5 | 0.68 | 0.62-0.76 | <0.05 |
| SCGB3A2 | 4.91 | 2.65-9.09 | <0.05 |
| SCLY | 11.19 | 4.23-29.6 | <0.05 |
| SCNM1 | 2.41 | 1.81-3.2 | <0.05 |
| SCRIB | 2.21 | 1.62-3.01 | <0.05 |
| SCRN1 | 0.67 | 0.58-0.77 | <0.05 |
| SCYL2 | 0.67 | 0.58-0.77 | <0.05 |
| SDHB | 3 | 2.02-4.45 | <0.05 |
| SEC62 | 0.55 | 0.43-0.7 | <0.05 |
| SECISBP2 | 0.47 | 0.35-0.63 | <0.05 |
| SEH1L | 2.28 | 1.62-3.2 | <0.05 |
| SEL1L3 | 0.69 | 0.62-0.78 | <0.05 |
| SEMA3C | 2.45 | 1.79-3.37 | <0.05 |
| SEMA3D | 1.7 | 1.44-2.02 | <0.05 |
| SEMA4A | 1.33 | 1.18-1.49 | <0.05 |
| SEMA5B | 85.39 | 17.98-405.53 | <0.05 |
| SENP1 | 3.03 | 2.02-4.52 | <0.05 |
| SEPT11 | 2 | 1.65-2.42 | <0.05 |
| SERBP1 | 1.98 | 1.53-2.56 | <0.05 |
| SERPINB6 | 0.78 | 0.7-0.86 | <0.05 |
| SF3A3 | 4.11 | 2.67-6.34 | <0.05 |
| SF3B2 | 2.4 | 1.72-3.35 | <0.05 |
| SF3B4 | 2.28 | 1.8-2.89 | <0.05 |
| SF3B6 | 2.63 | 1.98-3.49 | <0.05 |
| SFMBT2 | 0.56 | 0.46-0.68 | <0.05 |
| SGCB | 1.37 | 1.21-1.56 | <0.05 |
| SGO1 | 2.89 | 2.32-3.6 | <0.05 |
| SGO2 | 2.42 | 1.95-3 | <0.05 |
| SH2D2A | 2.11 | 1.79-2.49 | <0.05 |
| SH3BP1 | 1.71 | 1.43-2.05 | <0.05 |
| SH3GL2 | 15.58 | 5.93-40.96 | <0.05 |
| SH3GLB2 | 0.57 | 0.45-0.72 | <0.05 |
| SH3RF1 | 1.74 | 1.47-2.05 | <0.05 |
| SH3RF3 | 1.36 | 1.23-1.49 | <0.05 |
| SHCBP1 | 1.77 | 1.54-2.03 | <0.05 |
| SHISA3 | 1.51 | 1.28-1.77 | <0.05 |
| SHROOM3 | 1.61 | 1.44-1.81 | <0.05 |
| SIPA1L3 | 0.52 | 0.41-0.65 | <0.05 |
| SIX1 | 1.86 | 1.47-2.36 | <0.05 |
| SIX5 | 0.58 | 0.47-0.71 | <0.05 |
| SKA1 | 2.03 | 1.77-2.32 | <0.05 |
| SKA3 | 2.27 | 1.93-2.66 | <0.05 |
| SLA | 1.5 | 1.34-1.67 | <0.05 |
| SLAMF7 | 0.59 | 0.5-0.7 | <0.05 |
| SLBP | 2.59 | 2.01-3.33 | <0.05 |
| SLC16A1 | 1.76 | 1.42-2.17 | <0.05 |
| SLC19A1 | 1.72 | 1.41-2.09 | <0.05 |
| SLC1A7 | 23.21 | 8.75-61.59 | <0.05 |
| SLC22A25 | 8.72 | 4.04-18.81 | <0.05 |
| SLC22A9 | 1.52 | 1.33-1.73 | <0.05 |
| SLC25A10 | 2.81 | 2.07-3.8 | <0.05 |
| SLC25A21 | 2.64 | 1.99-3.5 | <0.05 |
| SLC25A23 | 0.62 | 0.53-0.73 | <0.05 |
| SLC25A33 | 2.91 | 2.13-3.98 | <0.05 |
| SLC25A45 | 0.61 | 0.51-0.74 | <0.05 |
| SLC25A53 | 3.24 | 2.25-4.68 | <0.05 |
| SLC38A1 | 1.73 | 1.4-2.14 | <0.05 |
| SLC43A3 | 1.74 | 1.52-1.98 | <0.05 |
| SLC46A3 | 0.73 | 0.64-0.83 | <0.05 |
| SLC47A1 | 0.8 | 0.73-0.88 | <0.05 |
| SLC4A11 | 1.71 | 1.42-2.07 | <0.05 |
| SLC52A2 | 1.84 | 1.44-2.37 | <0.05 |
| SLC7A7 | 0.69 | 0.6-0.79 | <0.05 |
| SLC9A5 | 3.54 | 2.15-5.83 | <0.05 |
| SLFNL1 | 8.91 | 3.65-21.75 | <0.05 |
| SLIT1 | 0.47 | 0.36-0.61 | <0.05 |
| SMARCE1 | 2.91 | 1.92-4.4 | <0.05 |
| SMC1A | 2.11 | 1.63-2.74 | <0.05 |
| SMC1B | 2.13 | 1.62-2.8 | <0.05 |
| SMC2 | 2.08 | 1.7-2.55 | <0.05 |
| SMC4 | 1.69 | 1.37-2.09 | <0.05 |
| SMCO2 | 54.63 | 16.89-176.75 | <0.05 |
| SMG5 | 2.11 | 1.55-2.87 | <0.05 |
| SMIM12 | 2.33 | 1.63-3.32 | <0.05 |
| SMIM13 | 2.2 | 1.62-2.98 | <0.05 |
| SNN | 0.68 | 0.58-0.8 | <0.05 |
| SNRNP40 | 3.75 | 2.48-5.68 | <0.05 |
| SNRNP70 | 0.64 | 0.54-0.77 | <0.05 |
| SNRPB | 3.6 | 2.75-4.71 | <0.05 |
| SNRPB2 | 2.47 | 1.77-3.44 | <0.05 |
| SNRPC | 3.8 | 2.65-5.45 | <0.05 |
| SNRPD1 | 3.13 | 2.37-4.12 | <0.05 |
| SNRPD3 | 3.11 | 2.21-4.36 | <0.05 |
| SNRPE | 2.3 | 1.82-2.9 | <0.05 |
| SNRPF | 3.05 | 2.1-4.43 | <0.05 |
| SNRPG | 2.8 | 2.12-3.68 | <0.05 |
| SNTB2 | 2.15 | 1.63-2.82 | <0.05 |
| SNW1 | 2.66 | 1.85-3.82 | <0.05 |
| SNX24 | 0.54 | 0.41-0.7 | <0.05 |
| SNX33 | 0.46 | 0.34-0.61 | <0.05 |
| SOAT2 | 1.83 | 1.51-2.22 | <0.05 |
| SOCS6 | 1.54 | 1.3-1.82 | <0.05 |
| SOD1 | 2.14 | 1.59-2.87 | <0.05 |
| SOHLH1 | 1.33 | 1.21-1.46 | <0.05 |
| SOX11 | 2.54 | 1.94-3.32 | <0.05 |
| SOX9 | 1.87 | 1.48-2.37 | <0.05 |
| SP140 | 0.6 | 0.5-0.72 | <0.05 |
| SPA17 | 3.46 | 2.15-5.55 | <0.05 |
| SPAG5 | 2.16 | 1.83-2.55 | <0.05 |
| SPANXB1 | 5.79 | 2.98-11.24 | <0.05 |
| SPANXD | 19.7 | 5.61-69.22 | <0.05 |
| SPATA17 | 6.95 | 3.53-13.67 | <0.05 |
| SPATS2 | 0.49 | 0.38-0.65 | <0.05 |
| SPC24 | 1.86 | 1.6-2.17 | <0.05 |
| SPC25 | 2.04 | 1.76-2.36 | <0.05 |
| SPDL1 | 2.54 | 1.94-3.33 | <0.05 |
| SPIB | 1.77 | 1.48-2.12 | <0.05 |
| SPIN4 | 1.93 | 1.48-2.53 | <0.05 |
| SPRED1 | 1.4 | 1.22-1.6 | <0.05 |
| SPRR2A | 1.51 | 1.36-1.68 | <0.05 |
| SPRR2B | 230.15 | 33.99-1558.14 | <0.05 |
| SPRR2D | 1.39 | 1.25-1.54 | <0.05 |
| SPRTN | 2.97 | 2.06-4.26 | <0.05 |
| SPRY4 | 2.31 | 1.86-2.87 | <0.05 |
| SPTBN2 | 0.76 | 0.68-0.84 | <0.05 |
| SPTSSA | 1.95 | 1.5-2.53 | <0.05 |
| SRPK1 | 1.96 | 1.49-2.57 | <0.05 |
| SRPK2 | 1.81 | 1.41-2.31 | <0.05 |
| SRSF3 | 2.24 | 1.6-3.14 | <0.05 |
| SS18L2 | 2.12 | 1.53-2.92 | <0.05 |
| SSBP1 | 2.35 | 1.64-3.37 | <0.05 |
| SSPN | 0.59 | 0.49-0.71 | <0.05 |
| SSRP1 | 2.71 | 1.82-4.03 | <0.05 |
| SSX1 | 1.27 | 1.17-1.37 | <0.05 |
| SSX2B | 111.94 | 18.3-684.83 | <0.05 |
| SSX4 | 19.22 | 5.45-67.73 | <0.05 |
| SSX4B | 62.92 | 14.1-280.76 | <0.05 |
| SSX7 | 3.5 | 2.06-5.95 | <0.05 |
| ST3GAL1 | 1.28 | 1.15-1.42 | <0.05 |
| ST3GAL5 | 0.64 | 0.53-0.77 | <0.05 |
| ST6GAL1 | 0.64 | 0.54-0.75 | <0.05 |
| ST6GALNAC5 | 13.15 | 5.98-28.96 | <0.05 |
| ST7 | 0.38 | 0.27-0.55 | <0.05 |
| ST8SIA2 | 5.94 | 2.92-12.12 | <0.05 |
| STAC | 9.16 | 4.55-18.42 | <0.05 |
| STAP1 | 0.78 | 0.72-0.85 | <0.05 |
| STARD5 | 0.56 | 0.48-0.66 | <0.05 |
| STAU2 | 2.94 | 2.08-4.15 | <0.05 |
| STC2 | 1.36 | 1.22-1.52 | <0.05 |
| STEAP1 | 1.44 | 1.29-1.61 | <0.05 |
| STIL | 2.94 | 2.31-3.74 | <0.05 |
| STIM1 | 1.76 | 1.39-2.22 | <0.05 |
| STK26 | 1.93 | 1.56-2.4 | <0.05 |
| STK38 | 1.62 | 1.32-1.99 | <0.05 |
| STMN1 | 1.8 | 1.58-2.05 | <0.05 |
| STRA13 | 2.31 | 1.76-3.04 | <0.05 |
| STRIP2 | 2.24 | 1.78-2.81 | <0.05 |
| SUMO1 | 2.99 | 2.1-4.25 | <0.05 |
| SUMO2 | 2.86 | 1.91-4.27 | <0.05 |
| SUPT16H | 2.49 | 1.88-3.29 | <0.05 |
| SUV39H1 | 3.27 | 2.52-4.24 | <0.05 |
| SUV39H2 | 2.13 | 1.61-2.82 | <0.05 |
| SUZ12 | 3.84 | 2.69-5.48 | <0.05 |
| SYT14 | 27.96 | 6.83-114.49 | <0.05 |
| SZRD1 | 2.43 | 1.74-3.39 | <0.05 |
| TACC3 | 2.14 | 1.79-2.56 | <0.05 |
| TADA1 | 2.64 | 1.98-3.52 | <0.05 |
| TAF15 | 2.29 | 1.66-3.17 | <0.05 |
| TAF1B | 2.38 | 1.68-3.38 | <0.05 |
| TAF2 | 3.86 | 2.61-5.71 | <0.05 |
| TAF5L | 2.33 | 1.79-3.02 | <0.05 |
| TAGAP | 0.69 | 0.61-0.79 | <0.05 |
| TAGLN2 | 1.5 | 1.36-1.65 | <0.05 |
| TAPBPL | 0.59 | 0.48-0.72 | <0.05 |
| TARS2 | 2.1 | 1.61-2.72 | <0.05 |
| TBC1D31 | 3.77 | 2.56-5.54 | <0.05 |
| TBC1D9 | 0.6 | 0.52-0.69 | <0.05 |
| TBX20 | 517.39 | 50.3-5321.93 | <0.05 |
| TBX22 | 2.04 | 1.56-2.66 | <0.05 |
| TCF19 | 1.61 | 1.4-1.85 | <0.05 |
| TCF7L2 | 0.43 | 0.3-0.61 | <0.05 |
| TERF1 | 2.88 | 1.84-4.49 | <0.05 |
| TES | 1.74 | 1.39-2.18 | <0.05 |
| TESK2 | 0.51 | 0.4-0.65 | <0.05 |
| TESPA1 | 2.19 | 1.62-2.95 | <0.05 |
| TEX101 | 1.29 | 1.16-1.42 | <0.05 |
| TEX30 | 2.18 | 1.68-2.83 | <0.05 |
| TFAM | 2.52 | 1.75-3.64 | <0.05 |
| TFB2M | 2.08 | 1.6-2.71 | <0.05 |
| TFDP1 | 1.64 | 1.36-1.97 | <0.05 |
| TGS1 | 2.21 | 1.68-2.91 | <0.05 |
| THOP1 | 2.26 | 1.66-3.09 | <0.05 |
| THY1 | 1.33 | 1.2-1.48 | <0.05 |
| TICRR | 3.11 | 2.52-3.83 | <0.05 |
| TIMELESS | 1.7 | 1.42-2.03 | <0.05 |
| TIMM17A | 2.03 | 1.6-2.58 | <0.05 |
| TIMM23 | 2.74 | 1.94-3.87 | <0.05 |
| TIMM8A | 2.4 | 1.92-3 | <0.05 |
| TIPIN | 2.58 | 1.98-3.37 | <0.05 |
| TIPRL | 2.67 | 2.05-3.46 | <0.05 |
| TJP1 | 0.72 | 0.65-0.79 | <0.05 |
| TK1 | 1.93 | 1.69-2.2 | <0.05 |
| TLE6 | 2.49 | 1.8-3.45 | <0.05 |
| TLK1 | 0.52 | 0.4-0.67 | <0.05 |
| TLL1 | 1263.59 | 168.26-9489.19 | <0.05 |
| TLR1 | 0.55 | 0.45-0.66 | <0.05 |
| TM7SF2 | 0.54 | 0.43-0.69 | <0.05 |
| TMC6 | 1.27 | 1.17-1.37 | <0.05 |
| TMC8 | 1.33 | 1.22-1.44 | <0.05 |
| TMEM106C | 1.87 | 1.53-2.3 | <0.05 |
| TMEM164 | 1.75 | 1.44-2.12 | <0.05 |
| TMEM167B | 0.49 | 0.37-0.65 | <0.05 |
| TMEM169 | 15.22 | 5.3-43.69 | <0.05 |
| TMEM183A | 2.65 | 1.93-3.65 | <0.05 |
| TMEM219 | 0.41 | 0.3-0.58 | <0.05 |
| TMEM241 | 2.11 | 1.57-2.83 | <0.05 |
| TMEM243 | 0.56 | 0.44-0.71 | <0.05 |
| TMEM43 | 1.84 | 1.45-2.33 | <0.05 |
| TMEM59 | 0.53 | 0.42-0.67 | <0.05 |
| TMEM97 | 1.83 | 1.53-2.19 | <0.05 |
| TMOD3 | 2.22 | 1.59-3.1 | <0.05 |
| TMPO | 2.08 | 1.67-2.59 | <0.05 |
| TMPPE | 2.55 | 1.73-3.75 | <0.05 |
| TMPRSS5 | 0.34 | 0.22-0.52 | <0.05 |
| TMSB15A | 1.62 | 1.43-1.82 | <0.05 |
| TNFRSF10A | 1.7 | 1.36-2.13 | <0.05 |
| TNFRSF8 | 1.96 | 1.56-2.46 | <0.05 |
| TNFSF13 | 0.58 | 0.47-0.73 | <0.05 |
| TNFSF9 | 1.48 | 1.3-1.68 | <0.05 |
| TNIP1 | 0.59 | 0.49-0.73 | <0.05 |
| TOMM40 | 2.4 | 1.71-3.36 | <0.05 |
| TOMM40L | 2.05 | 1.54-2.74 | <0.05 |
| TOMM7 | 0.55 | 0.43-0.69 | <0.05 |
| TONSL | 2.6 | 2.09-3.24 | <0.05 |
| TOP2A | 1.67 | 1.49-1.88 | <0.05 |
| TOP3A | 2.46 | 1.7-3.56 | <0.05 |
| TOPBP1 | 2.74 | 2.08-3.63 | <0.05 |
| TPGS2 | 2.75 | 2.05-3.69 | <0.05 |
| TPI1 | 2.13 | 1.62-2.79 | <0.05 |
| TPM3 | 2.23 | 1.83-2.71 | <0.05 |
| TPRKB | 2.28 | 1.67-3.12 | <0.05 |
| TPX2 | 1.72 | 1.52-1.94 | <0.05 |
| TRAF3 | 0.66 | 0.57-0.77 | <0.05 |
| TRAF3IP3 | 1.45 | 1.27-1.64 | <0.05 |
| TRAIP | 2.75 | 2.19-3.46 | <0.05 |
| TREML2 | 1.25 | 1.14-1.36 | <0.05 |
| TRIAP1 | 2.32 | 1.67-3.22 | <0.05 |
| TRIB3 | 1.37 | 1.21-1.54 | <0.05 |
| TRIM11 | 2.78 | 2.01-3.83 | <0.05 |
| TRIM46 | 2.05 | 1.7-2.47 | <0.05 |
| TRIM71 | 3.92 | 2.3-6.68 | <0.05 |
| TRIOBP | 4.02 | 2.33-6.94 | <0.05 |
| TRIP13 | 2.3 | 1.97-2.67 | <0.05 |
| TRMT6 | 2.26 | 1.63-3.15 | <0.05 |
| TRNAU1AP | 2.02 | 1.54-2.66 | <0.05 |
| TROAP | 2.01 | 1.73-2.35 | <0.05 |
| TRPS1 | 2.01 | 1.59-2.53 | <0.05 |
| TRPT1 | 0.42 | 0.32-0.57 | <0.05 |
| TSEN15 | 1.91 | 1.57-2.33 | <0.05 |
| TSFM | 2.77 | 2-3.84 | <0.05 |
| TSN | 2.46 | 1.68-3.61 | <0.05 |
| TSPAN12 | 1.44 | 1.26-1.64 | <0.05 |
| TSPAN31 | 0.34 | 0.23-0.49 | <0.05 |
| TSTA3 | 1.89 | 1.46-2.45 | <0.05 |
| TTBK1 | 11.23 | 4.07-30.97 | <0.05 |
| TTC13 | 2.1 | 1.6-2.77 | <0.05 |
| TTC29 | 11.93 | 4.6-30.93 | <0.05 |
| TTK | 2.22 | 1.86-2.66 | <0.05 |
| TTLL4 | 2.46 | 1.77-3.4 | <0.05 |
| TUBA1B | 2.49 | 2.03-3.05 | <0.05 |
| TUBA1C | 2.33 | 1.86-2.92 | <0.05 |
| TUBA3C | 1.4 | 1.21-1.62 | <0.05 |
| TUBB | 2.06 | 1.7-2.5 | <0.05 |
| TUBG1 | 2.05 | 1.65-2.55 | <0.05 |
| TWIST1 | 1.45 | 1.24-1.71 | <0.05 |
| TYMS | 1.69 | 1.5-1.92 | <0.05 |
| UBA2 | 2.73 | 1.84-4.06 | <0.05 |
| UBA7 | 0.7 | 0.61-0.79 | <0.05 |
| UBAP2L | 2.34 | 1.76-3.11 | <0.05 |
| UBE2C | 1.76 | 1.56-1.99 | <0.05 |
| UBE2J2 | 2.83 | 1.83-4.36 | <0.05 |
| UBE2L3 | 2.87 | 1.89-4.37 | <0.05 |
| UBE2S | 1.91 | 1.5-2.42 | <0.05 |
| UBE2T | 1.83 | 1.6-2.09 | <0.05 |
| UBE2V2 | 2.57 | 1.87-3.52 | <0.05 |
| UBQLN4 | 1.82 | 1.41-2.34 | <0.05 |
| UBR7 | 2.02 | 1.5-2.71 | <0.05 |
| UCHL5 | 2.05 | 1.55-2.72 | <0.05 |
| UHMK1 | 1.61 | 1.31-1.97 | <0.05 |
| UHRF1 | 2.13 | 1.82-2.51 | <0.05 |
| UMPS | 2.01 | 1.53-2.66 | <0.05 |
| UNC119 | 0.44 | 0.35-0.57 | <0.05 |
| UNC13C | 4.37 | 3.04-6.28 | <0.05 |
| UNC13D | 0.62 | 0.52-0.72 | <0.05 |
| UNC93B1 | 0.57 | 0.48-0.67 | <0.05 |
| UNG | 1.78 | 1.46-2.19 | <0.05 |
| URB2 | 2.64 | 2.02-3.44 | <0.05 |
| USF2 | 0.39 | 0.29-0.54 | <0.05 |
| USP1 | 1.96 | 1.52-2.54 | <0.05 |
| USP13 | 1.74 | 1.4-2.15 | <0.05 |
| USP31 | 2.72 | 1.86-3.98 | <0.05 |
| USP37 | 3.55 | 2.34-5.41 | <0.05 |
| USP39 | 2.68 | 1.77-4.04 | <0.05 |
| UTP11 | 4.09 | 2.8-5.97 | <0.05 |
| UTP14A | 1.92 | 1.5-2.46 | <0.05 |
| UTP18 | 2.26 | 1.64-3.11 | <0.05 |
| UTP23 | 2.6 | 1.82-3.72 | <0.05 |
| UTP3 | 1.68 | 1.39-2.04 | <0.05 |
| UTP6 | 2.63 | 1.85-3.75 | <0.05 |
| VAMP2 | 0.53 | 0.41-0.68 | <0.05 |
| VAMP7 | 1.68 | 1.35-2.09 | <0.05 |
| VBP1 | 1.71 | 1.38-2.13 | <0.05 |
| VCPIP1 | 2.07 | 1.6-2.68 | <0.05 |
| VCX | 1.72 | 1.39-2.12 | <0.05 |
| VCX2 | 1.59 | 1.35-1.88 | <0.05 |
| VCX3A | 1.94 | 1.53-2.46 | <0.05 |
| VILL | 0.5 | 0.38-0.65 | <0.05 |
| VMA21 | 1.86 | 1.46-2.38 | <0.05 |
| VPS25 | 2.41 | 1.72-3.38 | <0.05 |
| VPS4B | 2.19 | 1.63-2.95 | <0.05 |
| VSIG1 | 1.54 | 1.29-1.85 | <0.05 |
| VSIR | 0.61 | 0.51-0.72 | <0.05 |
| WBP2 | 0.48 | 0.35-0.65 | <0.05 |
| WDHD1 | 2.95 | 2.36-3.7 | <0.05 |
| WDR43 | 3.06 | 2.31-4.04 | <0.05 |
| WDR62 | 2.41 | 1.96-2.96 | <0.05 |
| WDR72 | 1.61 | 1.37-1.89 | <0.05 |
| WDR75 | 2.77 | 1.93-3.96 | <0.05 |
| WDR76 | 1.9 | 1.6-2.26 | <0.05 |
| WEE1 | 1.61 | 1.44-1.82 | <0.05 |
| WNK4 | 13.58 | 6.98-26.43 | <0.05 |
| WNT4 | 0.56 | 0.47-0.67 | <0.05 |
| WNT9A | 2.57 | 2.03-3.24 | <0.05 |
| XAGE1A | 12.39 | 5.09-30.17 | <0.05 |
| XAGE1B | 8.61 | 3.88-19.09 | <0.05 |
| XK | 1.54 | 1.32-1.78 | <0.05 |
| XPO1 | 2.06 | 1.53-2.77 | <0.05 |
| XRCC2 | 2.11 | 1.77-2.52 | <0.05 |
| XRCC3 | 2.69 | 1.93-3.75 | <0.05 |
| XRCC5 | 2.97 | 2.09-4.21 | <0.05 |
| XRCC6 | 3.91 | 2.63-5.8 | <0.05 |
| YARS | 1.93 | 1.46-2.54 | <0.05 |
| YBX1 | 2.56 | 1.82-3.6 | <0.05 |
| YEATS4 | 2.35 | 1.71-3.23 | <0.05 |
| YIF1B | 2.31 | 1.72-3.1 | <0.05 |
| YTHDF3 | 2.32 | 1.65-3.26 | <0.05 |
| YWHAZ | 2.05 | 1.57-2.67 | <0.05 |
| ZBED4 | 3.02 | 2.08-4.37 | <0.05 |
| ZBP1 | 0.64 | 0.53-0.76 | <0.05 |
| ZBTB32 | 1.36 | 1.19-1.54 | <0.05 |
| ZBTB4 | 0.46 | 0.38-0.57 | <0.05 |
| ZC3H12D | 0.58 | 0.47-0.71 | <0.05 |
| ZC3H15 | 2.65 | 1.82-3.86 | <0.05 |
| ZC3H3 | 2.02 | 1.51-2.7 | <0.05 |
| ZCCHC18 | 3.54 | 2.2-5.68 | <0.05 |
| ZFAND4 | 0.52 | 0.4-0.67 | <0.05 |
| ZHX2 | 0.46 | 0.37-0.57 | <0.05 |
| ZNF124 | 2.94 | 1.92-4.49 | <0.05 |
| ZNF208 | 2.1 | 1.6-2.74 | <0.05 |
| ZNF215 | 0.68 | 0.59-0.78 | <0.05 |
| ZNF257 | 2.74 | 1.89-3.98 | <0.05 |
| ZNF267 | 1.83 | 1.47-2.28 | <0.05 |
| ZNF300 | 1.37 | 1.2-1.56 | <0.05 |
| ZNF358 | 0.62 | 0.53-0.73 | <0.05 |
| ZNF367 | 1.93 | 1.62-2.29 | <0.05 |
| ZNF486 | 2.1 | 1.58-2.79 | <0.05 |
| ZNF534 | 10.06 | 4.3-23.56 | <0.05 |
| ZNF541 | 14.65 | 5.78-37.15 | <0.05 |
| ZNF554 | 0.37 | 0.25-0.55 | <0.05 |
| ZNF555 | 0.41 | 0.28-0.58 | <0.05 |
| ZNF578 | 15.94 | 5.67-44.79 | <0.05 |
| ZNF580 | 0.58 | 0.47-0.73 | <0.05 |
| ZNF710 | 0.55 | 0.43-0.7 | <0.05 |
| ZNF714 | 2.41 | 1.75-3.31 | <0.05 |
| ZNF716 | 4.9 | 3.07-7.84 | <0.05 |
| ZNF724P | 3.07 | 1.96-4.81 | <0.05 |
| ZNF726 | 3.72 | 2.55-5.44 | <0.05 |
| ZNF738 | 1.93 | 1.49-2.5 | <0.05 |
| ZNF763 | 0.31 | 0.2-0.47 | <0.05 |
| ZNF775 | 0.55 | 0.44-0.68 | <0.05 |
| ZNF823 | 2.36 | 1.68-3.29 | <0.05 |
| ZNF837 | 0.44 | 0.32-0.62 | <0.05 |
| ZNF844 | 0.65 | 0.55-0.78 | <0.05 |
| ZNF85 | 1.87 | 1.48-2.37 | <0.05 |
| ZNF860 | 0.65 | 0.55-0.76 | <0.05 |
| ZNF90 | 3.29 | 2.14-5.07 | <0.05 |
| ZNF93 | 2.69 | 1.89-3.83 | <0.05 |
| ZSCAN26 | 0.41 | 0.3-0.57 | <0.05 |
| ZWILCH | 2.15 | 1.68-2.76 | <0.05 |
| ZWINT | 1.74 | 1.53-1.98 | <0.05 |
| AC004383.4 | 1.41 | 1.26-1.57 | <0.05 |
| AC005592.2 | 64.69 | 22.92-182.64 | <0.05 |
| AC005624.2 | 4.7 | 2.82-7.81 | <0.05 |
| AC007040.5 | 5.48 | 2.67-11.24 | <0.05 |
| AC007240.1 | 2.6 | 2.01-3.35 | <0.05 |
| AC007246.3 | 0.29 | 0.19-0.44 | <0.05 |
| AC009410.1 | 1.28 | 1.16-1.42 | <0.05 |
| AC010226.4 | 0.51 | 0.39-0.68 | <0.05 |
| AC019066.3 | 0.76 | 0.68-0.84 | <0.05 |
| AC066606.1 | 13.6 | 4.57-40.47 | <0.05 |
| AC068491.1 | 1.82 | 1.55-2.13 | <0.05 |
| AC073236.2 | 1.25 | 1.15-1.37 | <0.05 |
| AC092669.3 | 42.88 | 11.67-157.53 | <0.05 |
| AC093734.11 | 0.49 | 0.36-0.66 | <0.05 |
| AC097724.3 | 5.25 | 2.92-9.44 | <0.05 |
| AC098973.2 | 1.33 | 1.18-1.51 | <0.05 |
| AC108142.1 | 37.88 | 9.63-148.99 | <0.05 |
| AC108488.3 | 2.13 | 1.69-2.67 | <0.05 |
| AC129492.6 | 4.26 | 2.6-6.98 | <0.05 |
| AC135893.2 | 2.4 | 1.79-3.23 | <0.05 |
| AL157902.3 | 0.35 | 0.22-0.53 | <0.05 |
| AP000251.3 | 2.84 | 2.12-3.81 | <0.05 |
| CH507.154B10.2 | 1180.05 | 158.38-8792.4 | <0.05 |
| CTC.470E21.2 | 0.38 | 0.28-0.52 | <0.05 |
| CTC.523E23.4 | 1.94 | 1.49-2.53 | <0.05 |
| CTD.2008L17.2 | 2.65 | 2.07-3.38 | <0.05 |
| CTD.3035D6.2 | 2.03 | 1.55-2.67 | <0.05 |
| CTD.3049M7.1 | 10.84 | 4.03-29.11 | <0.05 |
| DSCR8 | 1.39 | 1.23-1.58 | <0.05 |
| FOXD2.AS1 | 2.95 | 2.14-4.06 | <0.05 |
| GS1.120K12.4 | 5572464.29 | 8710.56-3564908722.21 | <0.05 |
| HCG23 | 0.38 | 0.26-0.55 | <0.05 |
| LINC00461 | 2.51 | 1.72-3.66 | <0.05 |
| LINC00668 | 4.18 | 2.52-6.92 | <0.05 |
| LL0XNC01.116E7.2 | 4.57 | 2.65-7.88 | <0.05 |
| MIR600HG | 0.64 | 0.54-0.77 | <0.05 |
| MNX1.AS1 | 1.55 | 1.33-1.81 | <0.05 |
| NAPA.AS1 | 0.41 | 0.3-0.57 | <0.05 |
| PCGEM1 | 18970.38 | 367.56-979102.74 | <0.05 |
| PWRN1 | 2.31 | 1.68-3.18 | <0.05 |
| RP1.20N18.6 | 63.34 | 15.52-258.54 | <0.05 |
| RP11.1058B10.1 | 9.96 | 4.49-22.06 | <0.05 |
| RP11.108M21.1 | 2.91 | 2.15-3.94 | <0.05 |
| RP11.1103G16.1 | 2.66 | 2-3.53 | <0.05 |
| RP11.127L20.3 | 0.56 | 0.45-0.69 | <0.05 |
| RP11.15B17.1 | 104.38 | 25.15-433.15 | <0.05 |
| RP11.161H23.9 | 106332 | 3412.68-3313083.35 | <0.05 |
| RP11.171A24.2 | 2633.38 | 92.8-74730.68 | <0.05 |
| RP11.178A10.1 | 1.63 | 1.38-1.92 | <0.05 |
| RP11.17H4.2 | 0.76 | 0.68-0.86 | <0.05 |
| RP11.181C3.2 | 2.62 | 2-3.45 | <0.05 |
| RP11.199O14.1 | 3.76 | 2.26-6.25 | <0.05 |
| RP11.1L12.3 | 1.61 | 1.33-1.95 | <0.05 |
| RP11.21C4.1 | 10.62 | 5.73-19.67 | <0.05 |
| RP11.227D13.1 | 5.08 | 2.85-9.05 | <0.05 |
| RP11.22P6.3 | 2.41 | 1.95-2.99 | <0.05 |
| RP11.253M7.1 | 1895.44 | 203.81-17627.95 | <0.05 |
| RP11.276H19.2 | 1.77 | 1.45-2.17 | <0.05 |
| RP11.278A23.1 | 8.26 | 4.21-16.2 | <0.05 |
| RP11.29H23.1 | 1.56 | 1.29-1.88 | <0.05 |
| RP11.305L7.1 | 1.62 | 1.37-1.91 | <0.05 |
| RP11.305N23.1 | 6.45 | 3.66-11.35 | <0.05 |
| RP11.30E17.3 | 0.42 | 0.3-0.61 | <0.05 |
| RP11.326A19.4 | 2.91 | 2.22-3.81 | <0.05 |
| RP11.328K4.1 | 1.46 | 1.26-1.7 | <0.05 |
| RP11.32K4.1 | 2.51 | 1.95-3.23 | <0.05 |
| RP11.345M22.1 | 1.76 | 1.46-2.13 | <0.05 |
| RP11.35L17.2 | 3.44 | 2.3-5.13 | <0.05 |
| RP11.366F6.2 | 2.9 | 2.02-4.15 | <0.05 |
| RP11.367G18.1 | 0.77 | 0.68-0.86 | <0.05 |
| RP11.379B18.6 | 15.79 | 5.75-43.36 | <0.05 |
| RP11.392A22.2 | 0.46 | 0.35-0.6 | <0.05 |
| RP11.411D10.1 | 7.39 | 3.4-16.06 | <0.05 |
| RP11.434C1.1 | 0.52 | 0.41-0.67 | <0.05 |
| RP11.438N16.1 | 1.66 | 1.38-1.99 | <0.05 |
| RP11.456O19.2 | 7.71 | 4.45-13.36 | <0.05 |
| RP11.456O19.4 | 333.07 | 31.89-3478.75 | <0.05 |
| RP11.497E19.1 | 1.33E+12 | 1.22E+04-1.45E+24 | <0.05 |
| RP11.500C11.3 | 2.04 | 1.57-2.65 | <0.05 |
| RP11.502I4.1 | 0.52 | 0.41-0.67 | <0.05 |
| RP11.513O17.2 | 1.41 | 1.22-1.62 | <0.05 |
| RP11.527L4.2 | 2.7 | 1.77-4.11 | <0.05 |
| RP11.528B10.4 | 2.55 | 1.81-3.59 | <0.05 |
| RP11.549K20.1 | 1.72 | 1.39-2.14 | <0.05 |
| RP11.551L14.5 | 3.61 | 2.24-5.82 | <0.05 |
| RP11.579E24.1 | 1.3 | 1.17-1.45 | <0.05 |
| RP11.580I1.3 | 2.27 | 1.69-3.05 | <0.05 |
| RP11.58A17.4 | 3.62 | 2.19-5.97 | <0.05 |
| RP11.600K15.1 | 17.34 | 7.53-39.92 | <0.05 |
| RP11.617F23.1 | 0.56 | 0.46-0.68 | <0.05 |
| RP11.697M17.1 | 1.32 | 1.18-1.48 | <0.05 |
| RP11.723G8.2 | 4.43 | 2.39-8.22 | <0.05 |
| RP11.758M4.1 | 2.8 | 1.96-4 | <0.05 |
| RP11.76C10.5 | 1.71 | 1.41-2.08 | <0.05 |
| RP11.776H12.1 | 1.85 | 1.49-2.29 | <0.05 |
| RP11.782E2.1 | 2.77 | 1.94-3.95 | <0.05 |
| RP11.791G22.1 | 15.92 | 5.69-44.55 | <0.05 |
| RP11.806L2.3 | 2.36 | 1.88-2.98 | <0.05 |
| RP11.81H3.2 | 1.57 | 1.33-1.86 | <0.05 |
| RP11.84C10.4 | 6.75 | 3.31-13.78 | <0.05 |
| RP11.9G1.3 | 4.4 | 2.41-8.05 | <0.05 |
| RP3.398D13.3 | 0.48 | 0.35-0.66 | <0.05 |
| RP4.655J12.4 | 61467.34 | 1361.94-2774158.66 | <0.05 |
| RP4.754E20__A.5 | 3.53 | 2.31-5.39 | <0.05 |
| RP5.1121A15.4 | 4.95 | 2.92-8.4 | <0.05 |
| RP5.1166F10.1 | 150.85 | 18.76-1212.93 | <0.05 |
| RP5.1171I10.4 | 2.44 | 1.75-3.41 | <0.05 |
| SAMMSON | 53.97 | 16.29-178.86 | <0.05 |
| ST3GAL4.AS1 | 6.79 | 4.03-11.45 | <0.05 |
| TSPEAR.AS2 | 1.54 | 1.32-1.8 | <0.05 |
| TTTY9B | 1.26E+238 | 2.21E+138-Inf | <0.05 |
| MIR206 | 4.23 | 2.34-7.63 | <0.05 |
| MIR3122 | 2.52 | 1.96-3.25 | <0.05 |
| MIR3648.1 | 52.76 | 13.4-207.8 | <0.05 |
| MIR3671 | 0.7 | 0.61-0.82 | <0.05 |
| MIR4506 | 1.69 | 1.37-2.08 | <0.05 |
| MIR503 | 1.73 | 1.41-2.12 | <0.05 |
| MIR515.1 | 5.64E+12 | 2.56E+07-1.24E+18 | <0.05 |
| MIR548AA1 | 1.67 | 1.43-1.96 | <0.05 |
| MIR5680 | 9.84 | 4.31-22.47 | <0.05 |
| MIR6074 | 1010.14 | 62.24-16394.36 | <0.05 |
| MIR6783 | 3.9 | 2.4-6.31 | <0.05 |
| MIR7158 | 1.34 | 1.19-1.5 | <0.05 |
| MIR7848 | 2.04 | 1.62-2.55 | <0.05 |
