# Supplementary material for: Systematic discrimination of the repetitive genome in proximity of ferroptosis genes and a novel prognostic signature correlating with the oncogenic lncRNA CRNDE in multiple myeloma
Source: Front Oncol. 2022 Dec 20;12:1026153. doi: 10.3389/fonc.2022.1026153 (PMC9808058; doi:10.3389/fonc.2022.1026153)
Supplement: Supplementary file 14 [file Table_3.docx]

**Supplementary table 3. Seventeen ferroptosis-related potential prognostic genes generated from the training cohort.**

| Gene | Description | P (KM) | Univariate cox analysis | | | |
| --- | --- | --- | --- | --- | --- | --- |
|  |  |  | HR | CI95_L | CI95_H | P |
| SLC38A1 | solute carrier family 38 member 1 | <0.0001 | 1.73 | 1.4 | 2.14 | <0.0001 |
| ALOX12B | arachidonate 12-lipoxygenase, 12R type | 0.0098 | 28.67 | 7.88 | 104.33 | <0.0001 |
| CDKN2A | cyclin dependent kinase inhibitor 2A | <0.0001 | 2.28 | 1.83 | 2.84 | <0.0001 |
| MIOX | myo-inositol oxygenase | 0.00019 | 1.47 | 1.26 | 1.7 | <0.0001 |
| AGPS | alkylglycerone phosphate synthase | 0.00046 | 3.82 | 2.65 | 5.52 | <0.0001 |
| PIK3CA | phosphatidylinositol-4,5-bisphosphate 3-kinase catalytic subunit alpha | <0.0001 | 0.45 | 0.33 | 0.62 | <0.0001 |
| HELLS | helicase, lymphoid specific | <0.0001 | 2.86 | 3.57 | 3.57 | <0.0001 |
| FH | fumarate hydratase | 0.0004 | 2.19 | 1.74 | 2.76 | <0.0001 |
| ISCU | iron-sulfur cluster assembly enzyme | 0.00098 | 0.51 | 0.4 | 0.65 | <0.0001 |
| DAZAP1 | DAZ associated protein 1 | <0.0001 | 5.26 | 3.19 | 8.68 | <0.0001 |
| SLC16A1 | solute carrier family 16 member 1 | 0.0026 | 1.76 | 1.42 | 2.17 | <0.0001 |
| RRM2 | Ribonucleotide reductase regulatory subunit M2 | <0.0001 | 1.47 | 1.33 | 1.63 | <0.0001 |
| SUV39H1 | SUV39H1 histone lysine methyltransferase | <0.0001 | 3.27 | 2.52 | 4.24 | <0.0001 |
| CDCA3 | cell division cycle associated 3 | 0.0015 | 2.16 | 1.76 | 2.67 | <0.0001 |
| DDIT4 | DNA damage inducible transcript 4 | <0.0001 | 1.37 | 1.24 | 1.52 | <0.0001 |
| GPT2 | glutamic--pyruvic transaminase 2 | 0.00011 | 1.38 | 1.21 | 1.59 | <0.0001 |
| TRIB3 | tribbles pseudokinase 3 | <0.0001 | 1.37 | 1.21 | 1.54 | <0.0001 |
