# Supplementary material for: Systematic discrimination of the repetitive genome in proximity of ferroptosis genes and a novel prognostic signature correlating with the oncogenic lncRNA CRNDE in multiple myeloma
Source: Front Oncol. 2022 Dec 20;12:1026153. doi: 10.3389/fonc.2022.1026153 (PMC9808058; doi:10.3389/fonc.2022.1026153)
Supplement: Supplementary file 15 [file Table_4.docx]

**Supplementary table 4.** Fourteen ferroptosis-related prognotic genes obtained from LASSO Cox regression model.

| Genes | Gene ID | Description | Annotation | Risk Coefficient |
| --- | --- | --- | --- | --- |
| SLC38A1 | 81539 | solute carrier family 38 member 1 | Ferroptosis driver | 0.1885 |
| ALOX12B | 242 | arachidonate 12-lipoxygenase, 12R type | Ferroptosis driver | 1.0223 |
| CDKN2A | 1029 | cyclin dependent kinase inhibitor 2A | Ferroptosis driver | 0.3994 |
| MIOX | 55586 | myo-inositol oxygenase | Ferroptosis driver | 0.1424 |
| AGPS | 8540 | alkylglycerone phosphate synthase | Ferroptosis driver | 0.1644 |
| PIK3CA | 5290 | phosphatidylinositol-4,5-bisphosphate 3-kinase catalytic subunit alpha | Ferroptosis driver | -0.4861 |
| HELLS | 3070 | helicase, lymphoid specific | Ferroptosis suppressor | 0.2649 |
| FH | 2271 | fumarate hydratase | Ferroptosis suppressor | 0.1245 |
| ISCU | 23479 | iron-sulfur cluster assembly enzyme | Ferroptosis suppressor | -0.0428 |
| DAZAP1 | 26528 | DAZ associated protein 1 | Ferroptosis suppressor | 0.1706 |
| SLC16A1 | 6566 | solute carrier family 16 member 1 | Ferroptosis suppressor | 0.1046 |
| SUV39H1 | 6839 | SUV39H1 histone lysine methyltransferase | Ferroptosis suppressor | 0.1882 |
| DDIT4 | 54541 | DNA damage inducible transcript 4 | Ferroptosis maker | 0.1898 |
| TRIB3 | 57761 | tribbles pseudokinase 3 | Ferroptosis maker | 0.0256 |
