# Supplementary material for: Systematic discrimination of the repetitive genome in proximity of ferroptosis genes and a novel prognostic signature correlating with the oncogenic lncRNA CRNDE in multiple myeloma
Source: Front Oncol. 2022 Dec 20;12:1026153. doi: 10.3389/fonc.2022.1026153 (PMC9808058; doi:10.3389/fonc.2022.1026153)
Supplement: Supplementary file 16 [file Table_5.docx]

**Supplementary table 5. Univariate analysis and multivariate analysis of the correlation of gene-signature risk score with outcomes among multiple myeloma patients in two cohorts including 1400 patients.**

| Characteristics | HR (U) | CI95 (U) | P (U) | HR (M) | CI95 (M) | P (M) |
| --- | --- | --- | --- | --- | --- | --- |
| MMRF-COMMPASS (OS) |  |  |  |  |  |  |
| Age | 1.03 | 1.02-1.05 | <0.001 | 1.03 | 1.02-1.05 | <0.001 |
| Ethnicity | 1.04 | 0.62-1.73 | 0.893 |  |  |  |
| Gender | 1.38 | 1.03-1.87 | 0.032 | 1.29 | 0.95-1.73 | 0.099 |
| Race | 0.76 | 0.54-1.08 | 0.124 | 0.79 | 0.56-1.12 | 0.181 |
| ISS stage (II vs I) | 2.07 | 1.34-2.80 | 0.001 | 1.65 | 1.06-2.56 | 0.026 |
| ISS stage (III vs I) | 4.21 | 3.19-6.33 | <0.001 | 2.59 | 1.70-3.94 | <0.001 |
| Signature | 1.18 | 1.15-1.21 | <0.001 | 1.17 | 1.13-1.20 | <0.001 |
| GSE24080 (OS) |  |  |  |  |  |  |
| Age | 1.02 | 1.01-1.04 | 0.005 | 1.01 | 1.00-1.03 | 0.112 |
| Albumin | 0.95 | 0.93-0.97 | <0.001 | 0.96 | 0.93-0.98 | 0.001 |
| B2M | 1.08 | 1.07-1.10 | <0.001 | 1.07 | 1.04-1.11 | <0.001 |
| Creatinine | 1.23 | 1.14-1.34 | <0.001 | 0.95 | 0.84-1.08 | 0.465 |
| CRP | 1.004 | 1.00-1.01 | 0.196 | 1.002 | 0.99-1.00 | 0.458 |
| Hemoglobin | 0.87 | 0.80-0.95 | 0.001 | 1.00 | 0.91-1.11 | 0.953 |
| Isotype (IgG vs others) | 0.93 | 0.69-1.26 | 0.65 |  |  |  |
| ISS stage (II vs I) | 1.57 | 1.07-2.02 | 0.02 | 1.01 | 0.66-1.54 | 0.967 |
| ISS stage (III vs I) | 2.87 | 2.30-4.07 | <0.001 | 0.94 | 0.54-1.63 | 0.821 |
| LDH | 1.01 | 1.00-1.01 | <0.001 | 1.009 | 1.00-1.01 | <0.001 |
| Race | 1.05 | 0.65-1.69 | 0.853 |  |  |  |
| Gender | 0.95 | 0.7-1.29 | 0.739 |  |  |  |
| Signature | 1.1 | 1.06-1.15 | <0.001 | 1.06 | 1.02-1.1 | 0.005 |
| GSE24080 (EFS) |  |  |  |  |  |  |
| Age | 1.01 | 1.00-1.03 | 0.057 | 1.00 | 0.99-1.02 | 0.726 |
| Albumin | 0.96 | 0.95-0.98 | <0.001 | 0.98 | 0.96-1 | 0.051 |
| B2M | 1.07 | 1.06-1.09 | <0.001 | 1.06 | 1.03-1.09 | <0.001 |
| Creatinine | 1.23 | 1.14-1.32 | <0.001 | 0.98 | 0.87-1.1 | 0.739 |
| CRP | 1.01 | 1-1.01 | 0.013 | 1.00 | 1.00-1.01 | 0.741 |
| Hemoglobin | 0.88 | 0.82-0.94 | <0.001 | 0.95 | 0.88-1.04 | 0.287 |
| Isotype (IgG vs others) | 1.04 | 0.78-1.29 | 0.995 |  |  |  |
| ISS stage (II vs I) | 1.39 | 1.75-1.9 | 0.036 | 0.97 | 0.69-1.39 | 0.888 |
| ISS stage (III vs I) | 2.35 | 1.9-3.16 | <0.001 | 0.93 | 0.59-1.47 | 0.748 |
| LDH | 1.01 | 1.00-1.01 | <0.001 | 1.008 | 1.00-1.01 | <0.001 |
| Race | 1.46 | 0.94-2.27 | 0.089 | 1.48 | 0.94-2.34 | 0.092 |
| Gender | 0.98 | 0.76-1.26 | 0.854 |  |  |  |
| Signature | 1.08 | 1.05-1.12 | <0.001 | 1.05 | 1.02-1.09 | 0.003 |

(U: univariate cox analysis; M: multivariate cox analysis; Only univariate P value less than 0.2 will be applied to multivariate cox analysis, and multivate P value less than 0.1 will be assigned in nomogram.)
